# Biomimicry in French Urban Projects: Trends and Perspectives from the Practice

**DOI:** 10.3390/biomimetics6020027

**Published:** 2021-04-27

**Authors:** Eduardo Blanco, Estelle Cruz, Chloé Lequette, Kalina Raskin, Philippe Clergeau

**Affiliations:** 1 Centre d’Écologie et des Sciences de la Conservation (CESCO UMR7204), MNHN, CNRS, Sorbonne Université, BP 135, 57 rue Cuvier, 75005 Paris, France; philippe.clergeau@mnhn.fr; 2 Ceebios, 62 rue du Faubourg Saint-Martin, 60300 Senlis, France; estelle.cruz@ceebios.com (E.C.); chloe.lequette@ceebios.com (C.L.); kalina.raskin@ceebios.com (K.R.)

**Keywords:** biomimicry, architecture, urban design, French urban projects

## Abstract

Biomimicry is a design framework with growing interests in sustainable architectural and urban design practice. Nevertheless, there is a significant lack of studies and knowledge regarding its practical application. In 2020, a French workgroup called Biomim’City Lab published a document identifying and describing 16 urban projects designed by French teams integrating biomimicry at various levels. Our research is an opportunistic study analyzing this data, aiming to identify trends and challenges in the French market. We analyzed the projects using a mixed-method approach, through quantitative typological analysis and qualitative narrative analysis. This sample of French projects indicates a trend of increasing interest in biomimicry on built space projects in France. Biomimicry was primarily applied at the façade/roof/soil systems, mostly using macroscopic models as ecosystems, plants, and animals. Designers declared to aim diverse objectives with the biomimetic approach; still, thermal comfort is the most recurrent in the sample. We also identified that challenges remain to foster the field application, as the lack of awareness of the urban fabric stakeholders on the topic and the gaps between research and design practice.

## 1. Introduction

Biomimicry draws upon emulation of, and knowledge transfer from, living organisms and whole ecosystems to find solutions to human problems [1]. In the built environment sciences, it is a growing topic [2,3,4], with an application that mainly aims for sustainable innovation [3,5]. Biomimicry offers an opportunity to operationalize sustainability and regenerative development on architectural and urban projects [2,6]. 

The practice of biomimetic architecture faced the first increase throughout the nineties, within the beginning of a global context of energy transition, giving birth to iconic biomimetic projects like the Eastgate building (1996) [2,5]. With the simultaneous development of biomimetic architecture in research, education, and architectural practice, the last two decades presented a surge of interest in the topic [7].

In Europe, biomimicry has been highlighted by the European Commission as an opportunity for research and innovation, with potential contributions to tackle climate change adaptation and mitigation [8]. Furthermore, some European research institutions focus on advancing the theory and practice of biomimicry in architectural and urban design. In Germany, the Universities of Stuttgart, Freiburg, and Tübingen have jointly dedicated efforts to develop and test biomimicry applications through the trans-regional Collaborative Research Center “Biological Design and Integrative Structures” SFB-TRR 141 [9]. In France, the French National History Museum created in 2019 the Bioinspire-Museum project to coordinate and promote bioinspiration throughout its activities, highlighting architectural and urban design applications of their biological and ecological knowledge [10]. In the Netherlands TU Delft has specialized teaching in the topic and hosted relevant research regarding biomimetic architectural design [11].

In France, biomimicry is a growing field of innovation. Since 2015 local stakeholders organize a national congress on biomimicry (Biomim’Expo), to showcase national emerging projects and research on the topic. In architecture and urban design, a collaborative workgroup of biomimicry practitioners was launched in 2019, called Biomim’City Lab. This collective, animated by Ceebios (a not-for-profit French network and center of expertise on biomimicry), integrates ten different French stakeholders of the urban fabric, as architects, real estate developers, consulting companies, and research institutions (in alphabetical order: Bechu & Associés, Ceebios, Eiffage, Elan, Icade, In Situ Architecture, New Corp Conseil, Nobatek/INEF 4, Renault, and Tangam Architectes). They all have experimented at some level biomimicry in their projects, aiming at sustainable development. This workgroup focus on increasing skills, co-developing new tools, and sharing expertise within the French context.

In 2020, one year after its creation, the Biomim’City Lab published an online report called “*Projets urbains bio-inspirés: un état des lieux des projets français*” (Bio-inspired urban projects: an inventory of French projects, available only in French in the Supplementary Materials). This report documented, in standard factsheets, sixteen French projects that have integrated biomimicry and bio-inspired approaches at different levels on urban projects dating from 2007 to 2019. The report presents the collected data, results, and feedback from the design teams from a sample of bio-inspired projects in architecture and urban design in France. The report is a communication and awareness-raising tool for practitioners to enhance biomimicry development in France [12].

The report is different from any other previous scientific and non-scientific publications regarding its objectives and data. Previously published thematic reports had only a few projects briefly presented [13]. In the academic field, few studies documented and explored the practice of biomimicry [11,14,15]. Hayes et al. (2019) realized one of the few available cases studies, focusing on six system-level biomimicry urban projects, but none of them from a European context. Furthermore, the analyzed projects in their study were not identified either deeply described. 

The sixteen documented projects from the “Bio-inspired urban projects: an inventory of French projects” report were selected by the BiomimCity’Lab stakeholders, following each stakeholder own preferences. The only criteria among all projects were that they had to integrate at some level a biomimetic approach and be situated in France or be designed by French teams. As a result, the analyzed projects vary in form, size, objective, status, and biomimicry application level. Furthermore, the report is not an exhaustive study of the French biomimetic urban projects, but it constituted an unprecedented sample worth analyzing. Several documented projects are proposals for architectural and urban design competitions, and the report includes projects that were not successful in the process. 

As a significant lack of studies and knowledge regarding biomimicry’s design practice applied to architectural and urban designs remains [16], our research carried out an opportunistic analysis of this French biomimetic practice sample, relying on all of the sixteen projects factsheets produced by the Biomim’City Lab. This study is an opportunity to through lights on the contemporary trends in the practice and challenges that design teams face integrating biomimicry.

In this context, the research questions we have explored are:

- What types of urban biomimetic projects did French teams design?
- How was the biomimetic approach expressed in these projects in terms of goals, biological models and integration level?
- What challenges did the design teams face on the design process?

## 2. Materials and Methods

### 2.1. Data Collection

As an opportunistic study, this research did not produced data, but data from the sixteen biomimetic French urban projects presented in the source document (“Projets urbains bio-inspirés: un état des lieux des projets français”, Biomim’City Lab, Paris, France, Document S1) were extracted and analyzed. All the French case studies document by the Biomim’City Lab report are here analyzed. Table 1 presents context information about these projects. Table S1 presents all the collected data. They have different understandings in terms of sustainability, comprehension, and abstraction of living systems, not evaluated in this study. However, they all derived from a creative approach based on the observation of biological systems.

Table 2 provides an overview of the variables and classification options used in the study, extracted from each project factsheet and compiled on a Microsoft Office Excel 365 database (Microsoft Corporation, Redmond, WA, USA). To make data scientifically sound with our research questions, we merged some of the original classes from the factsheets for the variables “project status”, “project type”, “integration level of the biomimetic approach”, and “biological model”. Document S2 explains each of the classification variables and options.

### 2.2. Analysis

Results were analyzed with a mixed-method approach, using quantitative and qualitative perspectives. At first, we use correlational research, relying on a quantitative typological analysis [17], to explore the sample trends. Following, we used a qualitative narrative and content analysis of text excerpts from the report [17,18]. This step aimed to explore lessons and barriers from the designing team perspective. Both approaches are detailed in the sequence:

#### 2.2.1. Typological Analysis

The typological analysis aims to identify significant trends in the sample, such as their chronological evolution, project types, aimed objectives, level of integration and types of biological models. After data collection, we quantitively analyzed data on Microsoft Office Excel 365 (Microsoft Corporation, Redmond, WA, USA), using frequency and chronological analysis.

#### 2.2.2. Content Analysis

Content and narrative pattern analysis have been done using text excerpts of the original report. Each project factsheet concluded with a section named “Lessons learned” (*Leçons à retenir*), in which designing and editorial teams highlighted main challenges, levers and barriers to the realization of a biomimetic urban project. We extracted these full excerpts from each factsheet and composed a unique textual corpus for analysis.

The textual corpus (Document S2) was then imported to MaxQDA Analytics Pro 2020 (VERBI GmbH, Berlin, Germany). We first used the word cloud function on this software to identify recurrent terms in the corpus. Afterwards, we used the interactive word tree function to explore phrases and narratives around the most frequent word, understand the context, and draw insights from this data.

### 2.3. Methodological Limits

This research is an opportunistic study, and some of its limits reflect the limits of the original data source. Notably, the French report does not document all biomimetic urban projects designed in France, but some of them. Nevertheless, we consider it a representative sample that allows us to infer trends in practice, once it is the most updated information available. Furthermore, this research does not aim to evaluate the pertinence and the environmental performance of the projects and their biomimetic design approach.

## 3. Results

### 3.1. Project Status and Types

**Figure 1:**
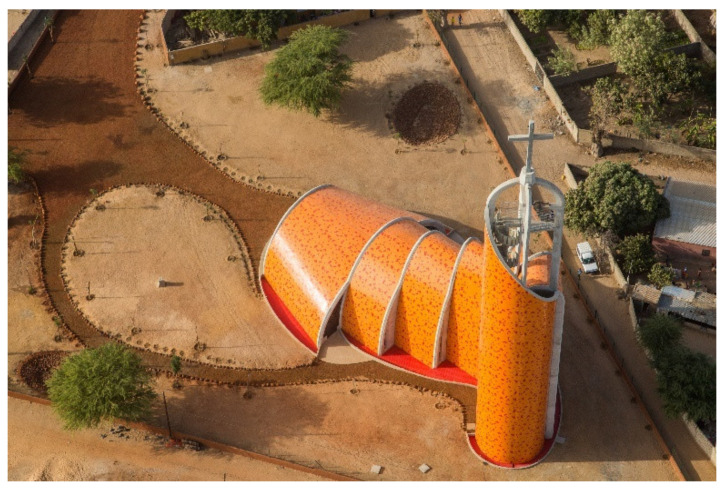
Nianing Church, Sénégal (© Régis L’Hostis/IN SITU Architecture).

We identified that 44% (*n* = 7) of the projects have been accepted by the project owners and are built or under construction. Examples in this category are the Nianing Church, a concluded project located in Senegal (Figure 1). This project finds inspiration in the termite mounds model aiming at passive thermal regulation, a well-described biological model successfully applied in previous projects as the CH2 and the Eastgate Center [5]. Another example is the Quartier de Gally project, a neighborhood under construction that aims at natural ecosystems as models to promote a neighborhood that better integrate urban spaces and nature. 

**Figure 2:**
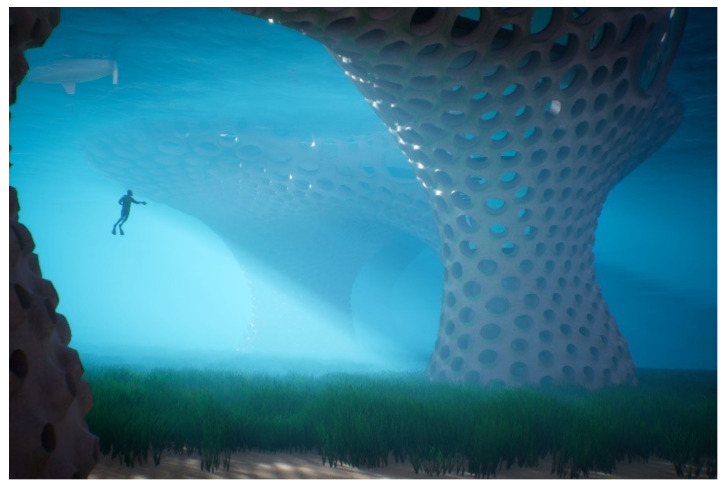
Biolum_Reef (© Treex/Tangram Architectes).

A quarter of the projects (*n* = 4) have been accepted but are not constructed and not under construction. It includes conceptual projects, as the Biolum_Reef, a project that aims to create autonomous human habitat in the sea, presenting bioluminescent bio-inspired technologies and contributing to ocean decontamination and natural habitat provision (Figure 2). This category includes also projects accepted by the client and further abandoned, as the Residence Solaire d’Ordener, a project in which the building orientations were calculated using a solar phyllotaxis algorithm to optimize sunlight exposition, and, finally, projects still in executive design, as the Ecotone project that finds inspiration in transition ecosystems recreating ecosystems services in the urban space.

Furthermore, 31% (*n* = 6) of the projects were not accepted by the project owners. One example is the Osez Josephine project, a mixed-use project that finds inspiration in French forests, aiming for diverse vegetation strategies, more circular water and waste cycles and bioclimatic and evolutive buildings. 

**Figure 3:**
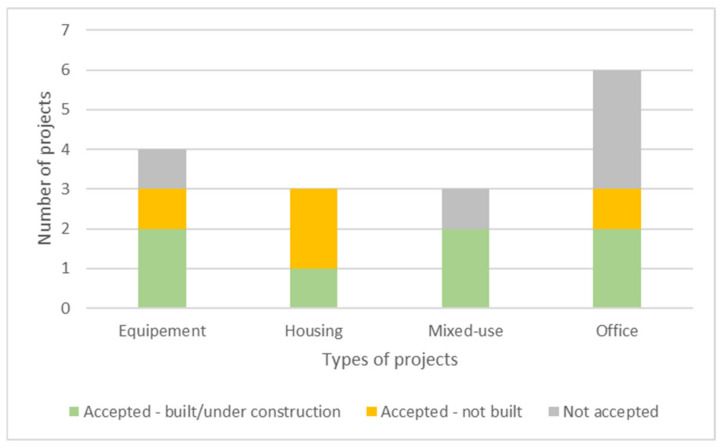
Distribution of projects status according to their types.

**Figure 4:**
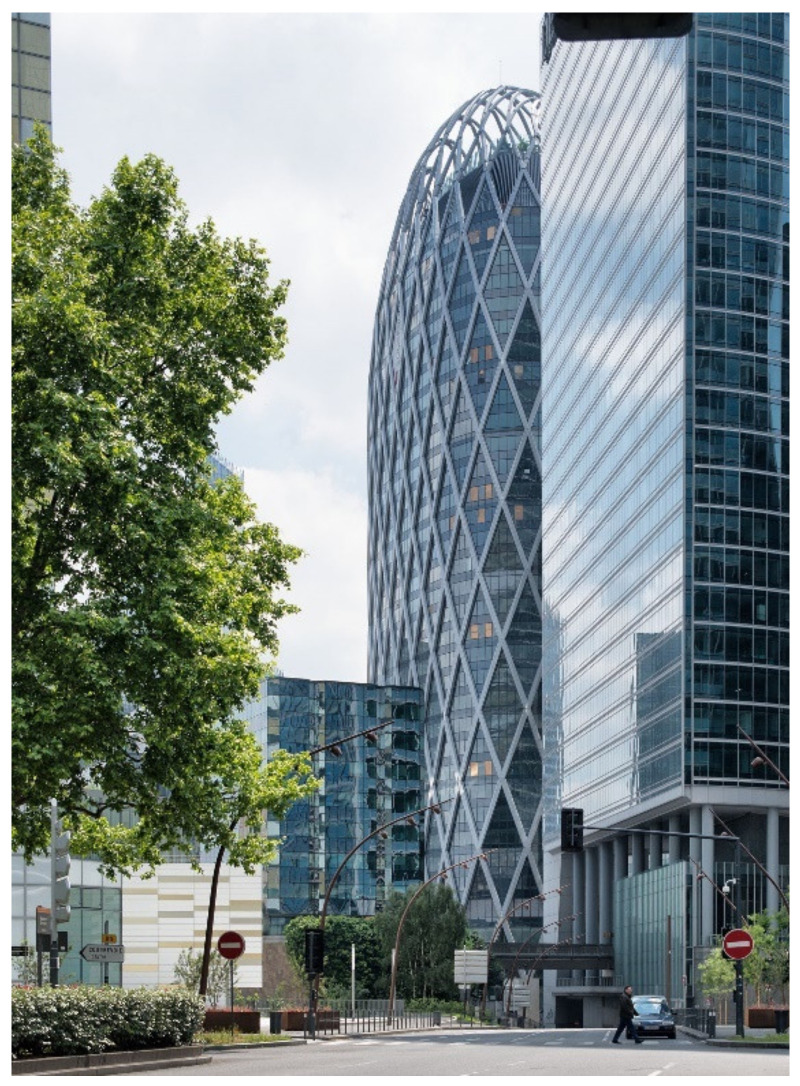
Tour D2, La Défense, Paris (© Pierre Elie de Pibrac/Bechu & Associés).

Office projects are the most represented (37%), followed by equipment projects (25%), as schools and churches (Figure 3). Housing and mixed-use projects are the less represented types (19% each). Office projects mostly implemented biomimetic approaches, but they also represents the project type with more issues to reach implementation phases (Figure 3). One successful office example is the Tour D2 project, which uses the periosteum structure (membrane covering the bones) as model for its exo-structure, which allowed to reduce the total building mass (Figure 4). An example that did not reach the implementation phase is the Parramatta project, which uses a local eel species model for a passive thermal regulation system.

Very few of them addressed the retrofit of existing infrastructures. Only 13% (*n* = 2) integrate some renovation. They are the Osez Josephine and the Quartier de Gally projects. In both, renovations were partial, on some selected pre-existing buildings. 

### 3.2. Chronological Evolution of Projects

**Figure 5:**
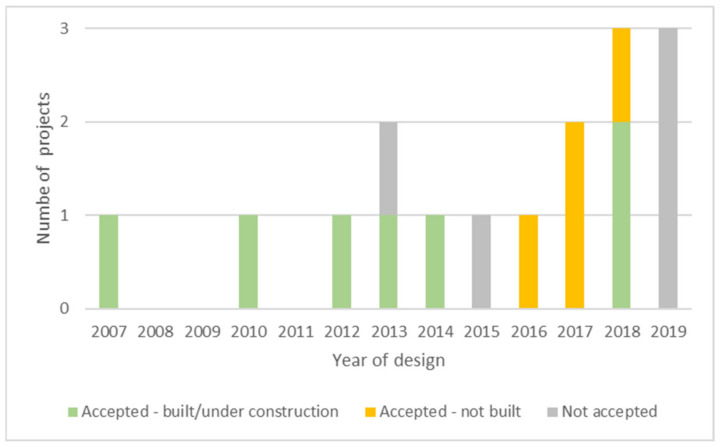
Distribution of the analyzed biomimetic urban projects according to their status and design date.

Figure 5 outlines this sample distribution over time and according to their design year and status. The oldest documented project in the report was designed in 2007. Between 2012 and 2016, the report presents at least one biomimetic project designed per year. Then, the number increases from 2017 to 2019. Nevertheless, since 2013, we also observe an increasing number of projects that did not reach implementation (not built or not accepted).

### 3.3. Objectives

**Figure 6:**
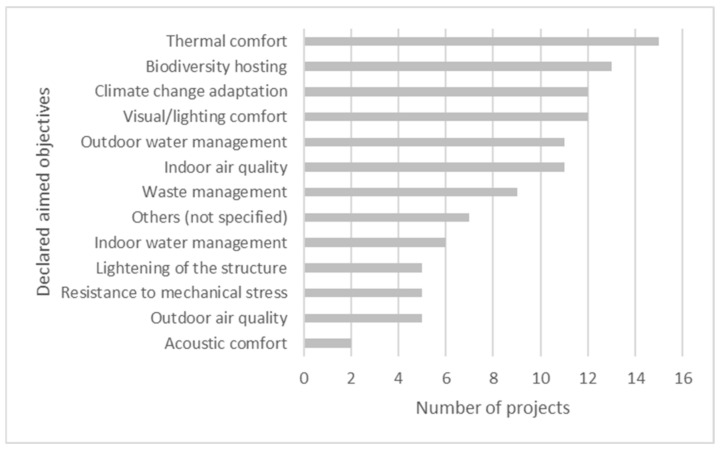
Aimed objectives.

Figure 6 presents the frequency of declared objectives of the projects with their biomimetic approach. We can observe that six objectives were present in more than ten projects (62.5% of the sample): climate change adaptation, biodiversity hosting, outdoor water management, indoor air quality, visual and lighting comfort, and thermal comfort.

**Figure 7:**
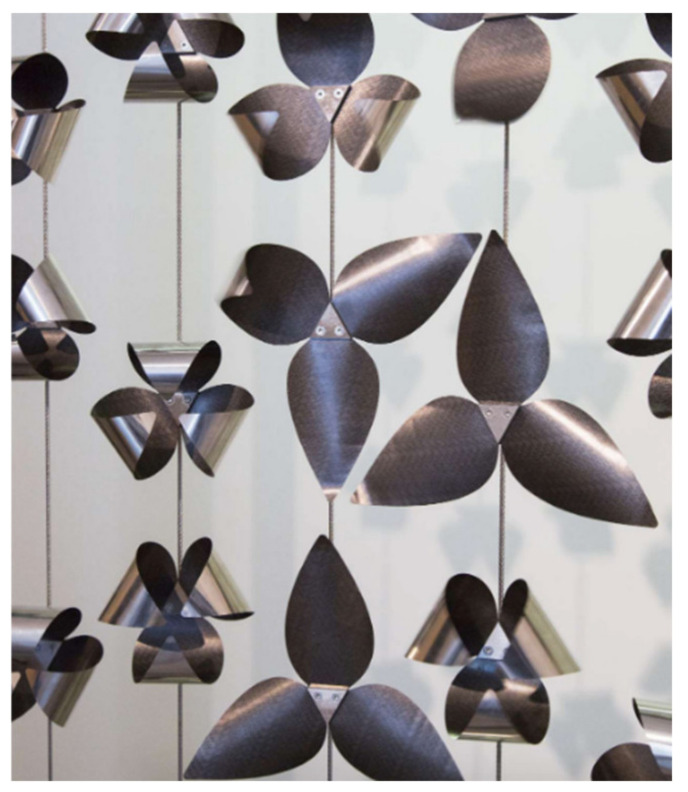
CIRC adaptative solar protections (© Art&Build).

Most of the projects declared to seek several objectives with their biomimetic approach. The average number of objectives aimed per project was 7. The project with a narrower focus is the CIRC, a project that uses bio-inspired adaptative solar protections made of metal with shape memory, aiming for visual and light comfort, and thermal comfort (Figure 7). The projects with a wider focus in the sample, aiming twelve different objectives are the Biolum_Reef, Osez Joséphine and the Pôle d’excellence sur le biomimétisme marin.

**Figure 8:**
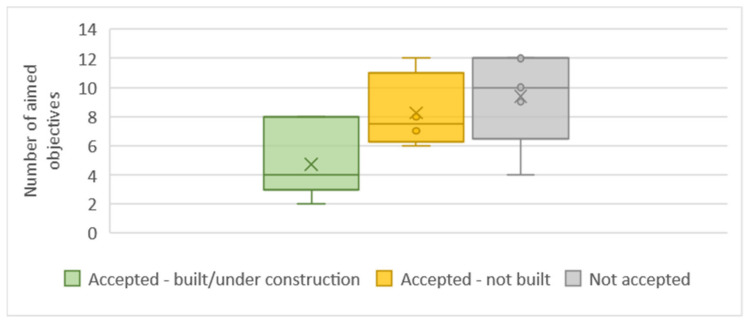
Distribution of the total number of aimed objective in the projects per project status.

We observe that accepted and built or under construction projects in the sample tend to have a narrow focus on their objectives (Figure 8).

### 3.4. Level of Integration

**Figure 9:**
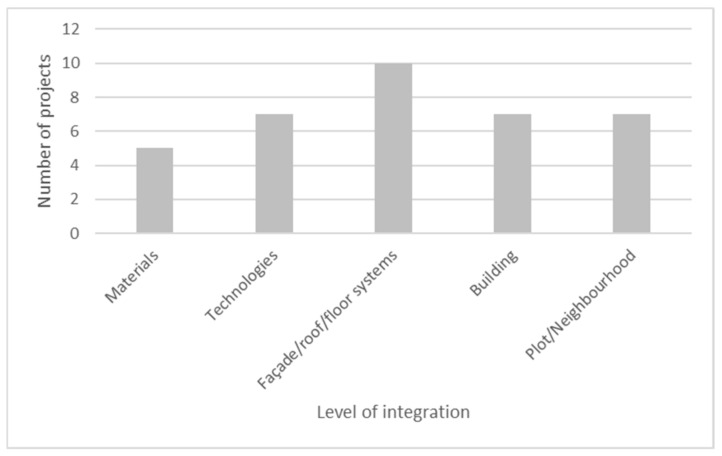
Integration level of the biomimetic approach.

The projects’ most recurrent integration level of the biomimetic approach is at the façade/roof/floor systems (Figure 9). Ten projects applied some level of biomimicry at this level. Examples are the Tour D2 project with its exo-structure, the CIRC with its adaptative solar protections and the Alguesens project, which proposes a bio-façade with algae bioreactors, ensuring building thermal regulation and CO_2_ sequestration.

Moreover, the less explored level is building materials (Figure 9). One example is the Bangkok I’m Fashion Hub, which explored a minimal structural surface in local woven bamboo, inspired by spider webs, associated with a bio-inspired membrane ensuring waterproofing and passive ventilation.

**Figure 10:**
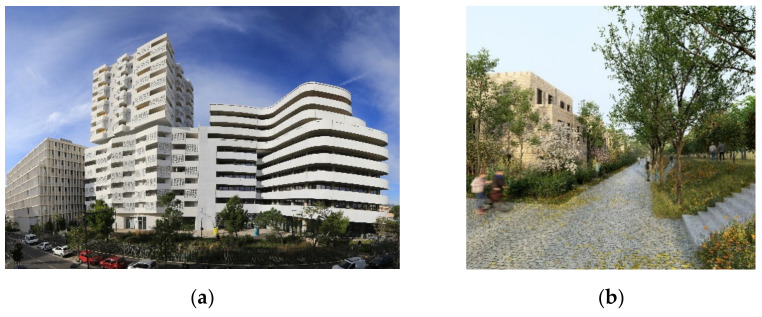
(**a**) Smartseille (© Action Photo Video Thierry Lavernos/Eiffage); (**b**) Quartier de Gally (© ICADE).

At the technology level, one example is the Smartseille project (Figure 10a), which tested a mycelium-based technology to remediate soil pollution on the project site. At the building level, one example is the Estran project, which replicates a foreshore ecosystem in the building, integrating a wetland to serve as a reservoir for rainwater, water treatment system and habitat for biodiversity. Finally, at the plot/neighborhood level, examples are the Quartier de Gally (Figure 10b) and the Ecotone projects.

On average, projects explore two different levels of integration. Three projects explored just one level (CIRC, Nianing Church and Tour D2), and only one project explored all the dimensions (Pôle d’Excellence sur le Biomimétisme Marin).

### 3.5. Type of Biological Models

**Figure 11:**
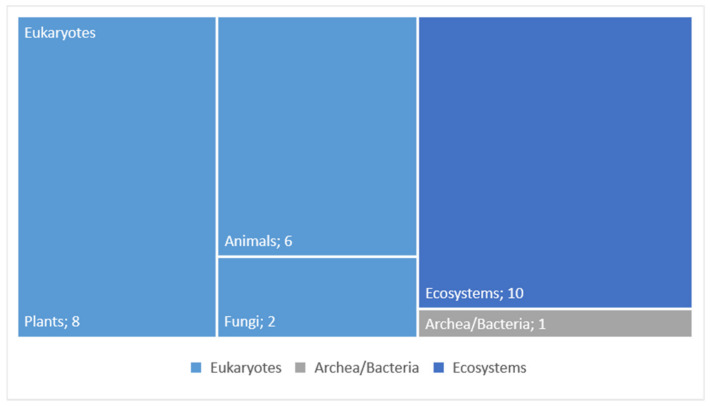
Occurrence of the different types of biological models (*n* = number of observations).

Ecosystems are the most recurrent models, cited in 10 projects, followed by Eukaryotes models as plants and animals. Fungi and Archaea/Bacteria models are the less explored models in our sample (Figure 11). 

### 3.6. Green Labels

**Figure 12:**
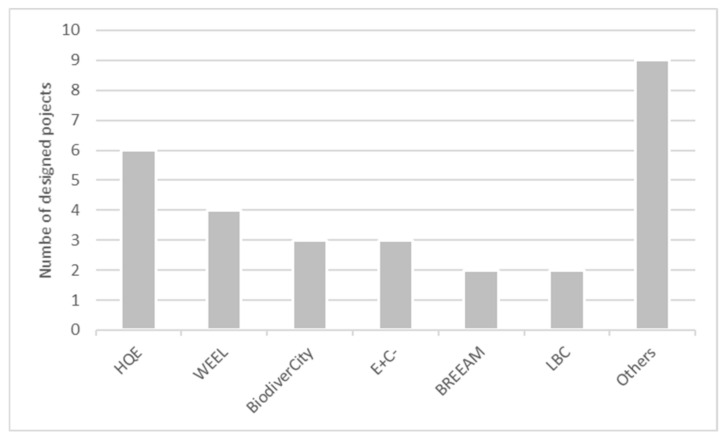
Green building labels.

To give visibility to their sustainable engagements and performances, ten projects from the sample (62.5%) highlighted labels that the projects aimed for or obtained. Fifteen different labels families have been cited, but only six have been cited more than once (Figure 12). We can find a higher presence of standard French market label families as HQE, E+C- and BiodiverCity. Nevertheless, innovative labels, not yet fully adopted by the French market, have been observed, as the LBC.

### 3.7. Design Challenges

**Figure 13:**
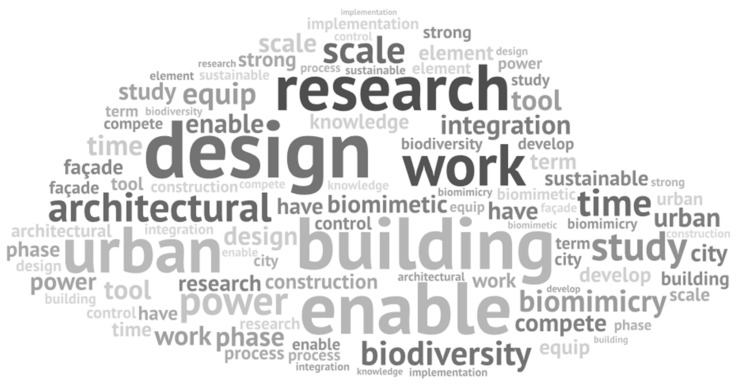
“Lessons learned” section word cloud, using MaxQDA Analytics Pro 2020.

The main recurrent keywords in the “Lessons learned” section from the projects factsheets relates to the design process and its challenges as: “*conception*” (design), “*rechercher*” (research), “*étude*” (study*)*, “*temps*” (time) and “*integration*/*intégrer*” (integration) (Figure 13).

**Figure 14:**
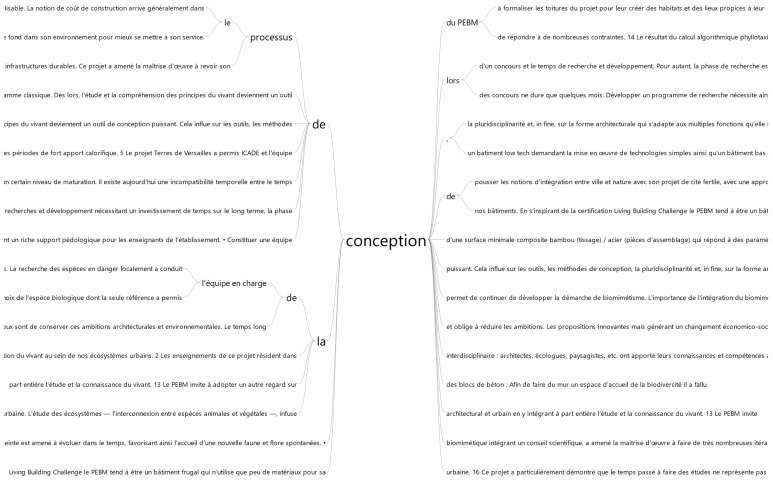
Interactive word tree for “*conception*” using MaxQDA Analytics Pro 2020.

Figure 14 presents the interactive word tree for the word “*conception*” (design), the main identified keyword. The interactive word tree allowed us to identify passages and recurrent ideas presented by the designing teams.

The passages related to the challenges of the design process converge into two main topics: project temporality and team knowledge.

The result highlights that the temporalities of design and research process are distinct, as the available time for the design of urban projects, in the French concurrently context, is short. However, the research and development process usually necessary to apply biomimicry into the process is long. Nevertheless, designers acknowledge the importance of bridging the gap between the two practices. The following passages illustrate this:

1. *“research and development process at the heart of biomimetic solutions: the bio-inspired facades of the project were the subject of a research project, with several prototypes and tests before being integrated into the project.”*
2. *“The long design time allows the biomimicry approach to continue to be developed.”*
3. *“Today there is a temporal incompatibility between the design time in a competition and the research and development time.”*
4. *“This project has particularly demonstrated that the time spent on studies does not represent a cost, but a real investment (…).”*
5. *“The limitations of this project are the number of associated research projects that would need to be launched over the long term.”*

The second challenge is related to the team experience and knowledge of biomimicry, biology and ecology. Designers acknowledge the importance of working in a multi-disciplinary context, integrating experts, and building biomimicry capacity on the design team and other project stakeholders. The following passages support this:

1. *“The importance of integrating biomimicry right from the competition phase with an acculturation of the architects and the project owner upstream of the project is a key element.”*
2. *“Set up an interdisciplinary design team: architects, ecologists, landscape designers, etc. contributed with their knowledge and skills to build a project (…).”*
3. *“This project provided an opportunity to explain the biomimetic approach and its potential to the contracting authority. The latter understood the real opportunities that the biomimetic tool offers (…) but we also identified some inherent obstacles related to their poor knowledge on the subject.”*
4. *“This project led the project management to review its architectural and urban design process by integrating the study and knowledge of living things into it.”*
5. *“The process of biomimetic design integrating a scientific council, led the project management to do many iterations and to develop their knowledge on many subjects. In return, the knowledge not directly used has enriched the process allowing the project management to improve its practice.”*

## 4. Discussions

### 4.1. An Increasing Trend over Time

In the analyzed sample, we observed an increasing trend over time of projects applying biomimicry for sustainable development in France. This trend deserves further exploration with a larger and exhaustive sample. This trend converges with the increase in biomimicry research applied to architecture and urban design in the last years already demonstrated by previous research [4,5]. Two important milestones in the French timeline that contributed to more visibility and interest in biomimicry are the Alguesens project (2016) and the Ecotone project (2017). Alguesens is one of the winners of the “*Reinventer Paris*” competition, aiming to innovate and experiment with new approaches to bring selected Parisian sites to life. Ecotone is one of the winners of the “*Inventons la Métropole du Grand Paris*” competition, one of Europe largest contemporary competition, promoting innovative projects to rethink critical sites in the Parisian metropole region.

Nevertheless, our sample also presents a growing number of projects that do not reach the implementation phase. Several factors could be related to these projects non-acceptance, not related to the biomimetic approach, as the highly competitive context of urban projects competitions, the quality of the overall project, the project costs, and political preferences. However, the role of the biomimetic approach in the project acceptance by the final client deserves further exploration. Hayes et al. (2019) highlighted in their case studies that the absence of well documented and successful application examples remains a significant barrier in the field. Designers from the analyzed projects also indicated that the project owner’s lack of understanding of biomimicry could be a barrier to the project success. 

### 4.2. A Focus on Macroscopic Models

Most of the analyzed biological models remained at macroscopic scales such as ecosystems and eukaryotes. This finding converges with previous results highlighting a taxonomic bias in selecting biological models [14]. This trend can be explained due to the lack of academic knowledge of biological models by the design team and the lack of time to analyze, compare, and then select relevant living models to face the project challenges. Integrating stakeholders with a strong background in biology or ecology in the design teams can be a way to explore further biological models [19,20], mainly in a short delay design context. Another lever is developing tools and methods to help designers navigate biological knowledge easily, as being explored by fellow researchers as reviewed by Wanieck et al. [21].

Ecosystem-level biomimicry is a growing research topic [4,16], and its first position in our sample highlights a particular interest in the practice for this subject. We assume that ecosystem-level inspiration helps address transversal and systemic urban challenges such as biodiversity loss, the materials, and energy flows between urban and ecological systems [16,22,23].

### 4.3. Organising the Design Process

There is a significant challenge in integrating research and development with urban biomimetic practice [24]. Designing teams highlighted the different temporalities and the positive aspects of integrating research and specialists in the projects. Integrated design process (IDP) could be a helpful design framework to organize biomimetic architectural and urban projects design. It relies on multi-disciplinary teams early engaged in the project, with a straightforward decision-making process and an external facilitator, aiming to foster sustainable and regenerative development on urban projects keeping in mind the project quality and costs [25].

Another leverage point would be to work alongside project owners to raise awareness of the topic and integrate biomimetic specifications on their project briefing and technical specifications.

### 4.4. Thermal Comfort and Biodiversity Hosting: Two Major Entry Points

Thermal regulation is a contemporary urban challenge well-explored in biomimetic architecture and illustrated by numerous well-documented proofs of concept [2,5]. Successful biomimetic cases reduce the risks of rejection for project stakeholders [16]. This could explain the number of observations of this objective within the sample (*n* = 15). Among the analyzed projects, diverse solutions and strategies have been observed to reach this objective through biomimicry. The Skolkovo Innovation Center proposes a site organization that minimizes thermal loss, imitating penguins comportment to save and share heat. Other projects deal with thermal regulation through the façades, as the CIRC and Alguesens, and others with passive ventilation systems as the Nianing Church and Parramatta projects. 

**Figure 15:**
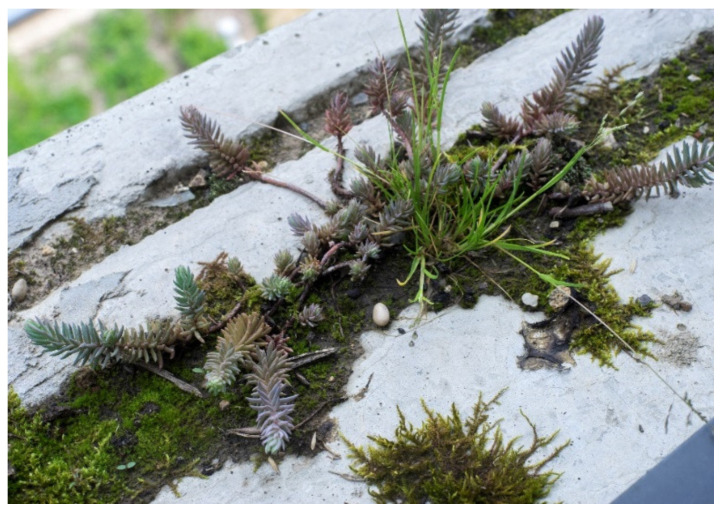
Biodiversity at the walls of Ecole des Sciences et de la Biodiversité (© Myr Muratet/ChartierDalix).

Regarding biodiversity, several projects declared to contribute to creating habitat for biodiversity (*n* = 13). However, only one project, the Ecole des Sciences et de la Biodiversité, clearly stated the biomimetic approach to reach this objective. To bring biodiversity back to the urban space and promote human and other species co-habitation, the building walls find inspiration in cliffs walls, using concrete blocks that create holes and niches for different animal and plant species (Figure 15).

Even if biodiversity and ecosystems models have a high interest in our sample and practice, very few bio-inspired projects achieve to address their complexity adequately. For example, projects finding inspiration on ecosystems tend to look mainly to materials and energy flows, translating it to into urban metabolism and circular economy solutions. However, the ecosystems biotic and abiotic structure and ecosystems functions are much less explored [22,26], leading to simplistic and metaphorical ecosystem-level biomimicry application.

### 4.5. Biomimetics Applied to Retrofit

The renovation of urban infrastructures remains the main lever over the coming years to reduce human impacts on biodiversity and ecosystems [27]. In 2019, retrofitting activities represented 54.4% of the building market in France [28]. For 2020, this activity is expected to increase +0.9% [29].

Biomimicry offers an opportunity to handle retrofitting with a sustainable development perspective [14,23]. Nevertheless, only a few of the analyzed projects had partially retrofitted existing infrastructures. This topic deserves further exploration. The reasons could be related to a lack of research and practice of biomimicry for architectural and urban retrofitting but also, retrofitting projects and stakeholders could be less open to innovative approaches. 

## 5. Conclusions

Biomimicry seems to be a design approach with growing interest in the French market, but several unresolved questions and challenges remain. 

The analyzed data highlighted the gaps between biomimetic and biological research teams and urban designers practitioners. To facilitate the knowledge transfer from biological and ecological disciplines to architecture and urban design practice is a major challenge. Our data demonstrated the importance of rethink design team composition and projects design phase organization to bridge this gap.

The case studies also highlighted the variety of sustainability goals addressed through biomimicry in France. Nevertheless, it would be worth formally exploring the different urban stakeholders’ motivations applying biomimicry and the difference in design process and outputs between applying it with a narrow or a broad focus.

Furthermore, it would also be essential to study and evaluate the performance of these projects concerning their objectives. Projects tend to use labels to show their performances, but urban biomimicry still lacks specific sustainable performance metrics. The use of life cycle assessments and ecosystems services assessments could allow a proxy to these performance assessments.

Finally, to document and analyze the practice of biomimicry on architectural and urban projects allowed us to have some perspective from the French practice. To showcase national projects can tackle the incomprehension of biomimicry by urban stakeholders and project owners and raise awareness of the topic. Nevertheless, such benchmarks are rare and deserve to be enlarged. A more exhaustive study would probably highlight new challenges, and it remains relevant work to be done. 

## Figures and Tables

**Table 1:** Analyzed projects.

Project Name	Project Location	Design Year
Alguesens	Paris, France	2016
Bangkok I’m Fashion Hub	Bangkok, Thailand	2015
Biolum_Reef	Marseille, France	2017
CIRC Lyon	Lyon, France	2018
Quartier de Gally (Cité Fertile—Terres de Versailles)	Versailles, France	2018
Ecoquartier Smartseille	Marseille, France	2013
Ecotone	Arcueil, France	2017
Eglise de Nianing	Nianing, Senegal	2014
Estran	Biarritz, France	2019
Groupe Scolaire des Sciences et de la biodiversité	Boulogne-Billancourt, France	2010
Osez Joséphine	Rueil Malmaison, France	2019
Parramata	Parramata, Australia	2013
Pôle d’Excellence sur le Biomimétisme Marin	Biarritz, France	2019
Residence solaire d’Ordener	Senlis, France	2018
Skolkovo Innovation Center	Skolkovo, Russia	2012
Tour D2	Paris, France	2007

**Table 2:** Collected data from the original factsheets with the classification options used in the study.

Variables	Classification Options
Year of design	-
Project Status	Accepted and constructed or under construction|Accepted but not constructed|Not Accepted.
Type of project	Housing|Equipment|Office|Mixed-use.
Renovation	Partial renovation on the project|No renovation
Aimed objectives with the biomimetic approach	Thermal comfort|Visual/lighting comfort|Acoustic comfort|Indoor air quality|Outdoor air quality|Resistance to mechanical stress|Indoor water management|Outdoor water management|Biodiversity hosting|Adaptation to climate change|Lightening of the structure|Waste management|Others
Integration level of the biomimetic approach	Materials|Technology|Façade/roof/floor system|Building|Plot/Neighborhood.
Type of biological model	Eukaryote—Animal|Eukaryote—Plant|Eukaryote—Fungi|Archaea/Bacteria|Ecosystem
Aimed labels and certifications families	Batiment passif|BDM|BiodiverCity|BREEAM|Cradle to Cradle|E+C-|Effinergie|HQE|LBC|LEED|Matériaux biosourcés|Nature-Art-Education|NF Habitat|RT2012 |WELL
Lessons learned and challenges	Text excerpts (Document S3)

## Data Availability

The data presented in this study are available in the Supplementary Materials.

## Supplementary Materials

The following are available online at https://www.mdpi.com/article/10.3390/biomimetics6020027/s1, Table S1: Projects data, Document S1: *Projets urbains bio-inspirés: un état des lieux des projets français*, Document S2: Variables and classification options definitions, Document S3: Leçons learned text excerpts.
