# Supplementary material for: Biomimicry in French Urban Projects: Trends and Perspectives from the Practice
Source: Biomimetics (Basel). 2021 Apr 27;6(2):27. doi: 10.3390/biomimetics6020027 (PMC8167731; doi:10.3390/biomimetics6020027)

PROJETS URBAINS BIO-INSPIRÉS

Un état des lieux des projets français

Édition 2020

Biomim' CITY LAB

Ceebios

Un document Ceebios & Biomim'City Lab

Edition : Ceebios
Eduardo Blanco
Chloé Lequette
Caroline Robert

Collaboration :
Art&Build
Bechu & Associés *- membre confondateur du Biomim'City Lab*
Ceebios *- membre confondateur du Biomim'City Lab*
ChartierDalix
Eiffage *- membre confondateur du Biomim'City Lab*
Elan *- membre confondateur du Biomim'City Lab*
Icade *- membre confondateur du Biomim'City Lab*
In Situ Architecture *- membre confondateur du Biomim'City Lab*
NewCorp Conseil *- membre confondateur du Biomim'City Lab*
Nobatek/INEF4 *- membre confondateur du Biomim'City Lab*
Renault *- membre confondateur du Biomim'City Lab*
Tangram Architectes *- membre confondateur du Biomim'City Lab*
XTU Architects

INTRODUCTION

Ce document est issu d'un travail collaboratif mené par le Biomim'City Lab, groupe de travail sur la ville bio-inspirée animé par Ceebios.

Le Biomim'City Lab souhaite fédérer les acteurs conscients du rôle du vivant pour la ville de demain, afin de monter en compétence, co-développer de nouveaux outils, faire évoluer les pratiques, partager les expertises, créer une zone de rencontres et d'expérimentation autour du sujet du biomimétisme et du design régénératif.

Ce document marque la première année d'activité de ce collectif lancé en octobre 2019, et répond aux objectifs suivants :

1. Mettre en valeur les projets urbains et architecturaux français bio-inspirés ;

2. Recueillir des données, résultats et retours d'expériences issus de la mise en œuvre d'approches bio-inspirées en architecture et urbanisme, puis les organiser sous un format didactique et pratique ;

3. Proposer un outil de communication et de sensibilisation, à même d'aider les acteurs de la « fabrique de la ville » à comprendre les opportunités qu'offre le biomimétisme pour la transition écologique.

17 projets urbains bio-inspirés, à l'échelle du quartier et du bâtiment, sont décrits ci-après dans des fiches standardisées, selon leurs différents niveaux de maturité. Aux **16 projets réalisés en France** ou par des acteurs français, nous avons souhaité ajouter **un projet international** particulièrement emblématique en la matière, qu'est le Bullit Center à Seattle, aux États-Unis, et dont la fiche ouvre ce premier recueil.

Dans les 17 projets détaillés dans ce cahier certains n'ont pas été lauréats, donc jamais réalisés. Néanmoins, une démarche approfondie a été portée par les équipes opérationnelles dans chacun de ces projets.

L'HABITAT D'HOMO SAPIENS EN QUESTION

Nous ne sommes pas prêts, mais nous pouvons changer

Notre habitat du XXIe siècle, au sens écologique du terme, et particulièrement nos écosystèmes urbains qui concentrent déjà plus de la moitié de la population mondiale, ne sont ni prêts, ni adaptés à nos nouvelles conditions de vie climatiques, écologiques, énergétiques, sanitaires et sociales. Cet habitat a été pensé et conçu à une autre époque, une autre ère, un autre temps, qui ne connaissait, ni n'envisageait les deux facteurs vitaux et structurants qui redessinent et interrogent l'avenir de l'humanité elle-même : le dérèglement climatique et la perte massive de biodiversité.
Si nous changeons d'époque, nous devons aussi changer notre façon d'habiter. Aujourd'hui, les écosystèmes urbains se densifient et se propagent par l'artificialisation et l'hermétisation des sols. Ils hébergeront 65% de la population mondiale en 2050, à moins de 150 km des côtes pour la plupart, et sont déjà les principaux émetteurs (2/3) des gaz à effet de serre.

Par les facteurs d'impacts qu'elles représentent, les villes à la fois souffrent et entretiennent encore les maux climatiques et écologiques. Pourtant, face à l'impérieuse nécessité de transition et d'adaptation, les villes et leurs concepteurs changent, évoluent, intègrent de nouveaux critères, développent des approches plus holistiques, cherchent des solutions et innovent... pour faire du développement urbain un contributeur positif au service d'une approche régénérative et de la capacité de résilience de l'espèce humaine toute entière.
Si nous voulons vivre mieux, nous devons repenser radicalement la façon de concevoir et développer nos écosystèmes pour les reconnecter à la nature, à la vie elle-même : notre habitat doit redevenir un espace propice à la vie, et c'est possible, grâce aux leçons du vivant.

Cette dynamique positive de réinvention des villes est en cours, il faut l'encourager et l'amplifier. C'est tout l'enjeu du Biomim'City Lab, créé à l'initiative d'ICADE et rejoint par BECHU & ASSOCIÉS, ELAN, CEEBIOS, NOBATEK/INEF4, IN SITU ARCHITECTURE, EIFFAGE, RENAULT, TANGRAM ARCHITECTES et NEW CORP CONSEIL. Le Biomim'City Lab s'intègre dans cette dynamique et souhaite y contribuer en proposant le vivant comme modèle (le biomimétisme) ; pour habiter nos villes comme on habiterait la nature.

LA NATURE COMME MODÈLE, UN PUISSANT ET SURPRENANT LEVIER DE RECONFIGURATION DES VILLES

La nature peut nous aider

Réintégrer de la nature en ville est une formidable dynamique à laquelle le Biomim'City Lab s'associe, mais c'est une condition nécessaire et non suffisante, il faut aussi repenser la ville par la nature, à l'instar du mode de fonctionnement d'un écosystème. La nature en effet n'est plus un décor ou un outil de valorisation urbaine, c'est aussi et surtout une alliée, une source de solutions et un modèle.
En réalité, repenser la ville avec et par la nature est la condition inévitable de son renouveau.
Le biomimétisme, déjà avéré dans de très nombreux domaines industriels, mais également dans l'architecture par des exemples remarquables, se propose comme une méthode d'analyse et de conception au service de la résilience de notre habitat d'espèce.

Le Biomim'City Lab propose de poser cette approche et cette philosophie au cœur de sa réflexion et de son action, pour dupliquer les expériences concluantes déjà mises en place, en proposer de nouvelles, et surtout changer d'échelle pour penser l'ensemble de la ville par une approche bio-inspirée, holistique et résiliente.
Aller plus loin : à l'instar des enjeux écologiques, la question n'est plus seulement d'être « neutre » en termes d'impacts mais de mettre en place des approches « régénératives ». La ville ne doit plus seulement compenser ou s'adapter, mais réparer pour recréer les conditions de la vie, et améliorer les conditions de vie de ses habitants.

LA VILLE BIO-INSPIRÉE COMME UNE RÉPONSE RÉALISTE ET INNOVANTE AUX GRANDS ENJEUX

Des solutions concrètes

L'approche préconisée par Le Biomim'City Lab permettra d'atteindre 3 grands objectifs :

1. Créer des bénéfices écosystémiques :
Dans le monde vivant, la coopération, par les gains d'efficacité qu'elle permet, est à l'origine de toutes les grandes transitions de l'histoire de la vie. S'inspirer de la nature permettra de réduire la consommation énergétique des bâtiments et des villes (alternative à la climatisation par de l'auto-ventilation passive ; production délocalisée et partagée ; façades réactives et bioclimatiques…) ; de proposer une sobriété constructive (moins de matière, sourcée localement, bio-sourcée, éco-conçue…) ; de participer au rafraîchissement des villes et à la lutte contre les îlots de chaleur (végétalisation) ; de lutter contre les risques d'inondations (réintroduction du cycle de l'eau en boucles locales) ; de lutter contre la pollution atmosphérique (captation et séquestration CO_2, dépollution végétale…) ; de recréer des villes nourricières par des espaces de production en circuits courts (permaculture, fermes urbaines…) ; de repenser la géométrie urbaine pour optimiser les flux, la mobilité et réintégrer un réseau parallèle de vaisseaux de nature…

2. Générer des impacts politiques, sociaux et culturels :
La ville bio-inspirée permet de développer la pédagogie de l'évidence et du bon sens auprès des habitants en forte demande de réintroduction de nature en ville, et préconscients du génie du vivant dans tous les domaines. Proposer la nature comme modèle fait consensus, mais surtout fait du bien et réenchante une vision d'avenir rassurante et non exclusivement techno-centrée. Réintroduire la nature et ses bénéfices écosystémiques permet de reconnecter les habitants entre eux, et avec la nature. De ce point de vue, en rapprochant les habitants et la nature, la ville bio-inspirée contribuera de façon essentielle à une mission d'intérêt public planétaire.3. Contribuer à l'attractivité et à la santé des territoires :
En recréant des espaces fertiles, nourriciers et producteurs. Le vivant s'approvisionne en circuits courts. Penser la ville bio-inspirée, c'est recréer de l'économie à la fois circulaire et rapprochée. Ceci tant du point de vue de l'approvisionnement alimentaire (fermes urbaines et agriculture de proximité) que des filières économiques. Un biotope crée et propose les conditions favorables au développement de sa biocénose (ses habitants). Comme un biotope urbain, la ville bio-inspirée favorise les échanges, les approches symbiotiques et bénéfiques entre ses acteurs, et encourage la croissance organique par le rapprochement des flux d'échanges de proximité. En autre conséquence, ce raccourcissement des filières d'échanges permettra de réduire la pression sur la planète à plus grande échelle. Ainsi, nous pouvons concrètement faire de la ville une ressource pour répondre aux grands enjeux de demain.

12 ACTIONS ET ENGAGEMENTS POUR ALLER PLUS LOIN

La bonne nouvelle c'est que nous avons des solutions, et nous ne sommes qu'au début de leur mise en œuvre. L'effet de démultiplication et de levier est devant nous. Nous avons tout à y gagner.

1. Le Biomim'City Lab propose de mettre en réseau les acteurs de la « fabrique de la ville » et de créer de l'intelligence collective. À l'instar des écosystèmes naturels, la résilience vient souvent de la diversité des espèces et organismes, qui s'apportent des bénéfices mutuels et contribuent à l'équilibre permanent. Le Biomim'City Lab se propose donc en diversité sociale et culturelle, académique et technique, car nous croyons à la force de la créativité collégiale pour déformer les schémas de pensée et sortir des silos.

2. Le Biomim'City Lab sera un laboratoire d'analyse, de partage d'informations et de prospectives. Le collectif analysera les impacts et les enjeux de la non mise en conformité des villes avec les enjeux de résilience (sanctions juridiques, risques sanitaires, risque politique...), et recensera l'ensemble des cas concrets et des travaux scientifiques nécessaires au développement de la ville bio-inspirée de demain. Ceci permettra de nourrir l'action, d'accélérer des projets, d'en faire naître de nouveaux, d'éclairer l'action publique et d'accompagner les décideurs. Le Biomim'City Lab concourra à lancer des appels à projets, produira de la littérature scientifique et partagera des cas concrets.

Un collectif engagé et force de propositions, comme :

3. Proposer un nouveau cahier des charges pour la conception de l'espace urbain, avec de nouveaux indicateurs de conception appliquant « les principes du vivant » comme modèle et référence. Ceci pour réduire, voire inverser, l'impact environnemental global du secteur immobilier (usages des ressources, pollutions, appauvrissement des sols, érosion de la biodiversité) qui pourrait être mesuré par un bilan annuel sectoriel.

4. Placer la conception urbaine bio-inspirée au service de la sobriété énergétique, notamment dans l'objectif de réduire l'empreinte carbone des systèmes de climatisation énergivores. La climatisation est en effet la bombe énergétique de demain, or la nature regorge de solutions pour nous aider à rafraîchir par la revégétalisation/renaturation ou à ventiler de façon passive des bâtiments par des techniques bio-inspirées déjà avérées. Les technologies inspirées du vivant peuvent aussi nous aider à mieux nous isoler, à créer de la thermorégulation passive, à hybrider les systèmes de production, à mieux profiter du solaire...

5. Repenser le cycle de l'eau en milieu urbain à l'instar de celui des écosystèmes naturels à travers des approches de « villes éponges » pour mieux s'adapter aux canicules, aux sécheresses et aux inondations sur le long terme.

6. S'engager à créer des espaces urbains à biodiversité positive et régénérative en démultipliant leur « production fertile » par des aménagements bio-inspirés : ne pas seulement planter des arbres, mais des espaces mixtes propices à la recréation de biodiversité.

7. Faire baisser la température des villes (rafraîchissement, lutte contre les îlots de chaleur, réduction de la consommation énergétique).

8. Contribuer à réduire la pollution atmosphérique par la séquestration de carbone et la dépollution/filtration bio-inspirée et bio-assistée.

9. À l'instar des organismes vivants qui s'approvisionnent en ressources disponibles au sein de leur biotope, **définir une charte d'approvisionnement de proximité** pour réduire le rayon d'origine des matériaux nécessaires aux projets et augmenter la part des matériaux locaux.

10. Constituer des équipes de projet pluridisciplinaires et multiculturelles, en intégrant obligatoirement et systématiquement des écologues et scientifiques dans les équipes de projet.

11. Proposer des programmes de formation et de sensibilisation à la conception bio-inspirée auprès des équipes.

12. Créer un rendez-vous annuel de présentation du bilan des actions et engagements et éditer un rapport annuel.

LE GRAND DÉFI

Notre capacité à réinventer des espaces de vie propices à la vie dépendra de notre capacité à penser, créer et agir ensemble, à mêler nos savoirs et passions, à nous écouter, à animer cette confluence des talents, finalement à stimuler notre intelligence collective au service de notre communauté de destins. Participez ! Rejoignez-nous !

Biomim' CITY LAB

ICADE · bechu + associés · Ceebios · EIFFAGE · ELAN · IN SITU ARCHITECTURE · NewCorp Conseil · Nobatek INEF4 · RENAULT · TANGRAM ARCHITECTES

1 projet internationnal

+

16 projets français

LISTE DES PROJETS

01. THE BULLITT CENTER 10
Un bâtiment autonome & biophile

02. ALGUESENS 18
Une symbiose entre algues et bâtiment

03. BANGKOK, I'M FASHION HUB 24
Une surface minimale en bambou, une peau multifonctionnelle adaptative

04. BIOLUM_REEF 30
Un récif habité éco-vertueux

05. CIRC LYON 36
Une façade adaptative bio-inspiré

06. CITÉ FERTILE – TERRES DE VERSAILLES 42
Quartier du Parc

07. ÉCOQUARTIER SMARTSEILLE 48
Démonstrateur de la ville durable méditerranéenne

08. ÉCOTONE 54
Un écosystème complet en pleine ville

09. ÉGLISE DE NIANING 60
Regulation thérmique passive inspiré des termitières

10. ESTRAN 66
Un écosystème complet inspiré du monde marin

11. GROUPE SCOLAIRE DES SCIENCES & DE LA BIODIVERSITÉ 72
Inviter la biodiversité au bâtiment

12. OSEZ JOSÉPHINE 80
Faire pousser les bâtiments

13. PARRAMATA 86
Respirer comme une anguille, briller comme une méduse

14. PÔLE D'EXCELLENCE SUR LE BIOMIMÉTISME MARIN 92
Une hybradation entre architecture & paysage

15. RÉSIDENCE SOLAIRE D'ORDONER 100
Optimiser la consommation d'énergie avec la phyllotaxie

16. SKOLKOVO INNOVATION CENTER 106
S'inspirer de l'organisation sociale des manchots

17. TOUR D2 112
Une exostructure pour économiser de la matière

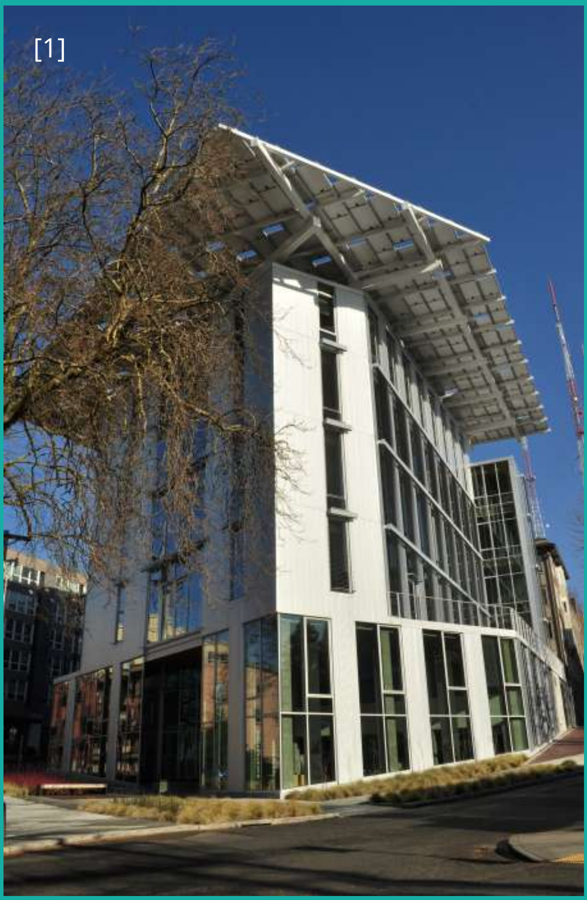
[1]

THE BULLITT CENTER

Adresse : 1501 East Madison Street Seattle, WA 98122 États-Unis
Statut du projet : construit
Date de livraison : 2013
Type/usage : tertiaire, immeuble de bureaux
Rénovation : non
Climat : CSB - climat tempéré chaud avec été sec
Surface du projet : 4 645 m²
Coût de construction : 18 500 000 $ (2013)
Coût final du projet : 32 500 000 $ (2013)

RÉSUMÉ

Situé à Seattle dans l'état de Washington, le Bullitt Center est considéré à ce jour comme étant l'un des bâtiments commerciaux les plus écologiques au monde. En 2013, le Bullitt Center devient le troisième bâtiment à obtenir la certification Living Building Challenge.
En s'inspirant du Pin de Douglas, espèce endémique de la région, le Bullitt Center tente de s'insérer au sein de l'écosystème urbain de Seattle sans le perturber ni y émettre des externalités négatives.
Autonome en énergie et en eau, le Bullitt Center a été conçu dans l'objectif de démontrer qu'il était possible d'ériger un bâtiment commercial ayant une empreinte écologique neutre, et par ailleurs non dépendant des énergies fossiles.
La démarche intérieure biophile permet aux utilisateurs de renouer avec leur environnement et d'augmenter ainsi leur sentiment de bien-être.

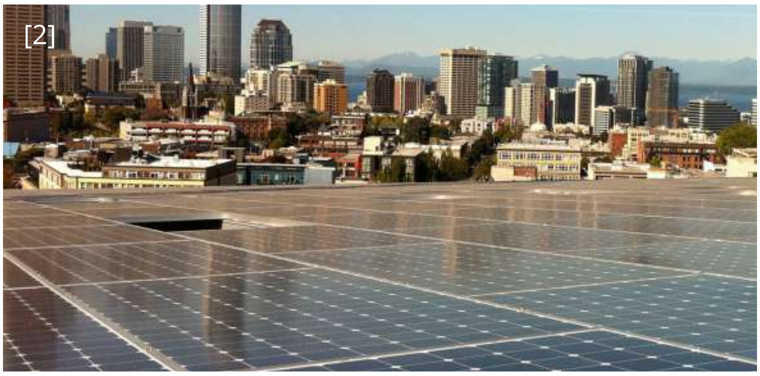
[2]

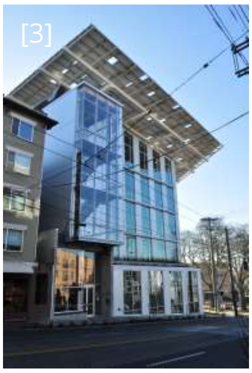
[3]

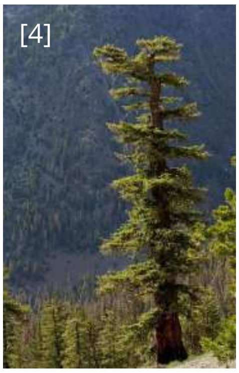
[4]

Revenir à la liste des projets

Rôle des principaux participants

Commanditaire : Bullit Foundation
Architectes : Miller Hull
Architecte paysagiste : Berger partenariat
Entrepreneur général, Core & shell : Schuchart
Ingénierie MEP : PAE
Ingénieur structurel : DCI Engineers
Ingénierie Mécanique : PSF Mechanical
Ingénierie et conception photovoltaïque : Solar Design Associates
Systèmes d'eau : 2020 Engineering
Illumination : Luma Lighting Design
Construction & installation de panneaux solaires : Northwest Wind and Solar
Enveloppe du bâtiment & tests de performance : RDH
Contrat général, amélioration locataire : Foushee & Geotility
Partenaire de développement : Pont 32
Mise en service : Keithly Barber Associates
Études d'impacts : Bullitt Center Assessment Team (études d'impacts environnementales, économiques, évaluation des services écosystémiques rendus, et éventuelle réplicabilité)

Coûts

Coût de construction : 18 500 000 $ (2013)
Coût final du projet : 32 500 000 $ (2013)
Coût foncier : 3 380 000 $ (2010)
Prix de location des bureaux au m² : entre 28 et 30 $/m² (2013)
Tarif assurances taxe & frais de maintenance : 116, 25 $/m²
Autres : Afin d'être autonome en énergie et en eau, chaque locataire de ce bâtiment dispose d'un crédit annuel d'eau et d'énergie auquel il doit se tenir.

Principaux prix

2016 • Energy in Design, AIA Seattle
2015 • Beyond Green Award First Place Sustainable Buildings Industry Council
• TOP Ten, AIA committee on environment (COTE)
2013 • Sustainable Building of the Year World Architecture News
• A+ Award Architecture & Sustainability Special Mention Architizer
• Building of the Year American Buildings Company
• Sustainable Building of the Year Metal Architecture Magazine
• Best Green Project of the Year ENR National
2012 • What Makes It Green AIA Seattle
• Commercial Building of the year Design Build with FSC

Certifications

• Living Building Challenge
• FSC projet certification

ANALYSE GLOBALE DU PROJET

Dans le cadre de sa stratégie climatique, la ville de Seattle a mis en œuvre en 2009 le programme pilote Living Building en lien avec l'Institut Living Future. C'est dans ce contexte que la fondation Bullitt a souhaité ériger un bâtiment à usage mixte, avec un très fort engagement écologique. Face aux contraintes que ce type de bâtiment engendrait, il a été décidé d'en faire un bâtiment à usage commercial.

Situé proche de l'Ecodistrict de Seattle, le Bullitt center a été érigé à l'intersection de deux quartiers résidentiels. Initialement le terrain était occupé par un restaurant qui a été déconstruit et dont les matériaux ont été réemployés, réutilisés, ou recyclés.
Durant l'élaboration de ce projet la Fondation Bullitt a étroitement collaboré avec la ville de Seattle. La construction du bâtiment a d'ailleurs fait évoluer le code de l'urbanisme et de l'architecture en matière d'environnement et de développement durable, pour permettre la mise en place de technologies disruptives pour assurer la performance écologique du bâtiment.

Livré en 2013 le Bullitt Center obtient cette même année la certification Living Building Challenge (LBC), faisant de lui le troisème bâtiment au monde à l'obtenir. À ce jour, cinq organismes occupent le site : Bullitt Center foundation, Intentional futures, International Living Future Institut, PAE SKL Architect, et le Center for integrate design de l'Université de Washington.
Afin de respecter les conditions d'obtention de la certification Living Building Challenge, l'ensemble des locataires s'engagent à respecter les plans de consommation d'eau et d'énergie établis par la fondation Bullitt, suivis par des indicateurs de performance.

Objectifs

En s'inspirant des processus écologiques du Pin de Douglas et des forêts qu'il constitue, le Bullitt Center tente de restaurer les services écosystémiques préalablement existants sur le site du projet. L'objectif est d'édifier un bâtiment régénératif, ayant une empreinte écologique, sociale et économique positive, et ce en tenant compte de l'ensemble de son cycle de vie.
Construit pour durer 250 ans le bâtiment est conçu pour s'adapter au cours des décennies à son environnement. Afin de rendre la demarche réplicable, la Fondation Bullitt a rendu public l'ensemble des informations relatives à sa conception. Les principaux objectifs du projet en termes de développement durable sont ici listés:

1. Vers une autonomie énergétique

Afin de répondre à ses propres besoins énergétiques plusieurs technologies et innovations ont été utilisées en synergie au sein du Bullitt Center.

• Panneaux solaires :
• 574 panneaux solaires disposés sur le toit du bâtiment sur une surface de près de 1300 m² ;
• 257 000 Kwh : la quantité d'énergie que peut produire ces panneaux solaires ;
• 230 000 Kwh : la consommation La consommation annuelle électrique du bâtiment ;
• Toute surproduction électrique est réinjectée au sein du réseau électrique de la ville.

• L'enveloppe du bâtiment :
• Composée essentiellement de fenêtres triple vitrage, l'enveloppe permet au bâtiment de bénéficier d'une source de lumière naturelle ;

• 82% : le taux de luminosité naturelle que le bâtiment perçoit en moyenne au cours d'une journée ;
• L'orientation NO SE permet au bâtiment de recevoir, tout au long de la journée et de l'année, un gain de chaleur solaire ainsi qu'un éclairage naturel renforcé ;
• Ventilation récupératrice de chaleur : grâce à un système de roues alvéolées, intégrées aux fenêtres de l'enveloppe, la chaleur s'échappant du Bullitt Center est récupérée afin de préchauffer l'air entrant .

• L'énergie géothermique :
• 5 pompes à chaleurs captent, au sein de 26 puits, la chaleur du sol ;
• Système de chaleur rayonnante fonctionnant en boucle fermée, ce circuit régule la température du bâtiment (en cas de fortes températures ce système de chauffage peut fonctionner en sens inverse).

• Réduire la facture énergétique du bâtiment :
• Transformer l'énergie cinétique du freinage en énergie électrique : lors de son freinage l'ascenseur transforme l'énergie cinétique en énergie électrique réduisant ainsi sa facture électrique de près de 60% ;
• Ordinateur portable : l'ensemble des ordinateurs que le Bullitt Center met à la disposition sont portables, consommant ainsi moins d'électricité qu'un poste fixe ;
• Favoriser une activité physique : les utilisateurs du Bullitt Center sont vivement encouragés à emprunter les escaliers du bâtiment à la place de l'ascenseur, pour cela les escaliers sont mis en valeur dans le projet.

2. Vers une autonomie en eau

• La collecte de l'eau pluviale : collectée à la surface des panneaux solaires l'eau de pluie est stockée dans une citerne pouvant contenir plus de 200 000 litres d'eau. Tout surplus d'eau est réinjecté dans le réseau hydraulique de la ville.

• Traitement de l'eau : afin de potabiliser cette eau, des systèmes de filtrations par céramique et charbon actif ont été installés au sein du site. Par mesure d'hygiène une petite dose de chlore est utilisée pour purifier l'eau.

• Chauffage de l'eau : une des 5 pompes à chaleur dont dispose le Bullitt Center, est dédiée au chauffage de l'eau des robinets et des douches.

• Les toilettes : pour consommer très peu d'eau, les toilettes s'inspirent du système des toilettes sèches.

• Traitement des eaux usées : avant de réintégrer son milieu naturel les eaux usées sont biologiquement traitées. Les eaux grises sont traitées par un système de phytoépuration, ainsi qu'un traitement UV. Les eaux noires sont, quant à elles, compostées avant d'être utilisées comme fertilisant.

3. Un bâtiment sain & esthétique

• Bâtiment sain : le projet a respecté la liste rouge des matériaux potentiellement nuisibles de la certification LBC, ainsi l'équipea interdit l'usage de près de 360 substances chimiques au sein de ses matériaux.

• Un bâtiment esthétique : conçu pour être « beau », la conception formelle, esthétique, assure aussi au Bulitt Center son intégration au sein du paysage urbain.

• Une conception biophilique : procure aux utilisateurs du Bullitt Center un sentiment de bien-être et de lien avec la nature.

4. Vers un bâtiment durable dans le temps

Dans le cadre d'une analyse fine du cycle de vie, la fin de vie de ce bâtiment a été minutieusement évaluée. Il a, par ailleurs, été conçue pour une longue durée.

• **Structure :** la structure, composée de bois, de béton et d'acier, est conçue pour durer 250 ans.
• **Enveloppe :** l'enveloppe du Bullitt Center est conçue pour vivre entre 50 et 75 ans.
• **Panneaux solaires :** les panneaux solaires installés sur le toit ont une durée de vie d'environ de 25 ans.
• **Réparable :** constitué de plusieurs couches facilement désassemblables le Bullitt Center a été conçu pour être facilement réparable, mais aussi s'adapter aux variations climatiques à venir.

5. Vers un bâtiment à mobilité douce

Dans le cadre de sa stratégie de « mobilité douce » le Bullitt Center dispose seulement de parkings pour les vélos, incitant les utilisateurs du site à pratiquer une activité plus régulière en se rendant à leur lieu de travail à pied ou à vélo. Il contribue ainsi à la décongestion de la ville de Seattle et incite à une réduction de la dépendance aux énergies fossiles.

ANALYSE BIOMIMÉTIQUE

Conception biomimétique

Afin de concevoir un bâtiment « vivant » l'équipe en charge du Bullitt Center s'est tout particulièrement inspirée du Pin de Douglas et de son écosystème. Cette espèce endémique des forêts du Nord-Ouest des Etat-Unis et du sud-ouest du Canada a apporté les sources d'inspiration suivantes :

• **Les racines :** afin d'assurer le chauffage du bâtiment, le circuit de chaleur rayonnante traverse les planchers de chaque étage, il s'inspire du système racinaire de ce pin ;

• **La canopée :** comme une canopé, le toit du Bullit Center utilise l'énergie solaire pour produire l'électricité ;

• **Le tronc d'arbre & les branches :** le bois utilisé a un rôle structurel dans le bâtiment et fonctionne aussi comme une stratégie de stockage de carbone ;

• **Le cycle naturel de l'eau :** afin d'être autonome et auto-suffisant en eau, l'équipe en charge de la conception du bâtiment s'est inspirée du cycle de l'eau établi au sein des forêts de Pins de Douglas, avec des processus de captation, stockage et épuration ;

• **Dégradation de la matière organique :** avant de réintégrer leur milieu naturel les eaux noires du Bullitt Center sont compostées, ce système de compostage s'inspire du cycle de la dégradation de la matière organique instauré au sein des forêts ;

• **Nid d'abeille :** s'inspirant du nid d'abeille, un système de roue alvéolée disposé au sein des fenêtres du bâtiment assure sa ventilation et récupère aussi la chaleur s'évacuant du Bullitt Center.

Impacts documentés du projet

Ecotrust estime qu'au cours de son cycle de vie le projet engendrerait grâce à son écoconception un bénéfice économique s'élevant à près de 18 450 000 $ pour la societé. Les principaux impacts positifs sont :

• Mobilité :
• 160 tonnes de CO_2 evitées/an grâce à la politique de mobilité douce instaurée au sein du site ;
• 2 930 000 $ d'externalités environnementales evités au long du cycle de vie du bâtiment grâce aux stratégiés de mobilité douce.

• Compostage des eaux noires :
• 2,7 tonnes/an de compost produit ;
• 1,4 millions litres d'eau/an économisés au sein des toilettes du Bullitt Center ;
• 680 000 $ d'externalités environnementales evités au long du cycle de vie du bâtiment grâce aux stratégies de traitement des eaux noires.

• Efficience énergétique :
• 323,5 tonnes CO_2 évitées au cours du cycle de vie du bâtiment grâce à son efficience énergétique ;
• 10 270 000 $ d'externalités liées à la consommation énergétique évités au cours de son cycle de vie.

• Eau :
• 487 561 litres d'eau collectés et utilisés au sein du site ;
• 380 812 litres d'eaux grises réintégrent le milieu naturel par réinfiltration et évaporation après traitemet ;
• 197 219 litres d'eau réintégrant leur milieu naturel par évapotranspiration ;
• 910 000 $ de pollution évités au cours du cycle de vie du bâtiment.

• Énergie photovoltaïque :
• 101,6 tonnes CO_2/an évitées grâce à l'utilisation de cette énergie renouvelable ;
• 3 280 000 $ d'externalités environnementales evités au long du cycle de vie du bâtiment grâce aux stratégies de production d'énergie renouvelable.

• Bois & matériaux :
• 545 tonnes de CO_2 stockés dans l'ossature en bois du Bullitt Center jusqu'à sa fin de vie ;
• 370 000 $ d'externalités environnementales evités grâce aux stratégies de stocake de CO_2.

DÉMARCHE BIOMIMÉTIQUE

Approche
○ Problem driven
● Solution based

Origine de la bio-inspiration
● Opportunité/hasard/intérêt personnel
○ Collaborations interdisciplinaires
○ Appel à projet
○ Autre

Performances ciblées
● Confort thermique
● Confort visuel/lumineux
○ Confort acoustique
● Qualité de l'air intérieur
● Qualité de l'air extérieur
○ Résistance aux contraintes mécaniques
● Gestion de l'eau intérieur
● Gestion de l'eau extérieur
○ Accueil de la biodiversité
● Adaptation au climat
○ Allègement de la structure
● Gestion des déchets
● Autres : contribution à la résilience du quartier

Échelle d'intégration
○ Matériaux
○ Technologie
○ Système façade/toit/sol
● Bâtiment
○ Îlot
○ Quartier

PROCESSUS DE CONCEPTION

Utilisation d'un cadre de conception
● Oui
○ Non

Contraintes majeures
● Manque de fonds
○ Utilisation d'outils biomimétiques
● Règlementations
○ Problèmes techniques
○ Autres

Complexité de la conception
● Haute
○ Faible

LIEN AVEC LA BIOLOGIE

Niveau d'inspiration
● Organisme
○ Comportement
● Écosystème

Dimension de l'inspiration
○ Forme
○ Matériaux
○ Constructions/structure
● Processus
● Fonction

Types de modèles inspirants
○ Animal
● Plante : Pin de Douglas
○ Champignon
○ Archea/Bacteria
○ Protozoaire
○ Chromista
● Écosystème : Forêt de Pin de Douglas

Type de connaissance utilisée
○ Existantes pour le grand public
● Existantes pour le spécialiste
○ Créées pendant la conception

Type d'apports en biologie
○ Historique/savoir du concepteur
● Acquisition lors de la conception
● Biologistes intégrés au processus

RÉSULTATS & INNOVATION

Complexité de la construction
○ Haute
● Faible

Niveau d'innovation
● Innovation de rupture
● Amélioration de l'existant

Revenir à la liste des projets

LEÇONS À RETENIR

• L’enveloppe du bâtiment constitue la source principale de lumière du bâtiment évitant ainsi de surcharger des plafonds en luminaires. Cette source de lumière naturelle offre aussi au bâtiment un certain confort et bien-être pour ses utilisateurs.

• Pour améliorer l’efficience et la performance écologique du bâtiment, un système d’évaluation environnementale été mis en place au sein du site afin de quantifier les flux de ressources et matières. L’avis des utilisateurs du Bullitt Center a aussi été pris en compte afin de compléter cette évaluation.

• Afin de s’assurer du bon fonctionnement d’un bâtiment comme le Bullitt Center, il est préconisé de recruter un ingénieur à temps plein. Ce dernier a pour mission de faire le lien entre les usagers et les technologies installés au sein du bâtiment.

• Un système informatique est installé au sein du Bullitt Center dans l’objectif de gérer automatiquement le confort lumineux et thermique du bâtiment. Cependant, certains utilisateurs du site se sont plaints de ne pas pouvoir le gérer manuellement.

• Afin d’assurer un éclairage et une ventilation naturels des bureaux, l’équipe en charge de la construction du bâtiment a privilégié l’aménagement d’espaces de travail en *open-space*.

• Le toit du Bullitt Center porte des panneaux solaires et des puits de lumière. Afin d’optimiser ces flux d'énergies lumineuse et électrique, il a fallu minutieusement étudier l’orientation et l’agencement du toit.

• Un bâtiment à haute performance environnementale constitue un coût financier lors de sa construction, néanmoins une forte plus-value à moyen et long terme et un fort retour en termes de qualité de vie et impacts positifs pour la societé a été observé.

• Les impacts sociaux et environnementaux positifs du projet sont importants et valorisés comme argument commercial.

• Se limiter aux ressources disponibles sur site a été une stratégie clé dans la conception du batimênt.

Crédits des images :

[1] [3] CC BY-SA 3.0 by Joe Mabel

[2] Public Domain by the United States Department of Energy

[4] CC BY-SA 3.0 by Walter , [4] CC BY-NC 2.0 by Brad Kahn

[1]

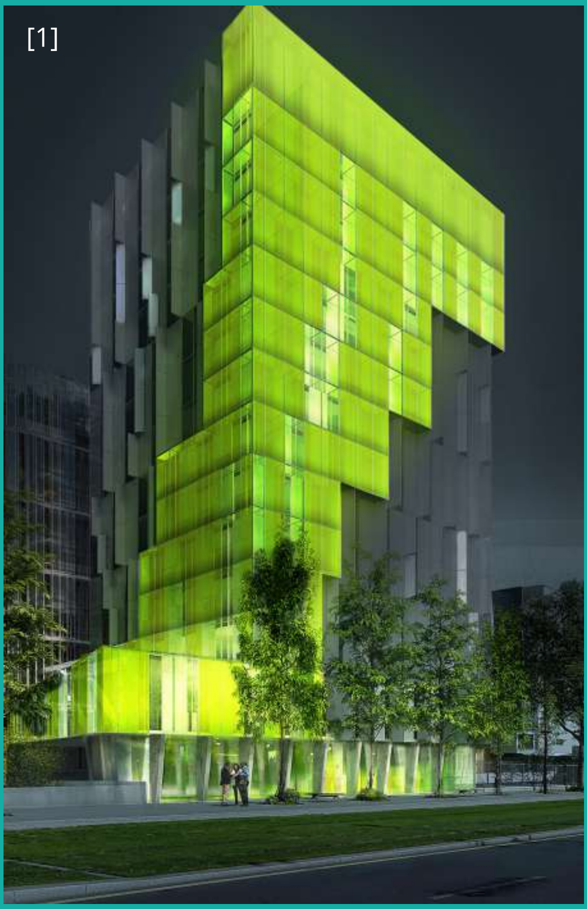

ALGUESENS

Adresse : Paris Rive gauche 13e arrondissement Boulevard des Maréchaux
Statut du projet : Projet accepté
Date de conception : 2016
Type/usage : Logements
Rénovation : Non
Climat : Cfb - climat tempéré chaud, sans saison sèche et à été tempéré
Surface du projet : 17 450 m^2 dont 13 000 m^2 de surface habitable
Coût de construction : -
Coût final du projet : 37 000 000 €

RÉSUMÉ

Situé à l'entrée sud du quartier Masséna dans le 13e arrondissement de Paris, le projet architectural Alguesens est en 2016 l'un des 22 lauréats du concours Réinventer Paris.
Composé de trois bâtiments, dédiés à des usages anthropiques, l'Algo House, la Plant House, la Tree House, ainsi qu'un bâtiment dédié aux lombrics, la Lombric House, le projet, une fois livré, sera une oasis de verdure au sein d'un écosystème urbain dense. Lieu de mixité sociale et d'innovation, Alguesens accueillera notamment au sein de son site une résidence ouverte aux étudiants et jeunes chercheurs, des logement locatifs intermédiaires en accession, un café alternatif, un laboratoire de recherche et d'innovation ainsi que des jardins et potagers. Alguesens comptera sur son site près de 295 logements : 49% d'entre eux seront en accession libre contre 23% de logements intermédiaires. Les 28% restant seront réservés à de la location, à destination de jeunes chercheurs. Alguesens comptera aussi au sein de son site 1092 m^2 de tiers lieu et 250 m^2 de commerces. Lieu de mixité sociale et d'innovation le projet Alguesens a aussi pour ambition de faire vivre en symbiose l'Humain et la nature.
Les architectes du cabinet XTU et MU, en concevant ce projet, ont pour objectif de réintégrer le vivant en ville tant pour des raisons écologiques que sociales. Alguesens est à la fois un lieu nourricier, dans le sens premier du terme, mais aussi dans le sens intellectuel du terme. En effet, en faisant de ce lieu un espace d'échange, de rencontre et de collaboration l'équipe en charge de la conception du projet souhaite faire de ce lieu un espace d'innovation et d'incubation.
Construit dans un quartier universitaire Alguesens est surtout connu pour la plus grande bio-façade du monde, constituée de micro-algues et installée sur l'une des façades de l'Algo House. Ce bâtiment sera, notamment pour l'Université Paris Descartes, un bâtiment vitrine mettant en lumière une culture verticale de micro-algues destinée à la recherche médicale.
Dans le cadre de l'analyse biomimétique de ce projet nous nous concentrerons principalement sur l'Algo House.

Revenir à la liste des projets

Rôle des principaux participants

Maîtrise d'ouvrage : Ville de Paris et BDP Marignan – SNI (financeurs)
Architectes : XTU architects et MU architects

Coûts

Coût final du projet : 37 000 000 €

Principaux prix

Sans objet

Certifications

• Certification NF Habitat HQE niveau Excellent visée

[1]

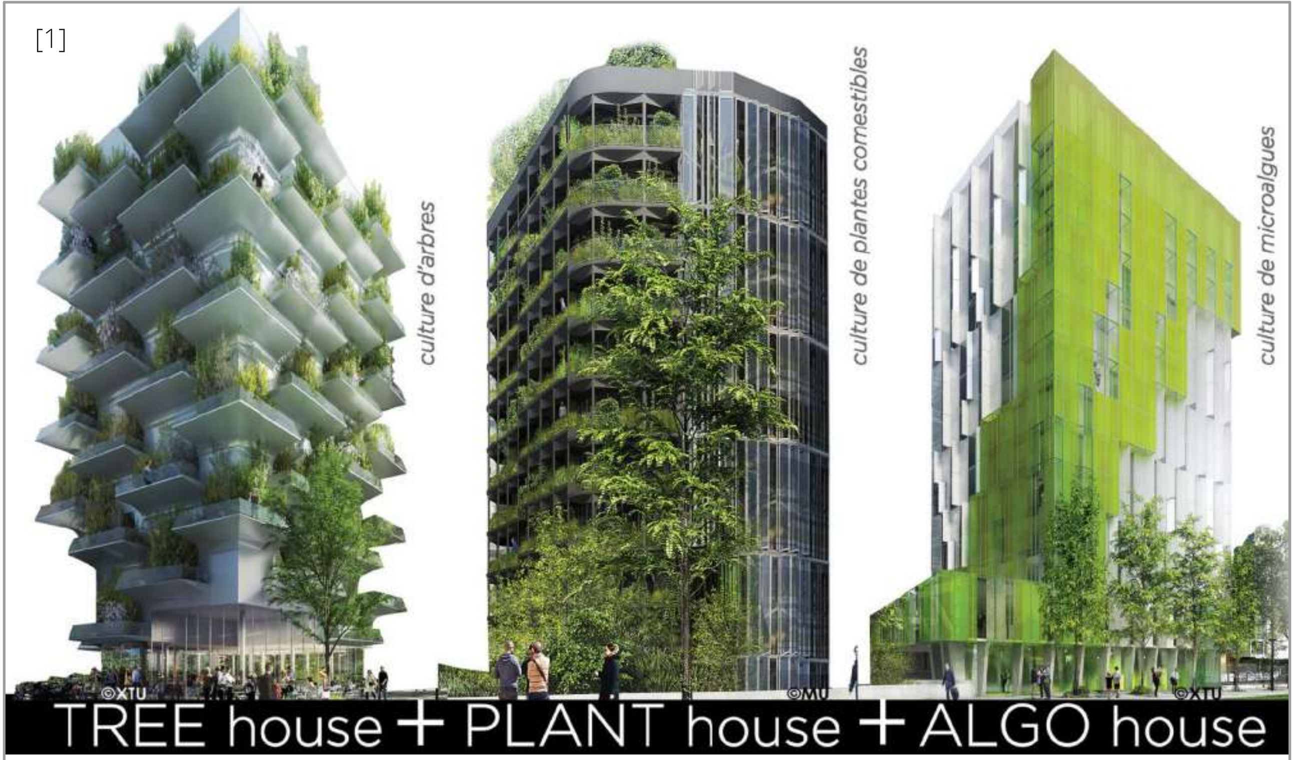

ANALYSE GLOBALE DU PROJET

Lauréat du concours Réinventé Paris le projet d'innovation urbaine Alguesens cherche, en recueillant la biodiversité en ville, à faire vivre en symbiose, l'humain et la nature au sein de site. Ce projet urbain biomimétique invite aussi à faire de son site un lieu de mixité sociale grâce à un complexe urbain se composant de trois bâtiments intelligents composés notamment de logements et d'espaces de recherche et d'accueil de la biodiversité :

• **L'Algo House :** résidence destinée aux jeunes chercheurs et étudiants, ce bâtiment accueillera sur sa façade la plus ensoleillée une culture verticale de microalgues, *Haematococcus pluvialis*. Cette bio façade, jouant le rôle de double peau, assura la régulation thermique du bâtiment et de dépollution en captant le CO_2 présent dans l'atmosphère nécessaire à l'accroissement des algues. Nous allons dans le cadre de cette présentation nous concentrer tout particulièrement sur cette bio-façade.

• **Tree house :** Ce batiment hébergera des logements intermédiaires en accession ainsi qu'un tiers lieu destiné à l'incubation de projet d'innovation du vivant.

• **Plant house :** cet immeuble a pour volonté d'être un terrain de jeux pour l'agriculture urbaine puisqu'il accueillera près de 2 000 m^2 de jardins et potagers en plus de ses logements locatifs intermédiaires.

La maîtrise d'œuvre souhaite grâce à ce projet revitaliser le quartier universitaire de Paris Descartes en aménageant une oasis urbaine allant dans la continuée de la trame verte construite entre le 13e arrondissement et la ville d'Ivry.

Objectifs

Face à une urbanisation galopante et dense de nos villes, la maîtrise d'œuvre a souhaité, selon Anouk Legendre, cofondatrice de l'agence XTU, faire de ce projet urbain un lieu favorisant : la mixité et l'ouverture entre citadins, l'incubations et l'innovations de projets biologiques, de réintégration du vivant en ville ; dans le but de tendre vers une société plus juste, durable et résiliente.
Le cahier des charges du concours réinventer Paris préconisait initialement la construction au sein de ce site d'une barre d'habitations, néanmoins la maitrise d'ouvrage a elle fait le choix de concevoir plusieurs bâtiments, ouverts sur le quartier en constituant des failles architecturales. De plus, les innovations techniques et technologiques initiées au sein de ce projet invitent à la réintégration d'une biodiversité, en ville sur les toits, façades et balcons des bâtiments et ne demandant que très peu d'entretien.
Par ailleurs, avant que l'Algo House, bâtiment phare de ce projet, ne voit le jour le cabinet XTU a travaillé avec des entreprises et laboratoires de recherche afin de développer un projet pilote de bio-façade de micro-algues, breveté, nommé SymBIO2. Le premier projet avoir bénéficier de cette technologie l'un des bâtiments du CBST construit au Champs-sur-Marne construit en 2016.

Revenir à la liste des projets

Conception biomimétique

Dans l'analyse de la conception biomimétique de ce projet nous nous concentrerons ici plus particulièrement sur Algo House. Ayant besoin de lumière pour croître, cette culture de microalgues verticale, sera installée sur la façade la plus ensoleillée du bâtiment. En développant une culture de ce phytoplancton au sein de son projet, la maîtrise d'œuvre participe à la production d'oxygène élément nécessaire, notamment, à la vie humaine.

• **Puits de carbone :** afin de croitre, les microalgues cultivées au sein de la bio-façade, vont avoir besoin de CO_2. Cette bio-façade participera ainsi à la lutte contre le rechauffement climatique.

• **Régulation thérmique :** en cultivant ces phyto-bioréacteurs, les architecte XTU assurent une régulation thermique naturelle du batiment.

• **Une réduction de 50% des consommations énergétiques du bâtiments :** en valorisant la chaleur captée par les photobioréacteurs pour chauffer le bâtiment et l'eau des sanitaires, la bio-façade de l'Algo House permettra de réduire la consommation énergétique du bâtiment sous le seuil de 48 kHW/m^2 du Plan Climat de Paris.

• **Une façade laboratoire :** une fois arrivé au terme de leur croissance (après 24h à 48h) les microalgues seront collectées afin d'être utilisées dans des projets de recherche médicale.

• **Une bio façade de plus de 900 m^2 :** la bio-façade Symbio2 installée au sien de l'Algo House, technologie brevetée par le cabinet d'architecte XTU, se verra être la plus grande bio-façade construite à ce jour par le consortium d'acteurs ayant participé à l'élaboration de cette innovation.

• **Vers une symbiose :** de ce fait le bâtiment et cette bio-façade sont interdépendants. Alors que les algues ont besoin de la chaleur émise par le bâtiment pour croître, l'Algo House dépend elle de cette bio-façade pour sa régulation thermique.

Impacts sur la recherche

Le Projet SymBIO2 est le résultat d'un projet d'innovation collaboratif, initié en 2007, regroupant une équipe d'acteurs pluridisciplinaires, dont, le laboratoire GEPEA, AlgoSource, Séché Environnement, Viry, R.F.R, OASIIS, Kalitec Génie climatique, le CSTB et XTU. Avant de déployer cette technologie sur l'une des façades de l'Algo House un projet pilote a été installé au sein de l'une des façades du Centre Scientifique & Technique du Bâtiment (CSTB) à Champ-sur-Marne.

DÉMARCHE BIOMIMÉTIQUE

Approche
- ○ Problem driven
- ● Solution based

Origine de la bio-inspiration
- ● Opportunité/hasard/intérêt personnel
- ● Collaborations interdisciplinaires
- ○ Appel à projet
- ○ Autre

Performances ciblées
- ● Confort thermique
- ○ Confort visuel/lumineux
- ○ Confort acoustique
- ● Qualité de l’air intérieur
- ○ Qualité de l’air extérieur
- ○ Résistance aux contraintes mécaniques
- ● Gestion de l’eau intérieur
- ● Gestion de l’eau extérieur
- ● Accueil de la biodiversité
- ○ Adaptation au climat
- ○ Allègement de la structure
- ● Gestion des déchets
- ○ Autres : performance énérgétique, s. culturels

Échelle d'intégration
- ○ Matériaux
- ● Technologie
- ● Système façade/toit/sol
- ○ Bâtiment
- ○ Îlot
- ○ Quartier

PROCESSUS DE CONCEPTION

Utilisation d'un cadre de conception
- ● Oui
- ○ Non

Contraintes majeures
- ○ Manque de fonds
- ○ Utilisation d'outils biomimétiques
- ○ Règlementations
- ● Problèmes techniques
- ● Autres

Complexité de la conception
- ● Haute
- ○ Faible

LIEN AVEC LA BIOLOGIE

Niveau d'inspiration
- ● Organisme
- ○ Comportement
- ○ Écosystème

Dimension de l'inspiration
- ○ Forme
- ○ Matériaux
- ○ Constructions/structure
- ● Processus
- ● Fonction

Types de modèles inspirants
- ○ Animal
- ● Plante
- ○ Champignon
- ○ Archea/Bacteria
- ○ Protozoaire
- ○ Chromista
- ● Écosystème

Type de connaissance utilisée
- ● Existantes pour le grand public
- ● Existantes pour le spécialiste
- ● Créées pendant la conception

Type d'apports en biologie
- ● Historique/savoir du concepteur
- ● Acquisition lors de la conception
- ● Biologistes intégrés au processus

RÉSULTATS & INNOVATION

Complexité de la construction
- ● Haute
- ○ Faible

Niveau d'innovation
- ○ Innovation de rupture
- ● Amélioration de l'existant

LEÇONS À RETENIR

• La bio-façade intégrée au sein de l'Aglo House a été soumise à des tests d'acceptabilité sociale avant d'être déployée . En effet, la culture verticale d'algue au sein des façades de nos bâtiments n'est pas chose courante dans nos villes. Il a donc fallu, au préalable, s'assurer que les citoyens soient favorables à l'émergence de tels dispositifs.

• Le projet Alguesens et le projet de bio-façade Symbio2 ont vu le jour grâce à une collaboration entre des acteurs pluridisciplinaires. Ce projet urbain met ainsi en avant l'intérêt de mener des projets d'innovation collaboratifs afin d'en faire des projets fonctionnels, biomimétiques et assurant une certaine mixité sociale.

• Alors que nos villes doivent faire face à une pression démographique galopante et une urbanisation dense, le projet Alguesens assure la réintégration du vivant au sein de nos écosystèmes urbains.

Crédits des images :

[1] © XTU architects

[2] © XTU + MU

Revenir à la liste des projets

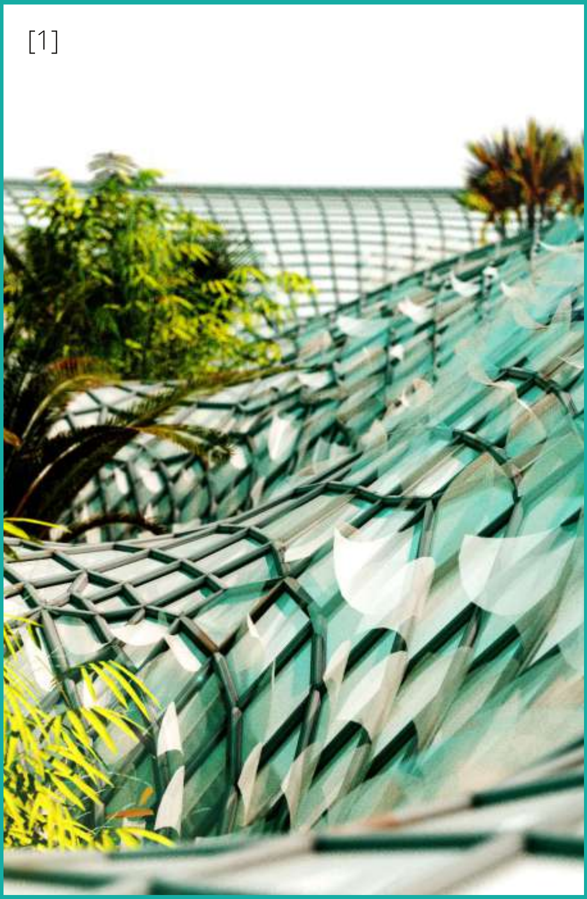

[1]

BANGKOK, I'M FASHION HUB

Centre de la mode Thaï

Adresse : 1035 Phloen Chit Rd, Lumphini, Pathum Wan District, Bangkok 10330, Thaïlande
Statut du projet : projet non accepté
Date de conception : 2015
Type/usage : centre d'évènements
Rénovation : non
Climat : AW - climat de savane à hiver sec
Surface du projet : 4345 m² sdp, 3495 m² shab
Coût de construction : 1 900 €/m²
Coût final du projet : -

Membres du Biomim'City Lab engagés :

TANGRAM ARCHITECTES

RÉSUMÉ

Le projet consiste en la création d'un centre dédié à la mode thaï et à l'émergence de la jeune scène artistique. Il est le résultat d'une hybridation entre architecture, paysage et espace public : les fonctions du programme qui s'égrènent le long d'un podium de défilé à pente continue en double hélice scandent un parcours fluide en triplex pour connecter le sol avec le métro et ses trottoirs suspendus. Ainsi, le projet s'apparente tout autant à un parcours public en spirale, parsemé de mezzanines et de jardins suspendus, qu'à un projet d'architecture.

De ce fait des arbustes, essences locales fournissant des pigments naturels, sont plantés dans des jardins suspendus permettent la teinture naturelle des fibres textiles produites sur place grâce à la captation du CO_2 réalisée par la toiture. La membrane supérieure de la toiture à effet bi-métal, assure tantôt l'étanchéité, tantôt la ventilation naturelle.

Les eaux de pluie sont récupérées dans des *containers* pour la production de fibres textiles ainsi que pour alimenter des jardins d'eau. L'évaporation de l'eau au sein de ces jardins permet ainsi le rafraîchissement naturel du site. De plus un tissage structurel en bambou, contribuant à la ventilation et au rafraîchissement naturels des espaces, ainsi qu'à la filtration du soleil, évoque la légèreté et la finesse des créations réalisées sur place.

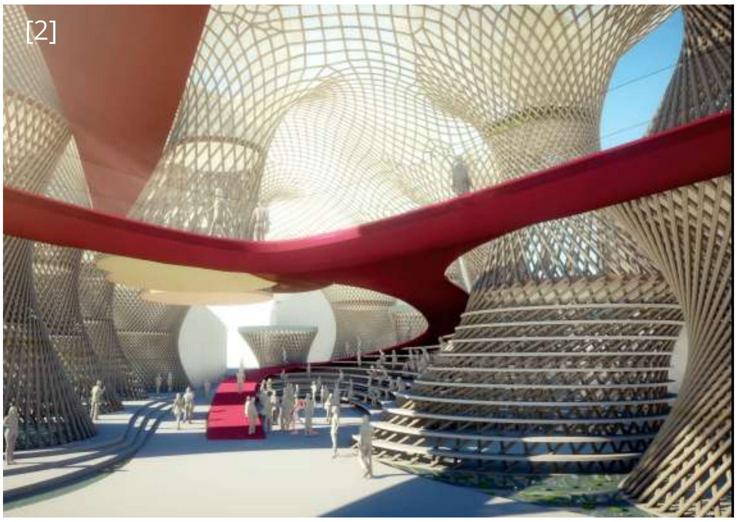

[2]

Rôle des principaux participants

Commanditaire : HMMD Architecture Competition

Architectes : Tangram Lab, Tangram Architectes, Treex

Coûts

Coût de construction : 1 900 €/m²

Principaux prix

Sans objet

Certifications

Sans objet

[2]

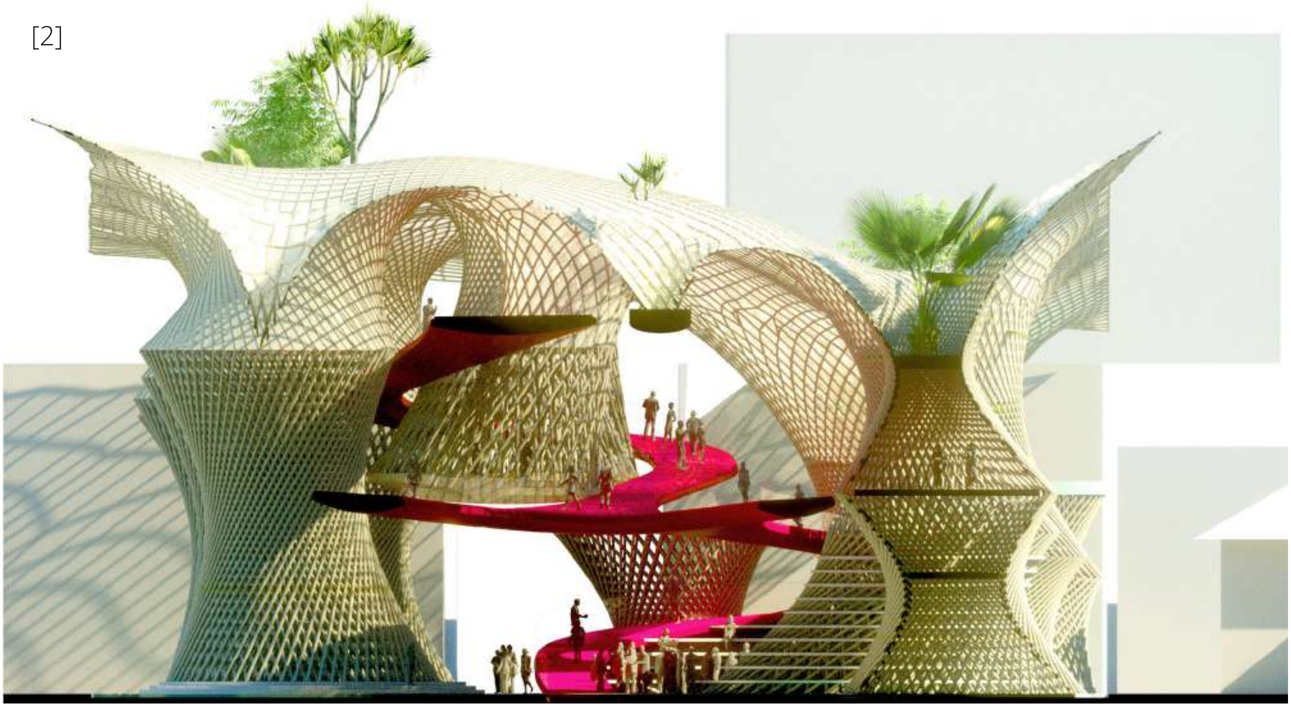

ANALYSE GLOBALE DU PROJET

Le projet consiste en la création d'un centre dédié à la mode thaï et à l'émergence de la jeune scène artistique. Il s'agit de créer un lieu pour mettre en avant les nouveaux talents (boutiques, défilés, lieux de rencontre et de formation). Situé dans l'hypercentre de Bangkok (station BTS Phloen Chit), le projet doit s'insérer dans une dent creuse entre des tours, une station de métro, des trottoirs suspendus, un reliquat pavillonnaire et l'omniprésence des voitures dans un mélange hétéroclite et coloré. La densité y est donc extrême et les contrastes morphologiques violents. La chaleur, l'humidité, le bruit et la pollution ambiantes confèrent à ce lieu un sentiment chaotique, d'étouffement.

Objectifs

Le projet entend répondre à 3 grands objectifs :

• **Suturer, relier une ville labyrinthique**, entremêlée, pluri-horizontale qui traduit la dynamique avec une ville agglutinée verticale, dont les innombrables gratte-ciels formant une *skyline* acérée traduisent la statique, l'immobilité. Le site du projet est un condensé de l'image de cette ville.

• **Choisir un matériau local, naturel**, durable, renouvelable et consommer un minimum de matière pour réaliser un lieu dédié à la découverte par le grand public des jeunes créateurs de mode thaï. Cet espace de mise en scène doit être ouvert, accueillant, stimulant et propice à la créativité.

• **Positiver des contraintes climatiques majeures** (chaleur, humidité, pollution de l'air) et liées au programme (consommations d'eau et de CO_2 par l'industrie textile) pour créer un lieu peu consommateur d'énergie, naturellement rafraîchi et purificateur.

Conception biomimétique

L'approche biomimétique est née de la volonté de résoudre les contraintes fortes présentées dans les objectifs (climat + pollution, industrie textile, site). Elle a donné naissance à une peau multifonctionnelle :

• **Une surface minimale structurelle en bambou tissée, inspirée des toiles d'araignées :** assure la minimisation de l'emploi de matériau. La recherche de forme vise la récupération d'eau de pluie nécessaire à la fabrication de fibres synthétiques recyclables (transformation organocatalytique) sur place, à l'alimentation de jardins d'eau et à l'arrosage des plantes à pigments naturels accueillis dans les jardins suspendus. Le tissage de bambou a pour quête d'assurer la ventilation naturelle et la protection solaire. La forme générée abolit la frontière entre façades et toitures et sépare finement les espaces intérieurs et extérieurs dans un tout continu.

• **Une membrane à effet hydro bimétal, inspiré de la pomme de pin :** permet d'assurer l'étanchéité et la ventilation naturelle passives. Cette membrane capte le CO_2 dans l'air pour produire des fibres textiles. L'effet lotus a également été étudié pour la récupération de l'eau de pluie.

• **Différentes plantes locales étudiées dans le cadre de ce projet :** dont notamment le *Costus claviger* pour l'enroulement spiralé, essences à pigments naturels et pour le traitement phytosanitaire.

Impacts documentés du projet

Les impacts potentiels visés par ce projet sont :

• **Créer un lieu qui relie**, unisse, différents espaces publics à différentes altimétries selon un parcours animé par des fonctions variées. Transformer un usage quotidien en expérience ludique et récréative grâce à une forme bâtie hybride réunissant architecture, espace public et paysage dans un tout continu.

• **Transformer des problèmes** (chaleur, humidité, pollution) en opportunité.

• **Développer une filière locale et durable** de production de matière première (construction, textile synthétique et pigments).

• **Créer un environnement paysager** avec des jardins d'eau pour créer un îlot de fraîcheur urbain.

Impacts sur la recherche

Cette tentative de tissage continu en bambou a débouché sur un projet de cité scolaire internationale actuellement en cours de construction à Yangon, Birmanie. Pour mener à bien ce projet de façades filtrantes en bambou, Tangram Architectes a organisé une filière locale complète comprenant :

1. Recherche en campagne de bambous compatibles avec la construction ;

2. Études des différents types de traitement du bambou ;

3. Recherche et formation d'entreprises locales avec mise au point de détails techniques d'assemblage adaptés aux compétences in situ (bambou/acier) ;

4. Réalisation de prototypes en ateliers et sur site ;

5. Construction du projet (en cours de finalisation).

DÉMARCHE BIOMIMÉTIQUE

Approche
● Problem driven
● Solution based

Origine de la bio-inspiration
● Opportunité/hasard/intérêt personnel
○ Collaborations interdisciplinaires
○ Appel à projet
○ Autre

Performances ciblées
● Confort thermique
● Confort visuel/lumineux
○ Confort acoustique
● Qualité de l’air intérieur
● Qualité de l’air extérieur
● Résistance aux contraintes mécaniques
○ Gestion de l’eau intérieur
● Gestion de l’eau extérieur
○ Accueil de la biodiversité
● Adaptation au climat
● Allègement de la structure
● Gestion des déchets
○ Autres

Échelle d'intégration
● Matériaux
○ Technologie
● Système façade/toit/sol
○ Bâtiment
● Îlot
○ Quartier

PROCESSUS DE CONCEPTION

Utilisation d'un cadre de conception
● Oui
○ Non

Contraintes majeures
○ Manque de fonds
○ Utilisation d'outils biomimétiques
○ Règlementations
● Problèmes techniques
○ Autres

Complexité de la conception
● Haute
○ Faible

LIEN AVEC LA BIOLOGIE

Niveau d'inspiration
○ Organisme
○ Comportement
● Écosystème

Dimension de l'inspiration
● Forme
● Matériaux
● Constructions/structure
○ Processus
● Fonction

Types de modèles inspirants
○ Animal
● Plante
○ Champignon
○ Archea/Bacteria
○ Protozoaire
○ Chromista
● Écosystème

Type de connaissance utilisée
○ Existantes pour le grand public
● Existantes pour le spécialiste
● Créées pendant la conception

Type d'apports en biologie
○ Historique/savoir du concepteur
● Acquisition lors de la conception
○ Biologistes intégrés au processus

RÉSULTATS & INNOVATION

Complexité de la construction
● Haute
○ Faible

Niveau d'innovation
● Innovation de rupture
○ Amélioration de l'existant

Revenir à la liste des projets

LEÇONS À RETENIR

Les enseignements de ce projet résident dans la conception d’une surface minimale composite bambou (tissage) / acier (pièces d’assemblage) qui répond à des paramètres variés telles que la distribution ordonnée des fonctions du programme, la connexion d’espaces publics à différentes altimétries et l'intégration de l’eau et des plantations dans l’architecture.
Le projet réfléchit aussi à la mise en place d’un cycle énergétique vertueux en réponse à un climat et à une industrie textile contraignants.
Cette réponse permet d’abolir la frontière entre toiture et façade, de penser l’enveloppe du bâtiment comme un tout unifié multifonctionnel permettant de traîter la protection solaire, le rafraîchissement et la ventilation naturels passifs. Cela ouvre la voie à un autre rapport dedans/dehors, intérieur/extérieur.

Crédits des images :

[1] [2] [3] Treex

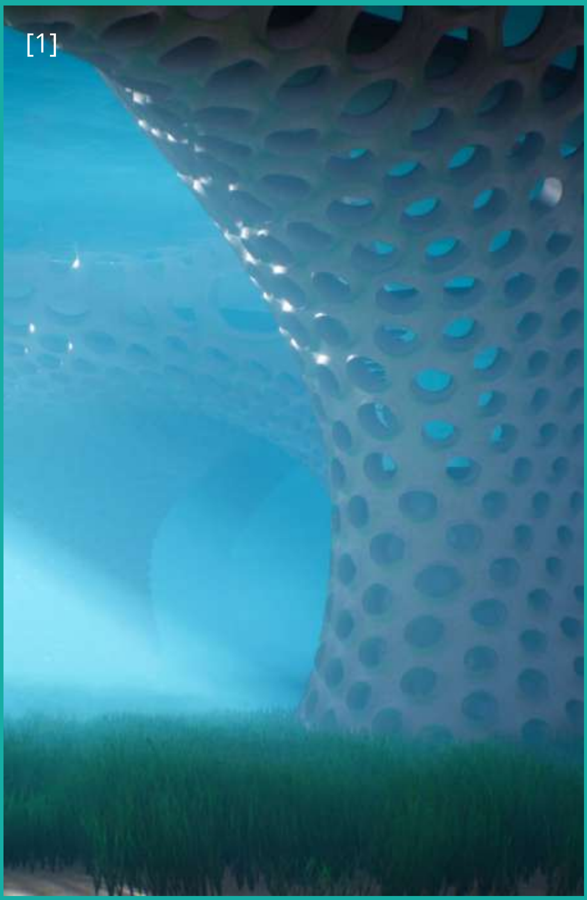

BIOLUM_REEF
Un récif habité éco-vertueux

Adresse : Île Ratonneau, Archipel du Frioul, Marseille, France
Statut du projet : projet conceptuel
Date de conception : 2017
Type/usage : lieux artistique, de recherche de sensibilisation grand public
Rénovation : non
Climat : CSA - climat tempéré chaud à été chaud et sec
Surface du projet : 16 500 m²
Coût de construction : -
Coût final du projet : -

Membres du Biomim'City Lab engagés :

TANGRAM ARCHITECTES

RÉSUMÉ

Biolum_Reef est un projet biomimétique de récif habité éco-vertueux qui poursuit 3 objectifs :

1. Favoriser l'essaimage de la biodiversité marine grâce à un récif artificiel. Polyfonctionnel et bioluminescent, il sera imprimé à partir de déchets plastiques collectés dans la mer. Le récif protégera le trait de côte de la houle, portée par le mistral et les vagues d'étrave des ferries. Il permettra une canalisation des bateaux en faveur d'une navigation propre et un mouillage en dehors des herbiers à posidonie.

2. Implanter, dans le récif, 3 habitats semi-immergés et autonomes dédiés à la collaboration avec les océans, à la création artistique et au partage de la connaissance. Ainsi, laboratoires scientifiques, départ d'explorations sous-marines, ateliers de design, fabriques de matériaux, traitement des déchets, amphithéâtres et salles d'exposition jalonneront le parcours aquatique du public.

3. Proposer un cycle éco-vertueux, energy free et dépolluant dans lequel les déchets sont des ressources. Les déchets organiques et plastiques, ainsi que les eaux noires, seront le moteur de la production de biomasse algale, d'éclairage bioluminescent, d'énergie renouvelable, d'eau potable, de sel, de nourriture variée et de l'impression 3D du récif. À mesure que nous dépolluons, nous créons les conditions d'une vie autonome ayant un impact positif sur l'environnement.

Rôle des principaux participants

Commanditaire : Fondation Jacques Rougerie / Institut de France

Architectes : Tangram Lab, Tangram Architectes, Treex, M.I.O - Institut Méditerranéen d'Océanographie (AMU, OSU Pyhtéas, CNRS, IRD)

Coûts

Sans objet

Principaux prix

2017 • Prix « Coup de Cœur » du concours international de la Fondation Jacques Rougerie / Institut de France, catégorie Architecture et Innovation pour la Mer.

Certifications

Sans objet

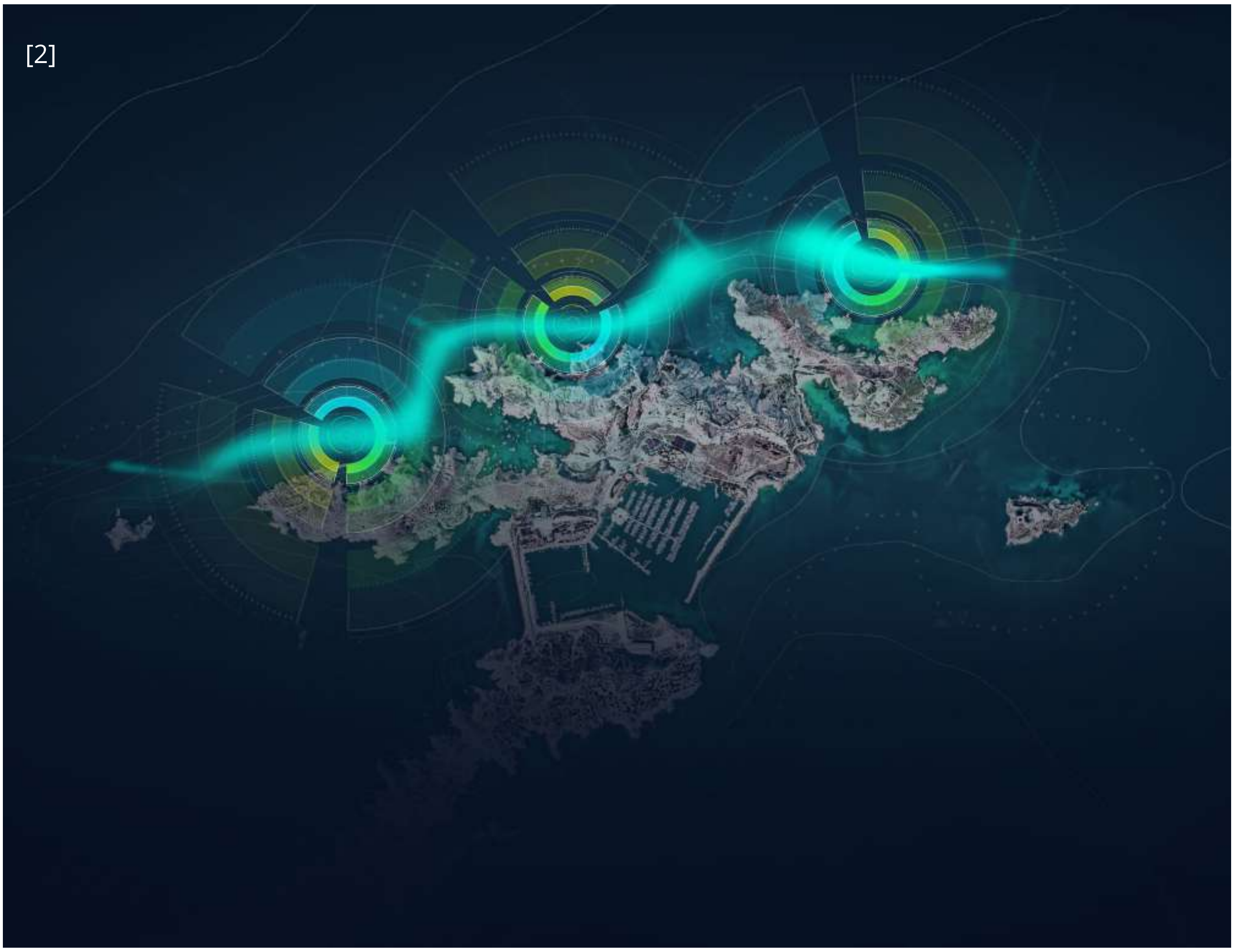

ANALYSE GLOBALE DU PROJET

La Fondation Jacques Rougerie / Institut de France organise chaque année un concours d'innovation et d'architecture pour le monde marin, afin d'encourager l'émergence de solutions innovantes en réponse aux grands défis du changement climatique et des conséquences qui en émanent.
L'archipel du Frioul est situé dans le Parc National des Calanques. Il est l'épicentre géographique de Marseille, la capitale mondiale de l'univers sous-marin. Une fragilisation de l'écologie terrestre et marine menace les îles du Frioul : la présence de nombreux bateaux de plaisance jetant l'ancre au hasard et de visiteurs peu scrupuleux de la faune et de la flore, fragilisent une biodiversité riche, dont certaines espèces endémiques. Déchets divers, arrachage des herbiers à posidonie, érosion du trait de côte, pollutions multiples mettent à bout de souffle un écosystème précieux. Le triptyque programmatique, découvrir – créer – transmettre, ambitionne de réunir les disciplines scientifiques et artistiques au service du plus grand nombre. Il s'incarne dans un récif, habité par une multitude d'espèces en son sein comme à sa périphérie, pour devenir un lieu de promenades sous-marines interactives.

Objectifs

Pour favoriser le développement de la biodiversité marine et assurer l'autonomie énergétique, un travail de form-finding de la peau du récif a été réalisé. Afin de ne pas impacter les conditions de vie la biodiversité terrestre et marine, les paramètres pris en compte sont variés : surface minimale pour alléger le poids et limiter l'impact au fond sur les herbiers à posidonie sans les priver de lumière, variations des dimensions de percements selon l'orientation (protection au vent, au soleil, oculus pour ventilation naturelle, grande fenêtre d'observation sous-marine, petits percements pour créer des niches écologiques), travail sur la rugosité (lisse en conditions terrestres, rugosités variables pour accroche de la biodiversité marine) et sur l'épaisseur selon les contraintes mécaniques.
Ce projet vise aussi à être autonome en énergie et à dépolluer son environnement : un cercle vertueux, en utilisant les déchets comme ressources, a été mis en place. Basé sur des bactéries bioluminescentes, des microalgues, le compost des déchets organiques, la production d'énergie houlomotrice et photovoltaïque, les déchets plastiques et les eaux sales, le projet envisage de produire de la matière première pour imprimer le récif, de fournir de l'eau propre, du sel, de la nourriture (poissons, algues), des compléments alimentaires (spiruline), du *bio-fuel*, de l'éclairage bioluminescent, de l'électricité, une ventilation et un rafraîchissement naturels passifs. Des « plots » techniques assurent ces fonctions. Ils sont connectés à la peau du récif par de grandes promenades muséographiques et éducatives.

ANALYSE BIOMIMÉTIQUE

Conception biomimétique

Cette conception biomimétique a été motivée par une volonté de résoudre un grand nombre de problématiques. Une approche écosystémique est rapidement apparue comme indispensable :

• **Une peau du récif inspirée de la structure minimale des coquilles d'oursin et la distribution de leurs épines :** générée de façon paramétrique et bio-inspirée, cette peau de récif s'adapte à ses conditions environnementales et se compose de percements répondant aux besoins, en termes d'habitats des espèces marines, ainsi qu'aux besoins humains pour leurs conditions d'habitat terrestres.

• Un récif corallien pour réduire la force des vagues et produire de l'énergie : le modèle des récifs coralliens a permis à la maîtrise d'œuvre de comprendre le rôle de ces écosystèmes dans le but de réduire la force des vagues et de ce fait protéger le trait de côte. Ceci tout en tirant parti de l'énergie de la houle pour de produire de l'électricité.

• Une symbiose entre les micro-algues et les bactéries bioluminescentes : les micro-algues couplées aux bactéries bioluminescentes ont été utilisées pour assurer le rafraichissement de l'air, la production des compléments alimentaires, la dépollution passive des eaux et la production d'une lumière naturelle nocturne.

• La posidonie un écosystème modèle : l'écosystème posidonie a également été riche d'enseignements pour établir un circuit fermé mais ouvert et branché sur son environnement pour concevoir un réservoir de biodiversité.

Impacts documentés du projet

Le projet entend apporter différents services et bénéfices :

• Développer la connaissance scientifique sur le monde marin : en offrant des espaces de travail *in situ* ainsi qu'un point de départ pour des explorations sous-marines ;

• Offrir des lieux inspirants pour toutes d'expressions artistiques créatives : ainsi, Biolum_Reef réunit en son sein des activités complémentaires et multidisciplinaires ;

• Créer un lieu de sensibilisation et de partage des connaissances acquises avec le grand public : un lieu d'accueil, de partage et d'enseignement ;

• Vivre en autonomie de manière responsable et vertueuse : en faisant des déchets les ressources ;

• Un récif tendant à croître à mesure qu'il dépollue la mer : ce projet vise à dépolluer le site de ses déchets (plastiques, chimiques) et s'en servir comme matière première pour imprimer le récif ;

• Se laisser coloniser : favoriser le développement d'une biodiversité riche, actuellement menacée, et ce en offrant des habitats variés et nombreux ;

• Reconnecter l'Humain et le vivant (non humain) : via une communion sous-marine, en proposant des promenades diurnes et nocturnes inédites (éclairées grâce à la bioluminescence).

Impacts sur la recherche

Ce projet a donné naissance a un partenariat de recherche entre le M.I.O et le CEAtech à Cadarache portant sur le couplage entre bactéries bioluminescentes et micro-algues. Les recherches ont abouti à un prototype en laboratoire et sont actuellement protégées par le secret.
Un autre projet de recherche est né à la suite de ce projet, sur la thématique de la construction additive en béton avec les équipes de R&D du groupe Vicat et le M.I.O. Ces recherches se prolongent sur un autre projet de colonisateur artificiel dans le cadre d'un projet de recherche européen H2020. Ce dernier, inspiré d'une éponge marine, est à present imprimé et sera prochainement immergé par l'Ifremer.

DÉMARCHE BIOMIMÉTIQUE

Approche
- ● Problem driven
- ● Solution based

Origine de la bio-inspiration
- ○ Opportunité/hasard/intérêt personnel
- ● Collaborations interdisciplinaires
- ● Appel à projet
- ○ Autre

Performances ciblées
- ● Confort thermique
- ● Confort visuel/lumineux
- ○ Confort acoustique
- ● Qualité de l'air intérieur
- ● Qualité de l'air extérieur
- ● Résistance aux contraintes mécaniques
- ● Gestion de l'eau intérieur
- ● Gestion de l'eau extérieur
- ● Accueil de la biodiversité
- ● Adaptation au climat
- ● Allègement de la structure
- ● Gestion des déchets
- ● Autres

Échelle d'intégration
- ● Matériaux
- ● Technologie
- ● Système façade/toit/sol
- ○ Bâtiment
- ○ Îlot
- ○ Quartier

PROCESSUS DE CONCEPTION

Utilisation d'un cadre de conception
- ● Oui
- ○ Non

Contraintes majeures
- ○ Manque de fonds
- ● Utilisation d'outils biomimétiques
- ○ Règlementations
- ○ Problèmes techniques
- ○ Autres

Complexité de la conception
- ● Haute
- ○ Faible

LIEN AVEC LA BIOLOGIE

Niveau d'inspiration
- ● Organisme
- ○ Comportement
- ● Écosystème

Dimension de l'inspiration
- ○ Forme
- ○ Matériaux
- ● Constructions/structure
- ○ Processus
- ○ Fonction

Types de modèles inspirants
- ● Animal : Scléractiniaires, *Echinoida*
- ● Plante : Posidonie, *Chlorella*
- ○ Champignon
- ○ Archea/Bacteria
- ○ Protozoaire
- ○ Chromista
- ● Écosystème : Herbiers Posidonie

Type de connaissance utilisée
- ○ Existantes pour le grand public
- ● Existantes pour le spécialiste
- ● Créées pendant la conception

Type d'apports en biologie
- ○ Historique/savoir du concepteur
- ● Acquisition lors de la conception
- ● Biologistes intégrés au processus

RÉSULTATS & INNOVATION

Complexité de la construction
- ● Haute
- ○ Faible

Niveau d'innovation
- ● Innovation de rupture
- ○ Amélioration de l'existant

LEÇONS À RETENIR

La prise en compte des problématiques issues de l'activité anthropique, pesant sur les systèmes vivants, permet à la maîtrise d'œuvre d'apporter des solutions vertueuses ayant un impact à une échelle bien plus vaste que celle du site lui-même. Ceci incite à réfléchir de façon écosystémique, à prendre en compte les déchets, l'énergie, les matériaux ou la biodiversité comme des éléments d'un système global, non comme des éléments séparés.

De cette manière, un acte local peut avoir un puissant effet global. Ce projet a été une réelle prise de conscience sur l'impact positif que peut avoir un projet si l'on dépasse le seul intérêt humain présent dans un programme classique. Dès lors, l'étude et la compréhension des principes du vivant deviennent un outil de conception puissant. Cela influe sur les outils, les méthodes de conception, la pluridisciplinarité et, *in fine*, sur la forme architecturale qui s'adapte aux multiples fonctions qu'elle remplit en interaction avec son environnement. Les limites de ce projet sont le nombre de projets de recherche associés qu'il faudrait lancer sur le long terme.

Crédits des images :

[1] Treex

[2] Tangram Architectes

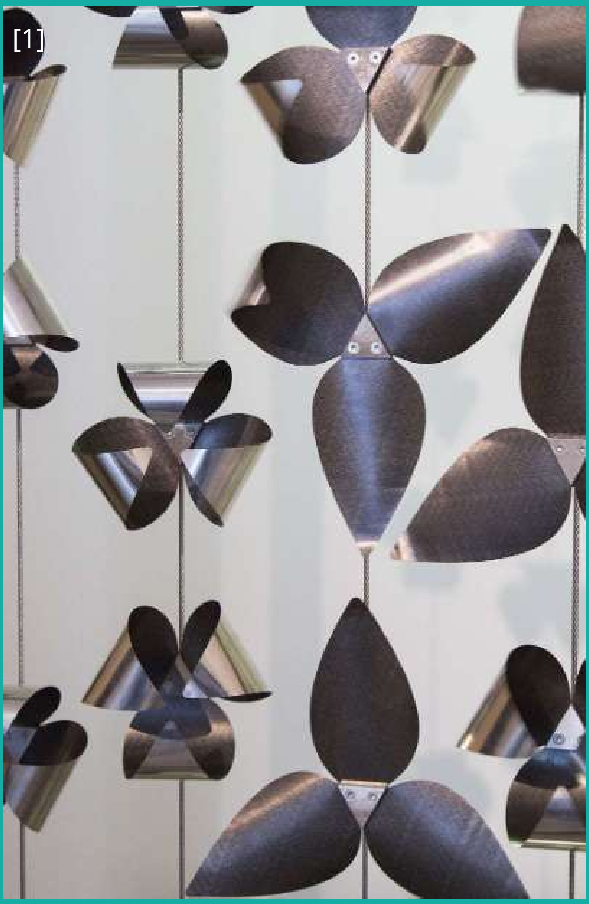

CIRC
LYON

Adresse : Biodistrict de Gerland, Lyon, France
Statut du projet : en construction
Date de livraison : 2022
Type/usage : centre de recherche
Rénovation : non
Climat : CFB - climat tempéré chaud, sans saison sèche et à été tempéré
Surface du projet : SDP 15 242 m² ; SDO 17 442 m²
Coût final du projet : 32,9 M€ HT

RÉSUMÉ

Le Centre International de Recherche sur le Cancer (CIRC) est une agence de recherche coordonnée par l'Organisation Mondiale de la Santé. Inauguré il y a 50 ans par George Pompidou, le CIRC va quitter la tour vétuste qu'il occupe actuellement dans le 8e arrondissement de Lyon pour s'installer dans le Biodistrict de Gerland. En rupture avec l'opacité habituelle des constructions de recherche, le bâtiment vise à apporter une nouvelle transparence avec son architecture bio-inspirée. Les façades ouvertes vers la ville sont en verre thermochromique et les façades donnant sur le patio sont équipées de protections solaires thermoactives en métal à mémoire de forme, à l'image des fleurs.

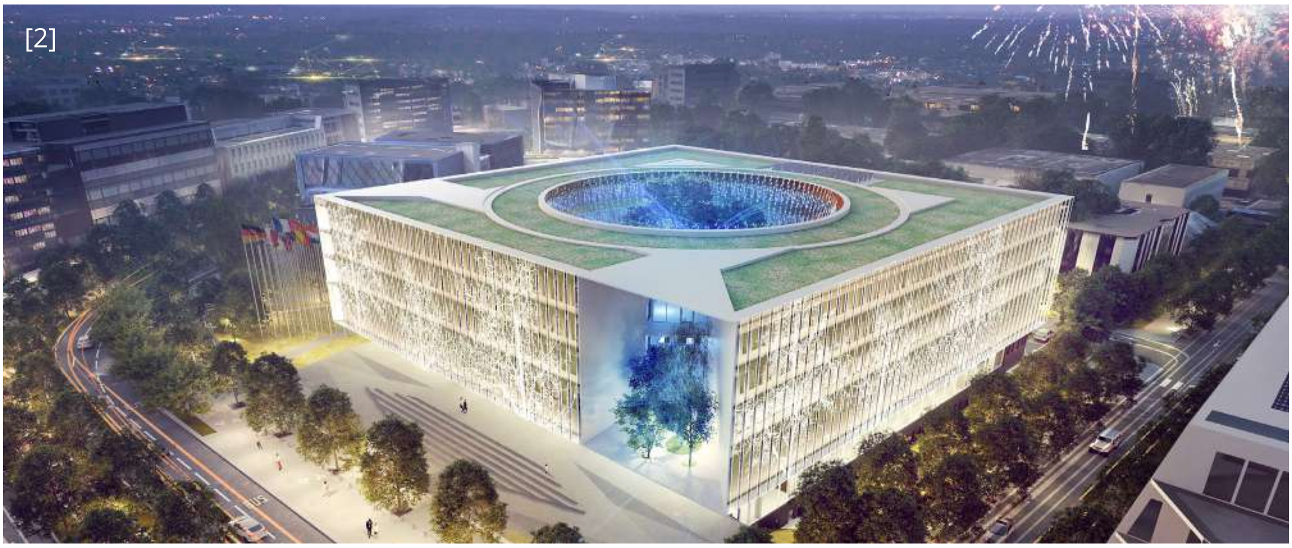

Rôle des principaux participants

Commanditaire : Métropole de Lyon
Architectes : Art&Build architectes / Unanime architectes
Mandataire entreprise générale : Demathieu Bard
BET structure et fluides : WSP France
BET environnement : Inddigo

Coûts

Coût final du projet : 32,9 M€ HT

Principaux prix

Sans objet

Certifications

• Visée certification WELL Silver

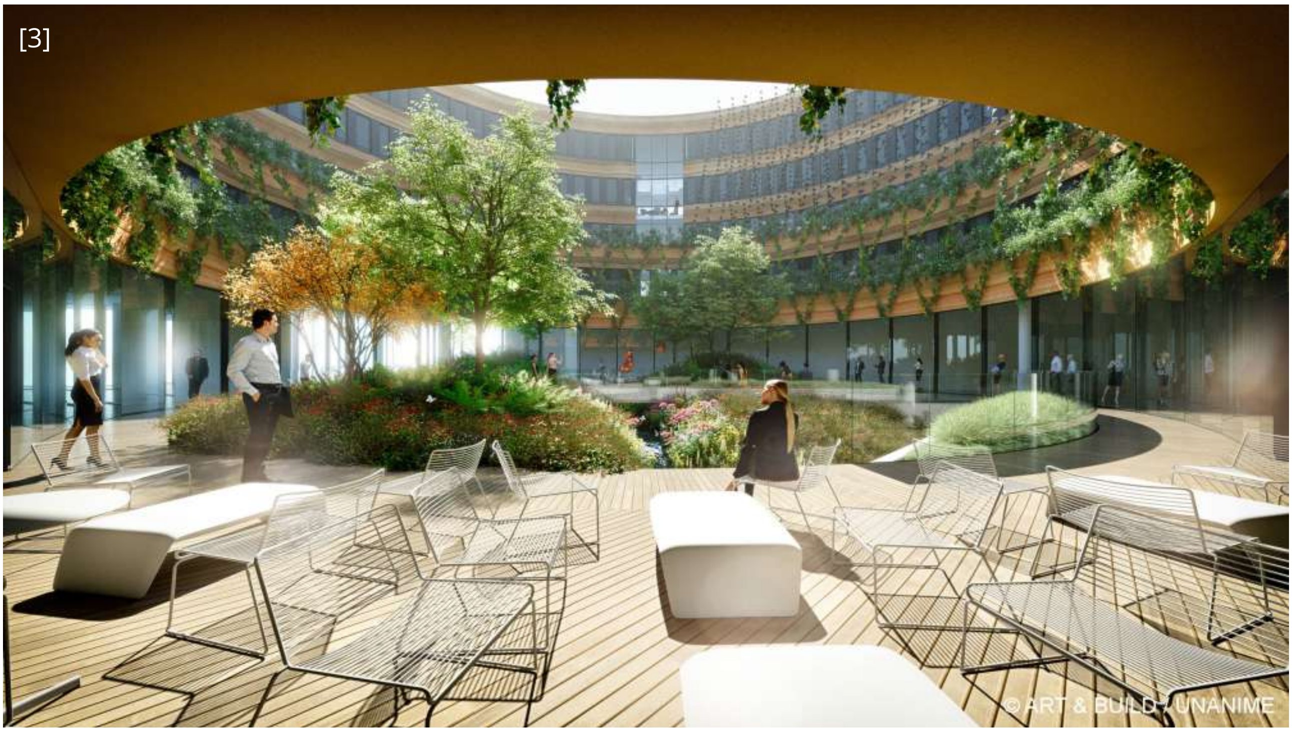

ANALYSE GLOBALE DU PROJET

Pour ce projet, la Ville de Lyon a fourni le terrain et sa dépollution (d'une valeur de 13 millions d'euros). La Métropole, la Région et l'État ont quant à eux fourni un financement partagé à hauteur de 50 millions d'euros pour le projet. Cet investissement important s'explique notamment par une forte ambition d'allier design et fonctionnalité, mais aussi sécurité, du fait de son caractère onusien. L'arrivée de l'OMS à Lyon viendra donner au CIRC son caractère international de par ses milliers de chercheurs venus du monde entier.

Objectifs

Le CIRC affirme son ambition internationale avec la construction de cet ouvrage emblématique. Le projet permet à la fois de dynamiser la recherche dans le domaine de la santé mais aussi de favoriser l'effervescence intellectuelle.
Le nouveau bâtiment a été pensé pour s'insérer dans le contexte urbain du Bio-District de Gerland. Grâce notamment à une conception bio-inspirée, il se démarque par sa performance énergétique exceptionnelle, sa flexibilité programmatique dans le court, moyen et long terme, ainsi que par sa prouesse technique avec l'intégration des innovations biomimétiques.

ANALYSE BIOMIMÉTIQUE

Conception biomimétique

Le projet tient compte des cycles de la nature et s'inspire de l'intelligence et des stratégies du vivant. L'architecture incorpore la biodiversité au cœur du patio et se différencie par sa conception bioclimatique et ses façades biomimétiques. L'édifice intègre des éléments bois, un matériau biosourcé, dans la structure des façades.

Une protection solaire biomimétique pour les façades principales

Les façades présentent deux interprétations du même objectif : l'adaptabilité à leur environnement via un stimulus externe. Dans ce projet, la température externe et l'apport calorifique du soleil déclenchent des protections solaires qui permettent de réduire la consommation d'énergie dans le bâtiment et contribuent à la maintenance du confort thermique des occupants.

• **Façades extérieures :** des lames en verre thermochromique créées une couche protectrice intégrée à la façade du bâtiment. Cette technologie s'inspire des peaux de certaines espèces qui se teignent pour se protéger des irradiations solaires lorsqu'elles deviennent trop intenses. En effet, ce dispositif change de couleur en fonction de la température de l'environnement, notamment lorsque le soleil crée un apport calorifique important en période estivale. La transmission lumineuse diminue progressivement en fonction de l'augmentation de la température atmosphérique et génère ainsi un masque solaire passif. Le système est autonome et indépendant, il ne nécessite pas d'entretien particulier. Les motifs qui apparaissent sur les façades sont inspirés des canopées d'arbre, ils apportent un effet biophilique, améliorant ainsi le bien-être des usagers du centre ainsi que les habitants du quartier. Réalisé grâce à une sérigraphie pointillée, le masque solaire est maitrisé.

• **Façades intérieures :** une protection solaire constituée de « fleurs » en métal à mémoire de forme, accrochées à une résille de câbles à l'extérieur de la façade. Le dispositif s'inspire des mouvement nastiques des plantes. Ce brise soleil dynamique et autonome rapporté à la façade ne demande ni intervention humaine ni consommation énergétique. Grâce à sa déformation passive, le système fonctionne sous l'action de l'ensoleillement qui génère une accumulation de calories. Au-delà d'une température de 25° C en surface, les lames métalliques s'ouvrent progressivement, créant ainsi un masque solaire. Quand la température de surface redescend, les « fleurs » se referment et la lumière pénètre à nouveau dans le bâtiment.

Impacts documentés du projet

Les principes fondateurs du projet sont la transparence, la convivialité, et la durabilité. Cette dernière se traduit par une conception modulable, qui permettra au bâtiment de s'adapter si besoin à l'évolution des usages et par des solutions technologiques qui améliorent la performance environnementale du projet.
L'ouvrage propose un ensemble bénéfique pour le bien-être ainsi que la santé de ses occupants et de ses visiteurs. D'une part, près de 300 chercheurs bénéficieront d'espaces dans un cadre pensé pour l'épanouissement individuel et collectif. D'autre part, la transparence du bâtiment offre aux piétons une perception inattendue de l'intérieur du bâtiment.

Impacts sur la recherche

Chaque bâtiment est un prototype car il n'y a jamais deux sites identiques. Le nouveau CIRC met en œuvre des solutions innovantes, issues de plusieurs années de recherche et développement. La fabrication des prototypes, les essais sur la performance des matériaux et les études de conformité face aux contraintes normatives, confèrent un caractère expérimental au bâtiment. Le *monitoring* de la vie du bâtiment sera capital pour les futures applications des solutions choisies.

La solution technologique de verre thermochromique retenue est issue des avancées technologiques disponibles sur le marché. Ces innovations ont fortement évolué pendant le développement du projet, depuis la phase conceptuelle en 2017 jusqu'aux études d'exécution en 2020. La solution thermochromique a été préférée à une photochromique pour éviter une activation en hiver et profiter des gains solaires pendant la période froide de l'année. Confortée par les simulations thermodynamiques, il s'agira d'une première application à grande échelle. Le *monitoring* du bâtiment permettra de prouver les données et résultats théoriques des études.

La solution technologique à mémoire de forme est issue d'un projet de recherche mené par Art&Build.
La réflexion initiale est basée sur le développement de brises soleil fixes qui se déforment de manière passive. Le projet démarre par l'identification de formes simples, puis explore différentes formes et tailles qui prennent en compte la force du vent et les déformations possibles. Le projet vise à optimiser la cinétique et la durée de vie du système. Pour ce faire, ABlab, le groupe de travail crée par Art&Build autour de ce projet de recherche, a réalisé de nombreux prototypes avant de proposer le système « Pho'liage », intégré au CIRC.

L'action du système est partiellement prise en compte dans les calculs thermiques du bâtiment. En effet, l'activation du dispositif dépend de nombreux facteurs externes tels l'ensoleillement, la température, le vent et l'orientation ; des simulations théoriques très lourdes auraient donc été nécessaires. Pour ce projet, l'effet du masque solaire est calculé selon son impact sur la régulation thermique, sans paramétrage particulier. Il s'agit d'une première application du système et son intégration est de nature expérimentale ; les résultats du monitoring permettront de calibrer les applications futures.

DÉMARCHE BIOMIMÉTIQUE

Approche
● Problem driven
○ Solution based

Origine de la bio-inspiration
○ Opportunité/hasard/intérêt personnel
● Collaborations interdisciplinaires
● Appel à projet
○ Autre

Performances ciblées
● Confort thermique
● Confort visuel/lumineux
○ Confort acoustique
○ Qualité de l'air intérieur
○ Qualité de l'air extérieur
○ Résistance aux contraintes mécaniques
○ Gestion de l'eau intérieur
○ Gestion de l'eau extérieur
○ Accueil de la biodiversité
○ Adaptation au climat
○ Allègement de la structure
○ Gestion des déchets
○ Autres

Échelle d'intégration
○ Matériaux
○ Technologie
● Système façade/toit/sol
○ Bâtiment
○ Îlot
○ Quartier

PROCESSUS DE CONCEPTION

Utilisation d'un cadre de conception
● Oui
○ Non

Contraintes majeures
○ Manque de fonds
○ Utilisation d'outils biomimétiques
○ Règlementations
○ Problèmes techniques
● Autres : intégration du système au projet

Complexité de la conception
● Haute
○ Faible

LIEN AVEC LA BIOLOGIE

Niveau d'inspiration
○ Organisme
● Comportement
○ Écosystème

Dimension de l'inspiration
○ Forme
○ Matériaux
○ Constructions/structure
● Processus
○ Fonction

Types de modèles inspirants
○ Animal
● Plante : *Trifolium*
○ Champignon
○ Archea/Bacteria
○ Protozoaire
○ Chromista
○ Écosystème

Type de connaissance utilisée
● Existantes pour le grand public
● Existantes pour le spécialiste
○ Créées pendant la conception

Type d'apports en biologie
○ Historique/savoir du concepteur
○ Acquisition lors de la conception
● Biologistes intégrés au processus

RÉSULTATS & INNOVATION

Complexité de la construction
● Haute
○ Faible

Niveau d'innovation
● Innovation de rupture
○ Amélioration de l'existant

LEÇONS À RETENIR

Un processus de recherche et développement au cœur des solutions biomimétiques : les façades bio-inspirées du projet ont été objet d'un projet de recherche, avec plusieurs prototypes et tests avant son intégration dans le projet. À la suite de cette première application et aux recherches menées pendant les études par Art&Build, des évolutions futures sont envisageables.
En ce qui concerne la solution de verre thermochromique, les températures d'activation, les degrés de transparence et les couleurs sont des variables à ajuster en fonction du contexte spécifique de chaque nouveau projet, tout comme la répartition de ces éléments sur les façades en fonction de leur contexte spécifique. Des simplifications seraient possibles, par exemple avec le rajout d'une couche supplémentaire sur le verre ou en remplaçant le verre avec d'autres matériaux transparents comme le PMMA afin de faciliter la mise en œuvre. La connaissance de données météorologiques et un approvisionnement en énergie PV pourrait permettre une activation maitrisée du système pour anticiper des périodes de fort apport calorifique.

Crédits des images :

[1] [2] [3] Art & Build Architectes / UNANIME

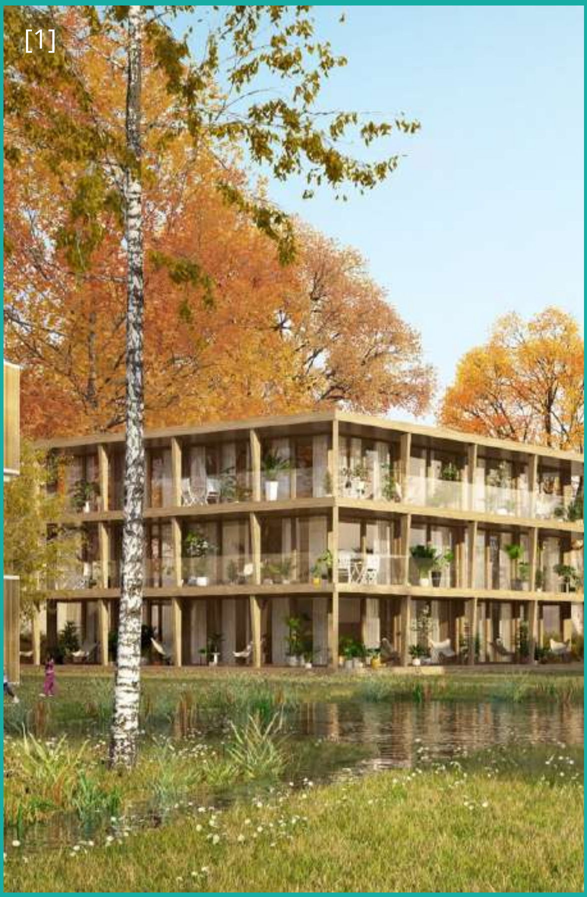

CITÉ FERTILE – TERRES DE VERSAILLES

Adresse : Versailles, France
Statut du projet : projet en cours de construction
Date de conception : 2018
Type/usage : quartier mixte à dominante résidentielle
Rénovation : oui - réhabilitation de trois bâtiments existants, deux maisons et une halle industrielle
Climat : CFB - climat tempéré chaud, sans saison sèche et à été tempéré
Surface du projet : 50 000 m² SDP
Coût de construction : -
Coût final du projet : -

Membres du Biomim'City Lab engagés :

ICADE

RÉSUMÉ

La Ville de Versailles et l'aménageur SNC Versailles PION (Icade) pilotent le nouveau quartier « Terres de Versailles » qui sera réalisé entre le domaine du Château de Versailles et Saint-Cyr l'École jusqu'en 2026.
Cette ancienne emprise militaire de 20 hectares préservera un cadre patrimonial exceptionnel grâce à un projet urbain aéré, végétalisé, de faible hauteur et contemporain, dans la tradition de la cité-jardin, ou cité fertile.
LA SNC Versailles PION s'est engagée à réaliser cette opération en respectant le paysage en lien avec le domaine du château, en ouvrant les logements sur la nature, en protégeant des nuisances sonores et en maîtrisant la place de la voiture.
Créer le 9e quartier de Versailles implique de développer une gamme complète de logements afin de donner au projet toute la diversité et la qualité d'un tissu urbain. Le projet comporte ainsi 4 typologies d'habitat : la maison de ville, la maison jumelée, l'individuel en duplex et le petit collectif qui comporteront des espaces extérieurs privés généreux, des ouvertures sur le paysage et un accès aux jardins partagés.
Dans une démarche alliant innovation, éducation et ambition environnementale sont aussi prévus des commerces et activités (alimentaire bio, restaurant, ferme...), des lieux d'apprentissage traditionnels (crèche et école) et novateurs (écocentre...), ainsi qu'un équipement hôtelier/loisirs, et une prairie fauchée paysagère. C'est au total 50 000 m² de surface de plancher qui seront réalisés, dont 550 logements.

Rôle des principaux participants

Maîtrise d'ouvrage : Ville de Versailles / SNC Versailles PION (Icade)
Maitrise d'œuvre des espaces publics : Lambert Lenack Architecture et Michel Desvigne Paysagiste
AMO Nature-Art-Éducation : Deyrolle
BET VRD : Oteis
BET gestion des eaux pluviales : Phytorestore
AMO Développement Durable : Une Autre Ville
AMO Biodiversité : CDC Biodiversité
AMO environnement et qualité des sols : Sol Paysage
AMO site et sols pollués : Cèdres

Coûts

Coût de construction : -
Coût final du projet : -

Principaux prix

Sans objet

Certifications

Visées :
- Label Nature-Art-Education
- Label E+C- pour les constructions
- Bâtiment passif pour le groupe scolaire

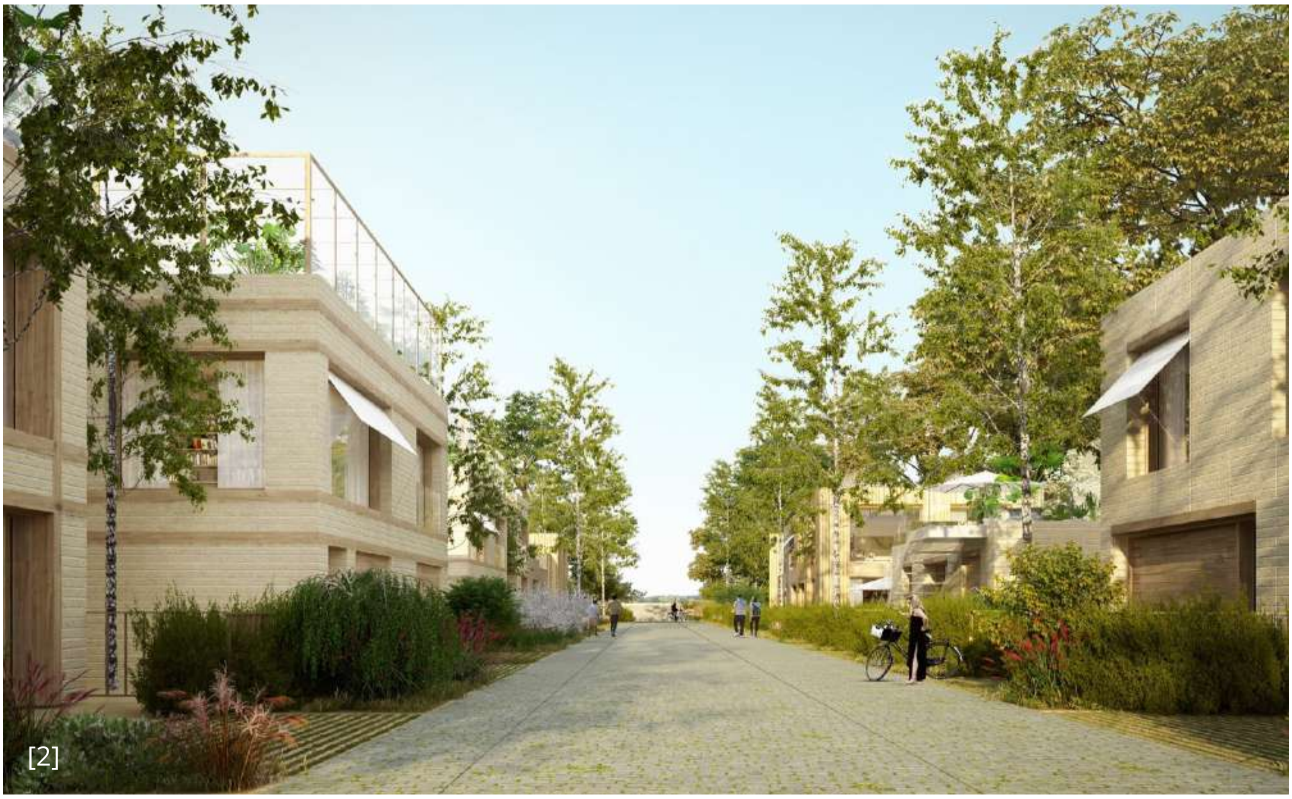
[2]

ANALYSE GLOBALE DU PROJET

Entre l'extrémité nord-ouest du domaine national du Château de Versailles et Saint-Cyr l'École, le grand projet urbain de reconversion de l'ancien site de la caserne de Pion « Terres de Versailles », fera de ces vingt hectares un quartier contemporain, aéré et végétalisé de faible hauteur. Intégré à la structure paysagère du parc du château et invisible depuis le site classé, il cultive l'esprit des cités jardins. Pour rendre à ce territoire sa vocation du XVIIe siècle en réinventant un nouvel équilibre entre la ville et la nature, ce projet d'aménagement ambitionne de devenir une référence internationale dans la pensée de la ville. La modification du plan local d'urbanisme par la Ville de Versailles en 2016 a ainsi permis de réserver le terrain en zone agricole. La SNC Versailles PION a été désignée lauréate du concours lancé par la Ville de Versailles en mai 2018.

Le projet urbain est basé sur un programme majoritairement résidentiel, complété par un programme de commerces, services et équipements publics. La conservation des bâtiments existants permettra de garder la mémoire du patrimoine bâti.

• Programmes résidentiels : 42160 m^2
• Équipement hôtelier : 5000 m^2
• Commerces (superette, restaurant, boulangerie) : 850 m^2
• Meulières : 310 m^2
• Équipements publics :
  o Groupe scolaire : 1370 m^2
  o Salle polyvalente : 400 m^2
• Équipement privé :
  o Crèche : 400 m^2

La durée contractuelle de la concession d'aménagement a été fixée à 10 ans.

Objectifs

Comment construire 550 logements, tout en laissant toute sa place à une nature généreuse, accessible et productive ? Ainsi, les espaces extérieurs seront très dessinés, un peu à l'image de jardins à la française et organisés autour de « pièces » : une place boisée, une grande terrasse qui longe le parc du Château, une « prairie des sports » et une prairie à moutons, toutes aménagées par Icade et rétrocédées à la ville. Le modèle du verger a été étudié et la cueillette spontanée du public sera rendue possible (branches accessibles et déchargées par les passants, nettoyage des fruits écrasés sur le sol...).

Les objectifs partagés par Icade et la Ville de Versailles pour ce projet sont :

• La **mise en valeur des perspectives et du patrimoine ;**
• La mise en œuvre d'un **paysage nourricier** ;
• L'**innovation et l'excellence environnementale** (label E+C- pour l'ensemble des constructions).

Revenir à la liste des projets

ANALYSE BIOMIMÉTIQUE

Conception biomimétique

Terres de Versailles a été conçu pour réaliser une cité jardin nouvelle génération, une Cité fertile, dans laquelle les habitants vivent au plus près de la Nature. Dans cette perspective, le projet prévoit l'implantation d'une micro-ferme, des jardins partagés pour les habitants, des vergers.
La philosophie du projet repose sur le tryptique Nature-Art-Éducation pensé par Deyrolle et qui deviendra bientôt un Label. Terres de Versailles sera le premier projet à obtenir ce label Nature-Art-Éducation.
Cette Cité se doit de répondre aux enjeux environnementaux et sociétaux où l'économie s'inspire de l'écologie et du fonctionnement des écosystèmes (avec un approche biomimétique) pour rendre les citoyens plus heureux, plus solidaires des autres et de la terre et plus préparés à la transmission aux générations futures.

La Cité Idéale du XXIe siècle devra être une cité nourricière. C'est le seul modèle qui nous permettra de préserver nos identités, de reconnecter sa population au bien-être, à la santé, au Sens et à la Nature, ainsi les élements suivants du projet méritent d'être à l'honneur :

• La cité fertile sera un **Jardin nourricier** ;

• Les **habitants** seront conscients de leur rôle et **acteurs de leur territoire** ;

• L'**innovation sera interconnectée** par le prisme Nature–Art–Éducation ;

• L'**architecture signifiante** sera en harmonie avec les valeurs de « Terres de Versailles » ;

• L'ambition sera de construire un **lieu exemplaire adapté aux enjeux contemporains** ;

• La desserte énergétique sera issue de la **géothermie**.

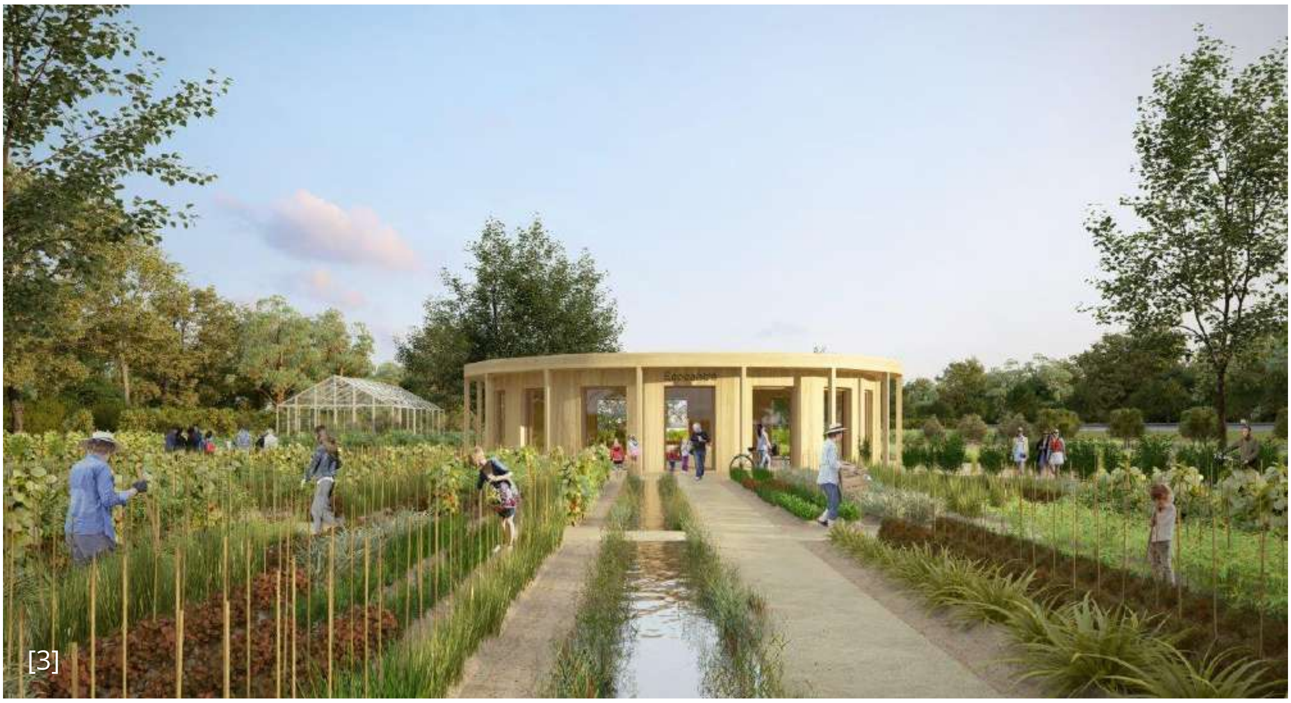
[3]

Revenir à la liste des projets

DÉMARCHE BIOMIMÉTIQUE

Approche
- [x] Problem driven
- [ ] Solution based

Origine de la bio-inspiration
- [ ] Opportunité/hasard/intérêt personnel
- [x] Collaborations interdisciplinaires
- [x] Appel à projet
- [ ] Autre

Performances ciblées
- [x] Confort thermique
- [x] Confort visuel/lumineux
- [ ] Confort acoustique
- [x] Qualité de l’air intérieur
- [x] Qualité de l’air extérieur
- [ ] Résistance aux contraintes mécaniques
- [ ] Gestion de l’eau intérieur
- [x] Gestion de l’eau extérieur
- [x] Accueil de la biodiversité
- [x] Adaptation au climat
- [ ] Allègement de la structure
- [x] Gestion des déchets
- [ ] Autres

Échelle d'intégration
- [x] Matériaux
- [ ] Technologie
- [ ] Système façade/toit/sol
- [ ] Bâtiment
- [ ] Îlot
- [x] Quartier

PROCESSUS DE CONCEPTION

Utilisation d'un cadre de conception
- [x] Oui
- [ ] Non

Contraintes majeures
- [ ] Manque de fonds
- [ ] Utilisation d'outils biomimétiques
- [ ] Règlementations
- [x] Problèmes techniques
- [ ] Autres

Complexité de la conception
- [ ] Haute
- [x] Faible

LIEN AVEC LA BIOLOGIE

Niveau d'inspiration
- [ ] Organisme
- [ ] Comportement
- [x] Écosystème

Dimension de l'inspiration
- [x] Forme
- [ ] Matériaux
- [ ] Constructions/structure
- [ ] Processus
- [x] Fonction

Types de modèles inspirants
- [ ] Animal
- [ ] Plante
- [ ] Champignon
- [ ] Archea/Bacteria
- [ ] Protozoaire
- [ ] Chromista
- [x] Écosystème

Type de connaissance utilisée
- [x] Existantes pour le grand public
- [ ] Existantes pour le spécialiste
- [x] Créées pendant la conception

Type d'apports en biologie
- [x] Historique/savoir du concepteur
- [ ] Acquisition lors de la conception
- [ ] Biologistes intégrés au processus

RÉSULTATS & INNOVATION

Complexité de la construction
- [ ] Haute
- [x] Faible

Niveau d'innovation
- [ ] Innovation de rupture
- [x] Amélioration de l'existant

Revenir à la liste des projets

LEÇONS À RETENIR

Le projet Terres de Versailles a permis ICADE et l'équipe de conception de pousser les notions d'intégration entre ville et nature avec son projet de cité fertile, avec une approche biomimétique à l'échelle des écosystèmes.
L'intégration des acteurs engagés dans la transition écologique et le label Nature-Art-Education ont été des élements clés dans le succès du projet et dans sa performance écologique.

Crédits des images :

[1] [2] [3] ICADE

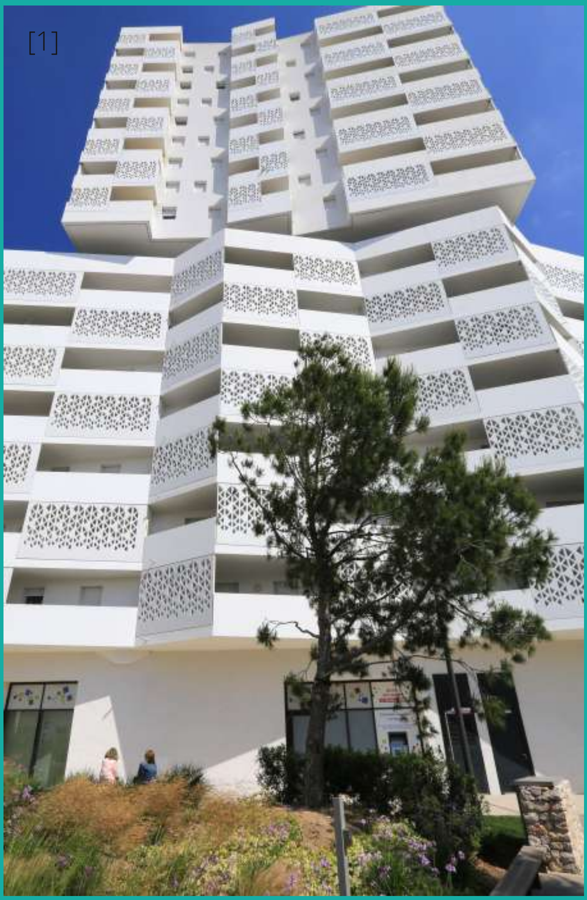
[1]

ÉCOQUARTIER SMARTSEILLE

Adresse : Îlot Allar, 1 Rue du Devoir, 13015 Marseille
Statut du projet : en construction (80% déjà livrés)
Date de conception : 2013
Type/usage : mixte
Rénovation : non
Climat : CSA - climat tempéré chaud à été chaud et sec
Surface du projet : 58 000 m² SDP
Coût de construction : 85 000 000 € HT
Coût final du projet : 160 000 000 € HT (investissement)

Membres du Biomim'City Lab engagés :

EIFFAGE
Ceebios

RÉSUMÉ

Situé au cœur de l'ÉcoCité Euroméditerranée 2 à Marseille, l'écoquartier Smartseille est un projet de 2,7 hectares porté par Eiffage Immobilier. Le site accueille un programme axé sur la mixité fonctionnelle, sociale et générationnelle : logements sociaux et en accession, bureaux, hôtel, résidence intergénérationnelle Cocoon'Ages, crèche, école, commerces et services de proximité, conciergerie de quartier et espace public central végétalisé. À terme, plus de 4 000 personnes vont vivre, travailler et habiter à Smartseille.
Première application du laboratoire en développement urbain durable Phosphore créé par Eiffage en 2007, ce projet vise à devenir un démonstrateur de la ville durable méditerranéenne avec une prise en compte globale et systémique du développement durable. Le projet se distingue également par la richesse de l'écosystème partenarial entourant la ville de Marseille, l'opérateur d'aménagement Euroméditerranée et Eiffage. Smartseille propose ainsi de nombreuses solutions adaptées aux besoins des habitants et des usagers et répondant aux enjeux de la transition écologique et du mieux vivre ensemble. Parmi ces innovations, on peut notamment citer les parkings mutualisés, l'accueil des véhicules en autopartage, la boucle à eau de mer, la le coaching et la solidarité énergétique, la conciergerie de quartier et le portail numérique associé ou encore l'agriculture urbaine. Le site a également fait l'objet d'une expérimentation de dépollution douce par mycoremédiation.

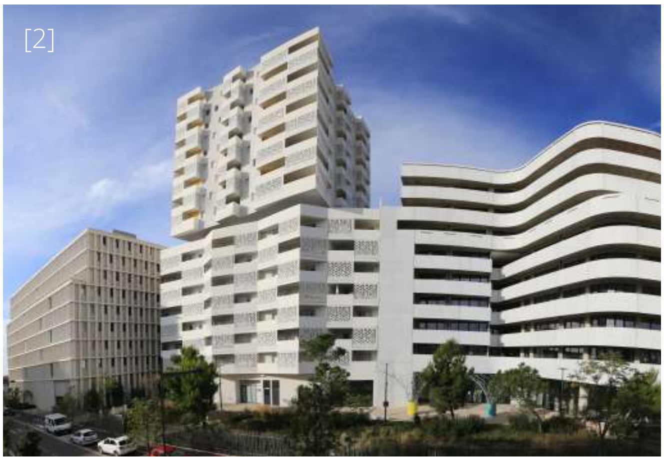
[2]

Revenir à la liste des projets

Rôle des principaux participants

Commanditaire : Ville de Marseille et l'Établissement Public d'Aménagement Euroméditerranée (EPAEM)
Porteur du projet : Eiffage Immobilier
Entreprise générale : Eiffage Construction
Maîtrise d'œuvre de l'EPAEM pour la définition urbaine : Agence Leclercq Associés ; Jacques Sbriglio ; Rémy Marciano ; Agence Ter
Pilotage de la partie aménagement de l'écoquartier : Agence d'urbanisme Babylone Avenue
Projet paysager : Agence Jean Mus
Conception architecturale :
• Îlot A (bureaux et hôtel) : Eduardo Souto de Moura et Jacques Sbriglio
• Îlot B (bureaux et logements) : Carta Associés et Atelier EGR
• Îlot C (conciergerie, logements, bureaux, commerces) : Atelier 82 et Corinne Vezzoni
• Îlot D (résidence pour personnes âgées, crèche, logements, commerces, bureaux) : Mathoulin-Jardin Architectes associés
• Îlot E (logements, bureaux, commerces) : Jean-Michel Battesti
Dépollution : Ortec, Polypop, Ceebios

Coûts

Coût de construction : 85 000 000 € HT
Coût final du projet : 160 000 000 € HT (investissement)

Principaux prix

2009 • ÉcoCité - Smartseille est l'un des 3 projets structurants ayant permis l'obtention du label ÉcoCité à l'extension d'Euroméditerranée en 2009

2015 • Smartseille fait partie de la première génération des « Démonstrateurs industriels pour la ville durable »

Certifications

• Logements certifiés NF Habitat
• Logements labellisés BDM (Bâtiments Durables Méditerranéens)
• Bureaux labellisés BDM
• Bureaux îlot A labellisés Effinergie+
• Bureaux îlot C labéllisés NF HQE
• Label BiodiverCity

ANALYSE GLOBALE DU PROJET

L'îlot démonstrateur Allar – alias Smartseille – est le premier jalon de l'extension du périmètre d'Euroméditerranée (Euromed 2). Le projet, s'inscrivant dans une opération plus globale de 170 hectares, vise à donner naissance à une ÉcoCité exemplaire et ainsi poser l'une des briques marseillaises de la ville de demain. Accueillant autrefois une usine de production de gaz, l'îlot Allar se situe au coeur des quartiers Nord, en bordure d'un QPV (Quartier Prioritaire de la Ville). La population vivant dans le quartier attenant, est en quête d'un renouveau. L'opération se doit d'assurer un développement urbain équilibré en améliorant significativement les conditions résidentielles des populations.

Le projet vise ainsi à donner naissance au 112e village de Marseille avec un programme axé sur la mixité fonctionnelle, sociale et générationnelle : logements sociaux et en accession, bureaux, hôtel, résidence intergénérationnelle Cocoon'Ages, crèche, école, commerces et services de proximité, conciergerie de quartier et un espace public central végétalisé. Au total, 58 000 m² SDP seront réalisés sur le site de 2,7 ha avec une densité de 148 logements par hectare.

Objectifs

Conçu dans le respect du génie du territoire, Smartseille se veut être le démonstrateur opérationnel d'une approche systémique de la ville durable méditerranéenne : efficience énergétique, construction labellisée Bâtiment Durable Méditerranéen, mobilités décarbonées, qualité d'usage, nature en ville. Le quartier est un concentré d'innovations au service des habitants et des usages, et touchant l'ensemble des composantes de l'écosystème urbain :

• **Boucle de thalassothermie** (boucle à eau de mer) réalisée par Dalkia Smart Building et baptisée Massiléo, inaugurée en 2016, portant à plus de 70 % la couverture des besoins thermiques (chaud et froid) du quartier par les énergies renouvelables ;

• **Solidarité Energétique** entre les bâtiments: la climatisation des bureaux fabrique de l'ECS pour les logements mitoyens ;

• **Architecture bioclimatique** ;

• **Expérimentation de la dépollution alternative par les champignons** selon le principe de la mycoremédiation ;

• **Logements évolutifs, studio partagé et résidence intergénérationnelle** ;

• **Nouveaux services de proximité :** auto-partage électrique, parking mutualisé, coaching énergétique, conciergerie de quartier ;

• **Services numériques :** habitat intelligent, réseau social de quartier, etc. ;

• **Points d'apport volontaire des déchets enterrés** ;

• **Biodiversité méditerranéenne**, agriculture urbaine et installation de 3 corolles végétalisées Urban Canopee.

Conception biomimétique

L'approche biomimétique dans le projet s'est retrouvée à travers la dépollution des sols et l'expérimentation de la mycoremédiation. Sur le site Allar, auparavant occupé par une usine de production de gaz, cette technique a pu être testée à grande échelle en complément d'une dépollution par des méthodes traditionnelles. Grâce à Ceebios, Eiffage a pu rencontrer la start-up Polypop, spécialisée dans cette technique de dépollution douce par le mycélium (partie végétative du champignon). La mycoremédiation permet l'absorption et la transformation des contaminants du sol (hydrocarbures lourds et aromatiques, cadmium et plomb notamment).

La méthode présente plusieurs avantages, comme une consommation d'énergie moins importante que les techniques traditionnelles, une réduction des coûts liés à l'enfouissement des terres polluées de type 1, le réemploi des terres sur le site en évitant l'émission de GES liés à leur évacuation. L'atout de cette démarche biomimétique réside donc dans le bénéfice environnemental global obtenu. Entre 2013 et 2015, les équipes du groupe Eiffage ont ainsi pu expérimenter ce procédé.

Après des études préalables ayant permis de sélectionner les souches de mycélium adaptées aux polluants du site, des tests avaient été réalisés avec succès en laboratoire. En effet, en 25 jours, on avait observé que 47% des hydrocarbures lourds et 35% des hydrocarbures aromatiques avaient été détruits et que 55% du cadmium et 40% du plomb avaient été retirés. L'expérience avait ensuite été reproduite à plus grande échelle sur une petite parcelle du terrain sur deux (myco)pilles de 90m^3 au total, une première en Europe. Celles-ci étaient alors composées d'un mille-feuille formé d'une couche de terre polluée et d'une couche de substrat à base de pailles inoculées du mycélium.

Une bonne partie des contaminants avait finalement été détruite, mais les seuils attendus n'avaient malheureusement pas été atteints. La notion de protection du vent entraînant un dessèchement prématuré des champignons a ainsi été mise en évidence avec la nécessité de revoir le système de bâche et d'irrigation. D'autres expérimentations de ce type sont encore nécessaires pour faire avancer la technique, et il est important de préciser qu'il faut disposer de temps et d'espace sur le site pour pouvoir mettre en oeuvre cette technique.

DÉMARCHE BIOMIMÉTIQUE

Approche
- [ ] Problem driven
- [x] Solution based

Origine de la bio-inspiration
- [x] Opportunité/hasard/intérêt personnel
- [ ] Collaborations interdisciplinaires
- [ ] Appel à projet
- [ ] Autre

Performances ciblées
- [x] Confort thermique
- [ ] Confort visuel/lumineux
- [ ] Confort acoustique
- [ ] Qualité de l'air intérieur
- [ ] Qualité de l'air extérieur
- [ ] Résistance aux contraintes mécaniques
- [ ] Gestion de l'eau intérieur
- [ ] Gestion de l'eau extérieur
- [x] Accueil de la biodiversité
- [x] Adaptation au climat
- [ ] Allègement de la structure
- [ ] Gestion des déchets
- [x] Autres : dépollution

Échelle d'intégration
- [ ] Matériaux
- [x] Technologie
- [ ] Système façade/toit/sol
- [ ] Bâtiment
- [x] Îlot
- [ ] Quartier

PROCESSUS DE CONCEPTION

Utilisation d'un cadre de conception
- [x] Oui
- [ ] Non

Contraintes majeures
- [ ] Manque de fonds
- [ ] Utilisation d'outils biomimétiques
- [ ] Règlementations
- [x] Problèmes techniques
- [ ] Autres

Complexité de la conception
- [x] Haute
- [ ] Faible

LIEN AVEC LA BIOLOGIE

Niveau d'inspiration
- [x] Organisme
- [ ] Comportement
- [ ] Écosystème

Dimension de l'inspiration
- [ ] Forme
- [ ] Matériaux
- [ ] Constructions/structure
- [ ] Processus
- [x] Fonction

Types de modèles inspirants
- [ ] Animal
- [ ] Plante
- [x] Champignon
- [ ] Archea/Bacteria
- [ ] Protozoaire
- [ ] Chromista
- [ ] Écosystème

Type de connaissance utilisée
- [ ] Existantes pour le grand public
- [x] Existantes pour le spécialiste
- [ ] Créées pendant la conception

Type d'apports en biologie
- [x] Historique/savoir du concepteur
- [x] Acquisition lors de la conception
- [ ] Biologistes intégrés au processus

RÉSULTATS & INNOVATION

Complexité de la construction
- [x] Haute
- [ ] Faible

Niveau d'innovation
- [x] Innovation de rupture
- [ ] Amélioration de l'existant

Revenir à la liste des projets

LEÇONS À RETENIR

Ce projet participe à la reconversion d'un ancien site industriel en un écoquartier en mettant notamment en œuvre des techniques de dépollution des sols par mycorémédiation et en y implémentant une dynamique sociale. Ce projet invite Eiffage à innover en termes d'urbanisme, d'éco-conception, de mobilité, d'habitat et d'aménagement des espaces créés, afin de participer à la construction de la ville durable de demain.

Crédits des images :

[1] [2] Action Photo Video Thierry Lavernos

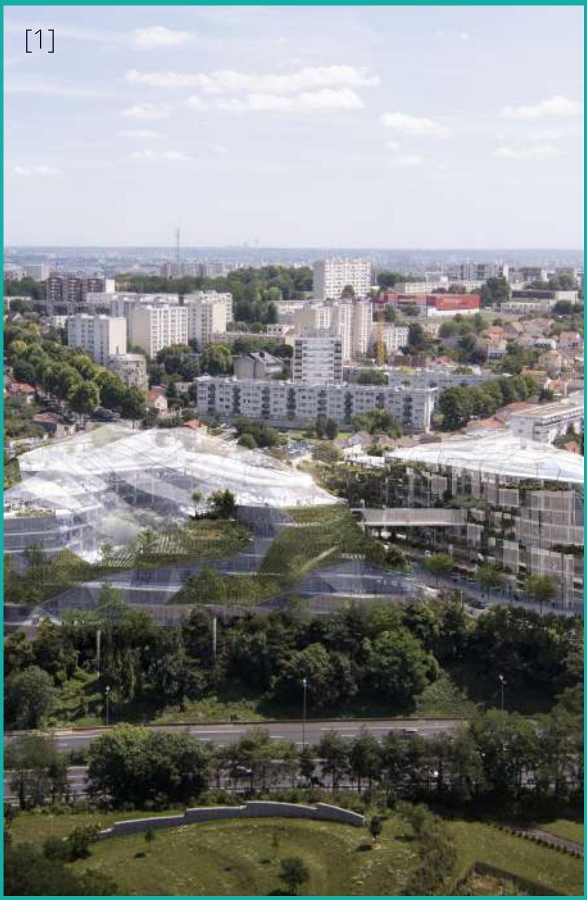

ÉCOTONE

Adresse : Arcueil, France
Statut du projet : projet lauréat
Date de conception : 2017
Type/usage : tertiaire mixte
Rénovation : non
Climat : CFB - climat tempéré chaud, sans saison sèche et à été tempéré
Surface du projet : 82 000 m²
Coût de construction : 2.500 €/m²
Coût final du projet : 300 M€ HT

Membres du Biomim'City Lab engagés :

ELAN Ceebios

RÉSUMÉ

Situé entre une zone urbaine et un parc, Écotone est une zone de transition entre deux écosystèmes créant un immobilier enrichi en biodiversité, telle la définition de son éponyme. Le bâtiment est un prolongement du paysage devenant un paysage habité et vivant, rythmé par les saisons. Les architectes ont imaginé une double peau biomimétique entre verre, ETFE et végétalisation afin de protéger les usagers des nuisances extérieures (bruit et chaleur), de proposer une luminosité naturelle ainsi qu'une visibilité sur le paysage. L'épaisseur de l'enveloppe permettra d'offrir de nouveaux usages et espaces de vie situés dans ces zones de transition.
Écotone va être un actif évolutif unique avec une enveloppe conçue comme un épiderme vivant et protecteur. Écotone montre ce que le biomimétisme peut apporter à l'architecture (en termes d'efficacité énergétique, de qualité esthétique, de pérennité, de qualité d'usage, etc.) et la façon dont l'application d'une démarche biomimétique bouleverse le déroulement habituel d'un projet urbain (implication de scientifiques très tôt dans le travail de réflexion, renouvellement de la manière de penser des questions telles que celles liées aux usages, structures, matériaux, etc.). Le comité scientifique de biodiversité et de biomimétisme, constitué de Ceebios, du MNHN, de Elan et du CRIGEN, a été créé spécialement pour intégrer les connaissances scientifiques de ces sujets en amont du projet. L'humain est au cœur du projet grâce à la mise en place d'un pôle de santé.
Le projet contribue à la santé des riverains en offrant des équipements sportifs. Le projet favorise également le lien intergénérationnel, avec une crèche et une résidence de chercheurs et jeunes actifs.

Rôle des principaux participants

Maîtrise d'ouvrage : Compagnie de Phalsbourg (mandataire), Engie Avenue, Hertel Investissement et Codeurs & Compagnie
Architectes : Triptyque Architecture, Duncan Lewis Scape Architecture, Parc Architectes et Oxo Architectes
Paysagiste : Atelier Georges
Conseil environnemental : Elan
Comité scientifique biomimétisme & biodiversité : Ceebios, Muséum National d'Histoire Naturelle, Elan, CRIGEN

Coûts

Coût de construction : 2.500€/m^2
Coût final du projet : 300 M€ HT

Principaux prix

Sans objet

Certifications

Visées :
• Breeam 2016, niveau Very Good et HQE 2016 Très performant pour l'évaluation des performances environnementales du bâtiment
• Cradle to Cradle, pour mettre en exergue les démarches d'économie circulaire en lien avec l'approche écosystémique du biomimétisme
• Biodivercity, pour valoriser l'intégration de la biodiversité au sein de l'immobilier
• Démarche E+C- démontrant le fort engagement pour la production d'énergies renouvelables, la réduction de la consommation énergétique, ainsi que pour l'inscription dans une démarche bas carbone
• L'utilisation de matériaux biosourcés sera également valorisée grâce à l'atteinte du niveau 2 du label « Matériaux biosourcés »

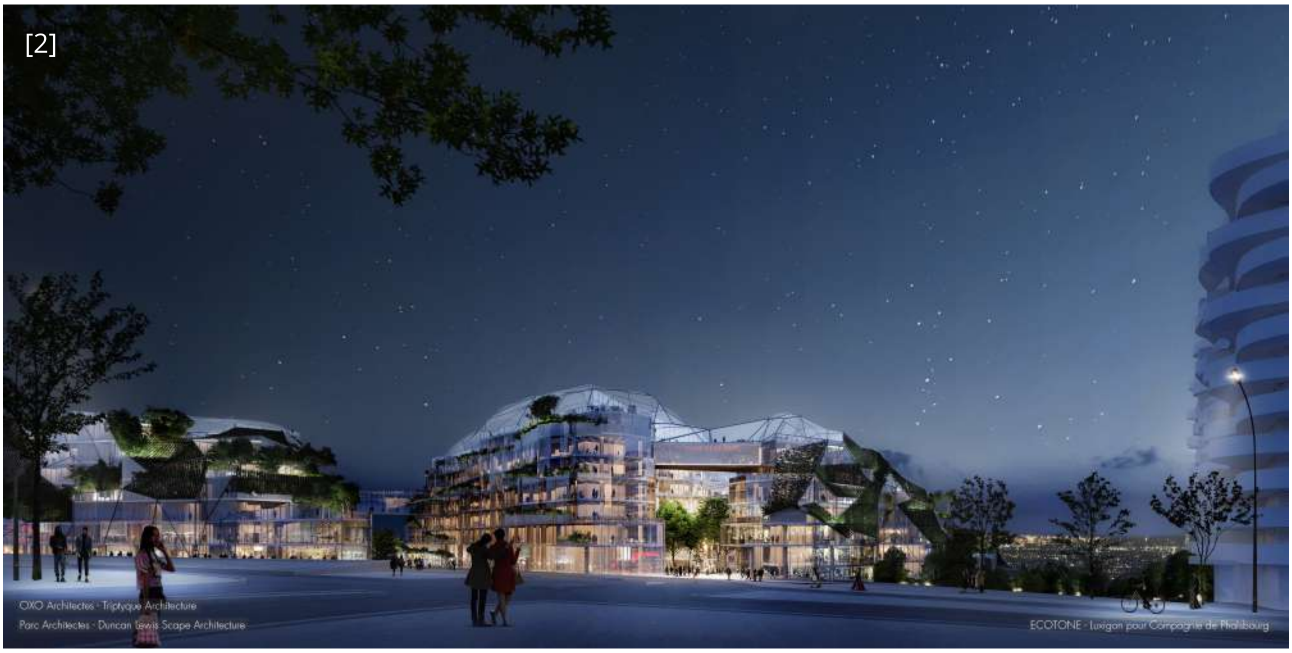

ANALYSE GLOBALE DU PROJET

Écotone est lauréat du concours Inventons la Métropole pour le site d'Arcueil. Il s'agit d'un des premiers projets immobiliers de cette envergure à se revendiquer biomimétique en 2017.
Les opérateurs immobiliers : Engie Avenue, Hertel Investissement et Codeurs & Compagnie ont constitué avec le mandataire la Compagnie de Phalsbourg une équipe pluridisciplinaire regroupant plus de 30 partenaires (architectes, comité scientifique, paysagiste, bureaux d'études techniques, conseil et start-ups innovantes).
Bâtir un immeuble jouant ce rôle d'interface entre les deux milieux de ce site privilégié d'Arcueil, que sont la ville et la nature, est l'ambition d'Écotone. Ce projet amorce un changement de paradigme dans nos modes de vie, de production et de consommation en reconnaissant que nous serons toujours dépendants de la nature, qu'il faut l'accepter et en faire un atout, y trouver une force. C'est pourquoi le projet Écotone, en tant que coteau amplifié et paysage habité, s'inspire de et fera corps avec la nature.

Objectifs

Les objectifs du projet sont :

• D'apporter un **dynamisme économique complémentaire** au site avec une programmation mixte.

• De **créer une jonction** sur ce site situé à un point stratégique entre les **trois villes** suivantes : Arcueil, le Kremlin-Bicêtre, Gentilly.

• De **s'inspirer du vivant** et de réintroduire de la **biodiversité en ville**.

• D'être un bâtiment de référence en **biomimétisme**.

Conception biomimétique

L'approche biomimétique se retrouve à différentes échelles du projet.

Dimension organisationnelle

La création d'une équipe pluridisciplinaire, possible grâce à la création d'un comité scientifique en biodiversité et biomimétisme constitué de Ceebios, MNHN, Elan et CRIGEN.

Dimensions architecturale et programmatique

Écotone va fonctionner comme un écosystème programmatique ouvert sur son environnement. Les espaces sont accessibles aux habitants et riverains pour répondre aux besoins du quartier. Contrairement à un projet lambda, les 4 architectes ont travaillé ensemble sur un seul et même bâtiment en répondant à un cahier des charges commun, pour créer des interactions entre les fonctions à l'image des relations qui existent dans un écosystème naturel.

Dimensions expérimentation et technologie

L'approche technologique qui permet de s'inspirer du vivant pour reproduire un processus est également expérimentée dans le projet Écotone.

• La **paroi Hygroskin**, développée par l'institut de Stuttgart, est inspirée de la pomme de pin. Elle se compose d'alvéoles qui s'ouvrent et se ferment naturellement en fonction du taux d'humidité dans l'air, grâce aux propriétés du bois qui les constitue. En plus des conditions de confort qu'elle offre aux usagers, cette expérimentation aura un rôle pédagogique et de sensibilisation au potentiel du biomimétisme.

• Écotone souhaite miser sur la pompe innovante bio-inspirée de l'entreprise française **Wavera** pour réduire la consommation énergétique. Cette pompe imite le mouvement des nageoires des dauphins pour une réduction de 30% de sa consommation en électricité.

• Des parcours biomimétiques sont conçus de sorte à multiplier les sollicitations des sens et rythmer les promenades, pour créer des rez-de-chaussée actifs. **Innoside** s'inspire de l'intelligence collective des fourmis pour développer une méthode de prédiction de mobilité et optimiser les flux.

• **Novobiom** propose un service innovant et compétitif de traitement biologique des sols. Motivé par la promotion de technologies éco-responsables bio-inspirées permettant de participer à la restauration d'écosystèmes dégradés, Novobiom base sa technologie sur l'emploi de champignons qui seront capables d'enrichir considérablement les sols dans l'optique de fertiliser les substrats utilisés dans les espaces végétalisés du projet.

Dimension écosystémique

Dans une logique de continuité et d'intégration au site, Ecotone évalue ses services écosystémiques, les services rendus par le bâtiment à son environnement. Ce sont les impacts positifs créés par le projet selon plusieurs axes structurants : L'amélioration de la biodiversité ; La production et la gestion des flux de matières avec l'utilisation de matériaux vertueux ; La réduction des émissions carbone; La production et la gestion d'énergie renouvelable ; La gestion des eaux pluviales ; La qualité de vie et la sensibilisation à l'approche environnementale dans la construction; L'adaptation au changement climatique.

DÉMARCHE BIOMIMÉTIQUE

Approche
● Problem driven
○ Solution based

Origine de la bio-inspiration
● Opportunité/hasard/intérêt personnel
● Collaborations interdisciplinaires
○ Appel à projet
○ Autre

Performances ciblées
● Confort thermique
● Confort visuel/lumineux
● Confort acoustique
● Qualité de l’air intérieur
○ Qualité de l’air extérieur
○ Résistance aux contraintes mécaniques
○ Gestion de l’eau intérieur
● Gestion de l’eau extérieur
● Accueil de la biodiversité
● Adaptation au climat
○ Allègement de la structure
● Gestion des déchets
○ Autres

Échelle d'intégration
○ Matériaux
○ Technologie
● Système façade/toit/sol
● Bâtiment
● Îlot
● Quartier

PROCESSUS DE CONCEPTION

Utilisation d'un cadre de conception
● Oui
○ Non

Contraintes majeures
○ Manque de fonds
● Utilisation d'outils biomimétiques
● Règlementations
● Problèmes techniques
○ Autres

Complexité de la conception
● Haute
○ Faible

LIEN AVEC LA BIOLOGIE

Niveau d'inspiration
● Organisme
○ Comportement
● Écosystème

Dimension de l'inspiration
● Forme
○ Matériaux
○ Constructions/structure
○ Processus
● Fonction

Types de modèles inspirants
○ Animal
● Plante
● Champignon
○ Archea/Bacteria
○ Protozoaire
○ Chromista
● Écosystème

Type de connaissance utilisée
● Existantes pour le grand public
● Existantes pour le spécialiste
● Créées pendant la conception

Type d'apports en biologie
○ Historique/savoir du concepteur
○ Acquisition lors de la conception
● Biologistes intégrés au processus

RÉSULTATS & INNOVATION

Complexité de la construction
● Haute
○ Faible

Niveau d'innovation
○ Innovation de rupture
● Amélioration de l'existant

Revenir à la liste des projets

LEÇONS À RETENIR

Écotone est un des premiers projets immobiliers parisiens à initier une démarche de biomimétisme en 2017. Malgré le contexte sanitaire de 2020, il s'agit d'un projet dont les enjeux sont de conserver ces ambitions architecturales et environnementales. Le temps long de la conception permet de continuer de développer la démarche de biomimétisme. L'importance de l'intégration du biomimétisme dès la phase concours avec une acculturation des architectes et de la maîtrise d'ouvrage en amont du projet est un élément clé. La volonté de la maitrise d'ouvrage est une condition *sine qua non* pour la réalisation d'un projet biomimétique. La création d'un comité scientifique pour accompagner et challenger l'équipe projet dès la phase concours est une action peu commune dans un process de promotion immobilière. Cette intégration de scientifiques experts en biomimétisme et biodiversité dès la phase concours est également une des clés de réussite du projet.

Crédits des images :

[1] [2]

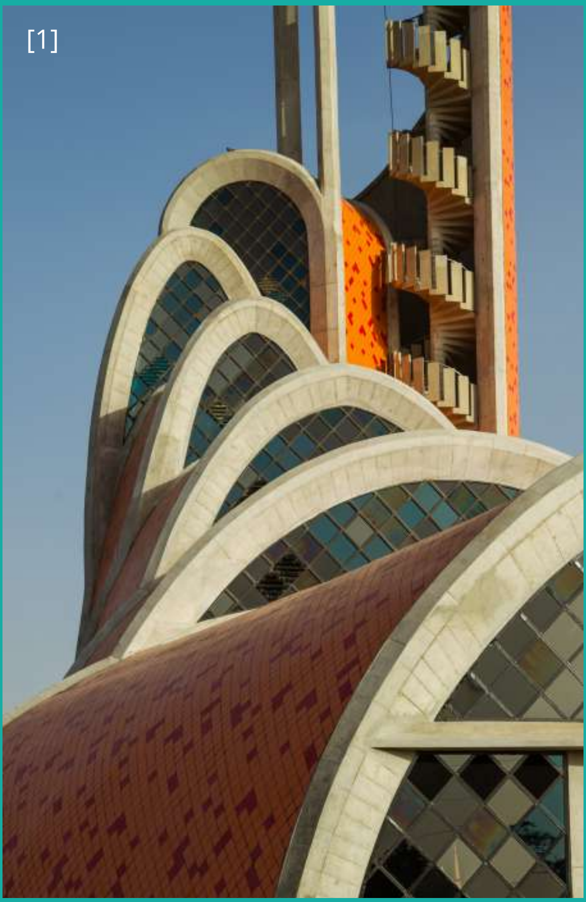

[1]

ÉGLISE DE NIANING

Adresse : Nianing, Sénégal
Statut du projet : construit
Date de conception : 2014
Type/usage : équipement
Rénovation : non
Climat : BWH - climat désertique chaud et sec
Surface du projet : 457 m² SDP
Coût de construction : 2 319 €/m²
Coût final du projet : 1 059 799 €

Membres du Biomim'City Lab engagés :

RÉSUMÉ

Ce projet se situe à Nianing à une centaine de kilomètres au sud de Dakar sur la « côte aux coquillages ». Afin de faire écho à cette particularité régionale, l'équipe en charge de la conception de cette église a pris comme point de départ la forme d'un cymbium.
Cette forme a été ensuite architecturée en fonction des contraintes constructives du programme et selon une orientation bioclimatique optimale.
Orienté vers l'est et élancé vers le ciel, l'édifice se referme au nord et au sud pour se protéger du soleil et du vent du désert de l'harmattan. L'édifice s'ouvre à l'ouest pour capter la fraîcheur des alizés par un jeu de volume décomposé en sept voûtes avec un clocher qui culmine à 45 mètres et qui joue le rôle à la fois de signal et de cheminée de ventilation.
Afin de mettre en place une ventilation passive naturelle, le projet s'inspire du fonctionnement de la termitière africaine qui est un modèle extrêmement efficace de régulation thermique. Le clocher est conçu comme une « cheminée de ventilation » qui assure un tirage thermique naturel et régulier tout au long de la journée et assure à l'édifice une fraîcheur constante.

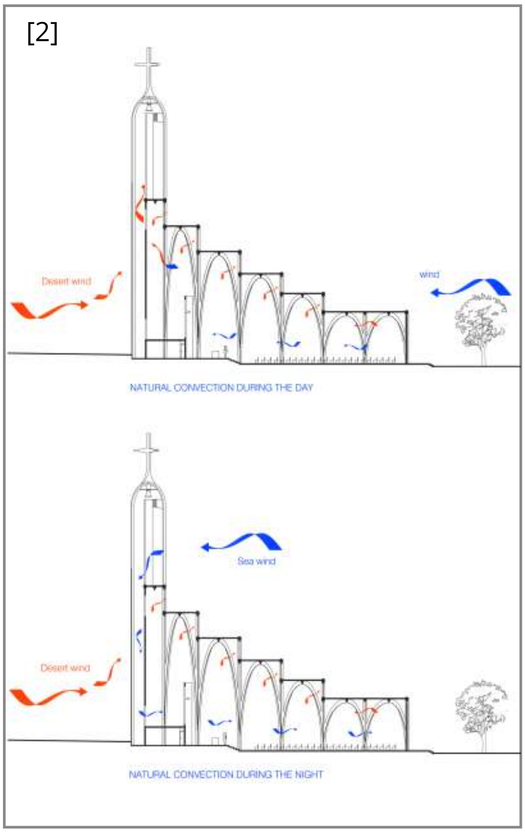

[2]

Rôle des principaux participants

Commanditaire : Archidiocèse de Dakar
Architectes : In Situ
BET structure : ETECS
BET acoustique : Impedance
Relai de maîtrise d'œuvre local : GA2D
Entreprise TCE : Eiffage

Coûts

Coût de construction : 2 319 €/m²
Coût final du projet : 1 059 799 €
Budget initial : 750 K € HT – aléa sol (présence d'argiles attapulites) qui a imposé la réalisation de micro-pieux à 18 mètres de profondeur.

Principaux prix

2020 • International Design Award, honorable mention
• Prix Afex Monde, nominé
2019 • Architecture Masterprize, winner
• Global Architecture and Design Award, winner
• Faith and Form, winner
• Green Solution Award, special mention

Certifications

Sans objet

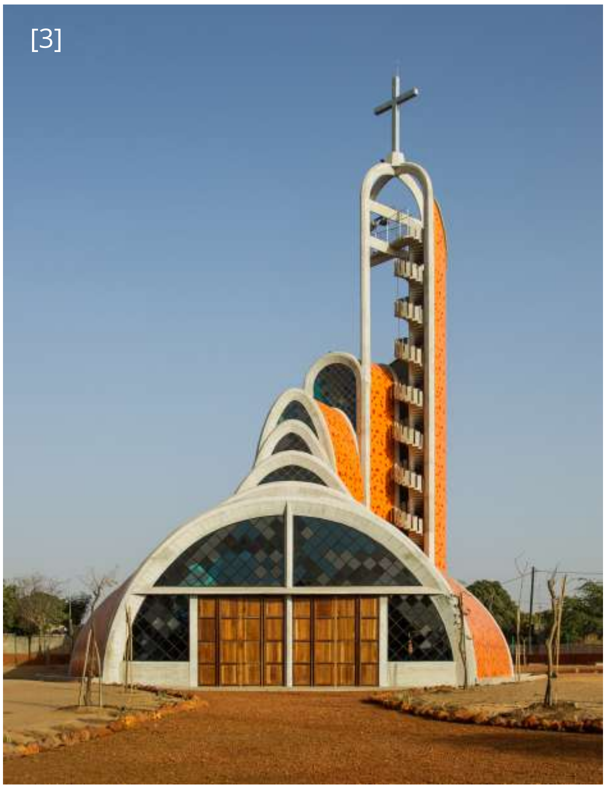

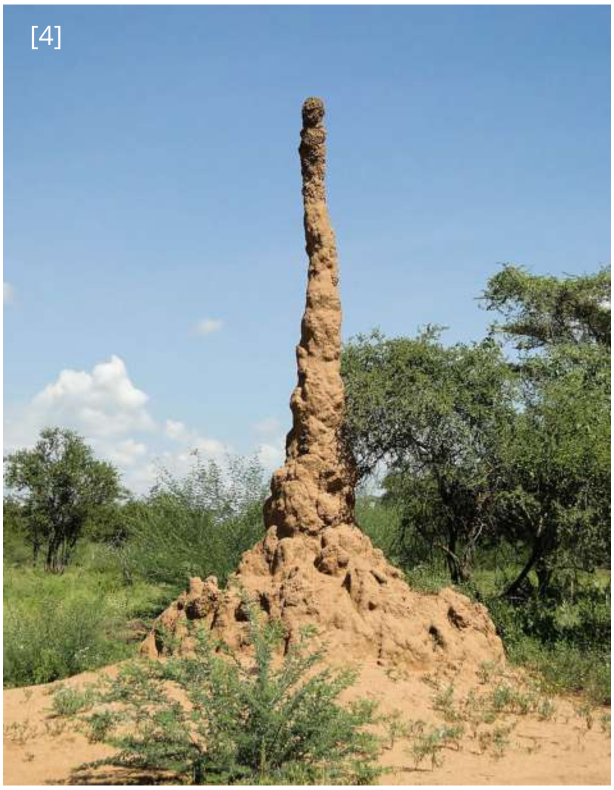

ANALYSE GLOBALE DU PROJET

Le cahier des charges défini par la maîtrise d'ouvrage dans le cadre de ce concours privé lancé en 2010 demandait à la maîtrise d'œuvre la réalisation d'une église d'environ 1 000 places sur la parcelle mitoyenne du presbytère de Nianing. Cela tient donc compte de la création d'une nef avec cœur, d'une sacristie, de toilettes accessibles et d'une esplanade extérieure permettant d'accueillir 500 personnes supplémentaires. C'est en 2014 que la première pierre de cette église a été posée pour une livraison en 2019. Dans le cadre de ces travaux le cahier des charges prévoyait que plus de 50 % de la main d'œuvre soit embauchée localement (région de Mbour). L'entreprise (Eiffage Sénégal) a embauché 32 personnes localement sur les 50 personnes qui travaillaient sur site.

Objectifs

L'édifice est fondé sur micropieux avec des longrines et une dalle portée. La superstructure est en béton armé hydrofugé. Le ciment est approvisionné localement par la cimenterie locale du Sahel de Kirené. Les arches sont coffrées par panneaux de contreplaqué de 2000 mm alors que les voûtes sont coffrées par voliges horizontales de 150 mm.
Les baies et menuiseries extérieures sont en acier finition peinture noire. Les carreaux de verres se déclinent selon trois couleurs : le bleu clair, le bleu foncé et l'incolore. Les carreaux qui recouvrent l'extérieur des voûtes sont émaillés et se déclinent selon deux couleurs : le orange et le rouge (les joints sont de couleur noir). Enfin les sols sont en ciment coquillé approvisionné localement sur la plage de Nianing.
À l'instar des costumes de cérémonie, l'édifice se drape à l'extérieur d'un « boubou » aux couleurs vives qui fait écho aux couleurs dominantes de la terre et du paysage : le rouge et l'orange.
À l'intérieur les couleurs s'effacent complètement pour favoriser le recueillement. Le béton brut de décoffrage blanc gris domine et les verrières introduisent une légère touche de couleur bleue qui évolue au fil de la journée. Le bleu correspond à un choix liturgique, c'est la couleur de la vierge.
La verticalité de l'édifice a été exploitée au maximum pour créer, le jour, une ventilation naturelle par « effet cheminée » et la nuit le système se retourne par convection naturelle.
Ce rafraîchissement par tirage thermique permet de conserver une température intérieure de 23 à 28 °C et permet d'économiser 112 % de la consommation énergétique du bâtiment. Le total des émissions de GES est de 0.3 $KgCO2/m^2/an$.
Au pied des voûtes ont été créées de larges cunettes qui sont reliées de façon gravitaire à des bassins de rétention paysagers aménagés en contrebas du site. Les bassins de rétention compensent l'imperméabilisation créée par l'édifice et permettent l'infiltration d'eau progressive dans les sols lors de la saison des pluies.

Revenir à la liste des projets

ANALYSE BIOMIMÉTIQUE

Conception biomimétique

Située dans une zone tropicale sèche l'église de Nianing a été conçue afin d'assurer le confort thermique du batiment à ses usagers. L'un des enjeux majeurs de ce projet était de trouver un moyen de rafraîchir en permanence l'édifice, de façon passive, sans recours à aucun système mécanique.

• **La termitière comme modèle :** La présence à proximité du site, de termitières de forme très verticale a interrogé les architectes qui ont alors essayé de comprendre le fonctionnement de ce modèle biologique, en particulier via l'analyse qu'en a faite l'architecte Mike Pearce. Alors que la température extérieure peut monter à plus de 40°C, la termitière arrive à conserver une température moyenne intérieure comprise entre 20 et 25 °C. Le modèle de fonctionnement est en fait assez complexe et mêle à la fois principe passif, par effet de cheminée, et principe actif, opéré par les termites elles-mêmes. Par ailleurs ces termitières, constituées de terre, jouent également un rôle important dans l'équation.
L'équipe en charge de la conception de l'église de Nianing s'est ainsi inspirée de ce fonctionnement biologique et de ses principes de ventilation passive. Le clocher de l'église s'inspire ainsi du système de cheminée de ces termitières. En effet, plus la différence d'altimétrie entre l'entrée d'air et la sortie d'air est importante et plus le flux d'air est rapide. Ce balayage de l'air intérieur créé un mouvement d'air sur notre peau qui donne une sensation de fraîcheur.

• Un autre **facteur de nature géo-climatique** a permis le fonctionnement efficace de l'effet cheminée, la proximité de la masse de l'océan et le principe de la convection thermique qui crée quotidiennement un flux d'air entre la mer et la terre. Au final c'est la combinaison entre le modèle de fonctionnement de la termitière et les caractéristiques géo-climatiques du site qui permet a création d'une surventilation par effet ascendant qui rafraîchit l'édifice.

Impacts documentés du projet

• **Fraîcheur et confort :** l' Église de Nianing est un lieu de rassemblement à vocation sociable et sa fraicheur en fait un lieu hospitalier propice au rassemblement en dehors des heures de messe.

• **Vers l'utilisation d'un béton moins énergivore :** sur le plan environnementale, l'impact écologique du béton est globalement négatif car très énergivore lors de sa phase de production. À l'origine du projet l'équipe de ce projet avait imaginé l'utilisation de briques geo-ciment (mélange terre ciment) mises en œuvre selon le principe de la voûte nubienne mais très vite la maîtrise d'œuvre s'est heurtée à une incompréhension culturelle locale (aussi bien de la part du maître d'ouvrage que la communauté religieuse de la paroisse).

• **Le clocher, joue le rôle d'un phare et de repaire, de nuit pour les pêcheurs :** une surprise qui n'avait pas été prise en compte dans la conception du projet. Au-delà de sa fonctionnalité, l'édifice apporte donc un service important à la communauté des pêcheurs , une des activités économiques principales de la région.

• **Le clocher de l'église de Nianing, un perchoir aux oiseaux :** le clocher de l'église constitue aussi un perchoir à oiseaux et ceci au grand dam de l'abbé qui fait nettoyer de façon hebdomadaire l'escalier qui mène à la cloche.

Impacts sur la recherche

Ce projet a été l'occasion pour In Situ de démarrer une démarche de recherche et développement que les architectes poursuivent en parallèle de leurs projets. Le cœur de leurs travaux de recherche et développement se concentre, plus particulièrement, sur la thématique de « la ventilation naturelle passive dans les bâtiments ».

DÉMARCHE BIOMIMÉTIQUE

Approche
● Problem driven
○ Solution based

Origine de la bio-inspiration
● Opportunité/hasard/intérêt personnel
● Collaborations interdisciplinaires
○ Appel à projet
○ Autre

Performances ciblées
● Confort thermique
○ Confort visuel/lumineux
○ Confort acoustique
● Qualité de l’air intérieur
○ Qualité de l’air extérieur
○ Résistance aux contraintes mécaniques
○ Gestion de l’eau intérieur
○ Gestion de l’eau extérieur
○ Accueil de la biodiversité
● Adaptation au climat
○ Allègement de la structure
○ Gestion des déchets
○ Autres

Échelle d'intégration
○ Matériaux
○ Technologie
○ Système façade/toit/sol
● Bâtiment
○ Îlot
○ Quartier

PROCESSUS DE CONCEPTION

Utilisation d'un cadre de conception
● Oui
○ Non

Contraintes majeures
○ Manque de fonds
● Utilisation d'outils biomimétiques
○ Règlementations
● Problèmes techniques
○ Autres

Complexité de la conception
○ Haute
● Faible

LIEN AVEC LA BIOLOGIE

Niveau d'inspiration
● Organisme
○ Comportement
○ Écosystème

Dimension de l'inspiration
● Forme
○ Matériaux
○ Constructions/structure
● Processus
● Fonction

Types de modèles inspirants
● Animal : termitière *Macrotermes michaelseni*
○ Plante
○ Champignon
○ Archea/Bacteria
○ Protozoaire
○ Chromista
○ Écosystème

Type de connaissance utilisée
○ Existantes pour le grand public
● Existantes pour le spécialiste
○ Créées pendant la conception

Type d'apports en biologie
○ Historique/savoir du concepteur
● Acquisition lors de la conception
○ Biologistes intégrés au processus

RÉSULTATS & INNOVATION

Complexité de la construction
○ Haute
● Faible

Niveau d'innovation
○ Innovation de rupture
● Amélioration de l'existant

Revenir à la liste des projets

LEÇONS À RETENIR

Élaborer un projet de ce type demande un certain niveau de maturation.
Il existe aujourd'hui une incompatibilité temporelle entre le temps de conception lors d'un concours et le temps de recherche et développement. Pour autant, la phase de recherche est nécessaire afin de construire des projets urbains biomimétiques.
Contrairement aux programmes de recherches et développement nécessitant un investissement de temps sur le long terme, la phase de conception lors des concours ne dure que quelques mois. Développer un programme de recherche nécessite ainsi la création de partenariat et une curiosité à toute épreuve.

Crédits des images :

[1] [2] [3] Régis L'Hostis/IN SITU Architecture

[4] NC

Revenir à la liste des projets

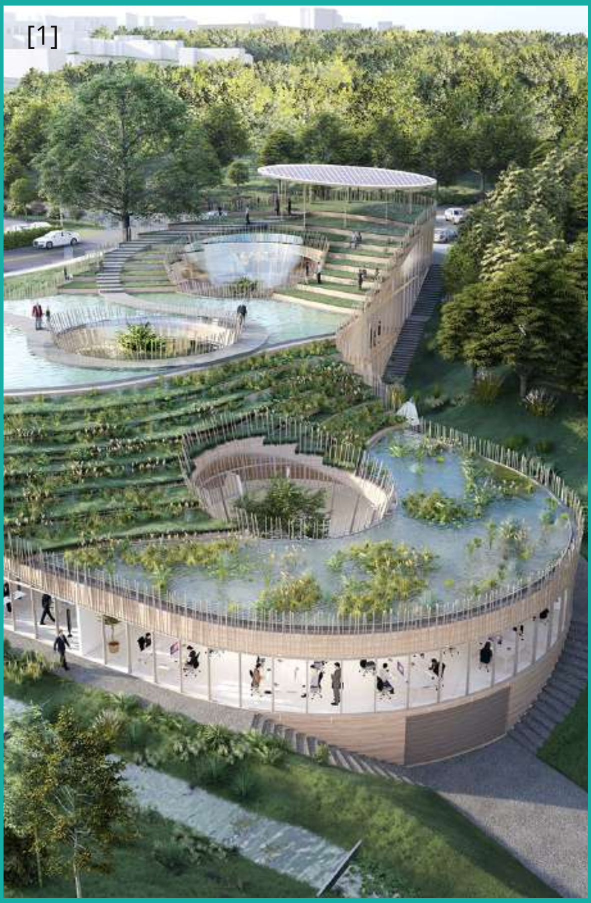
[1]

ESTRAN

Adresse : Biarritz, France
Statut du projet : non accepté
Date de conception : 2019
Type/usage : tertiaire
Rénovation : non
Climat : CFB - climat tempéré chaud, sans saison sèche et à été tempéré
Surface du projet : 2 900 m²
Coût de construction : 3 200 €/m²
Coût final du projet : 9 M€

Membres du Biomim'City Lab engagés :

bechu + associés

ELAN

RÉSUMÉ

L'ambition est de construire un « système vivant » et régénératif qui s'affirmera par une architecture à forte valeur symbolique. Celle d'un bâtiment nouvelle génération au rayonnement international, qui capitalisera sur la relation climat-océan pour faire naître un écosystème marin. Les 3 objectifs majeurs sont :

1. Positionner le Pays Basque comme référence sur le biomimétisme marin et contribuer au développement d'activités économiques innovantes et à forte valeur ajoutée pour le territoire avec l'accueil.

2. Créer un bâtiment à la fois vitrine mais aussi vecteur des activités scientifiques et économiques qu'il hébergera, au travers d'une approche intégrée de l'innovation, développant des technologies et usages au travers du prisme d'un biomimétisme marin.

3. Tendre vers un bâtiment régénératif à impacts positifs pour le territoire basé sur les services écosystèmiques et guidé en partie par la grille d'évaluation de la certification Living Building Challenge TM (LBC).

Rôle des principaux participants

Maîtrise d'ouvrage : Communauté d'Agglomération du Pays basque
Architectes : agence d'architecture Bechu & Associés.
BET ingénierie du bâtiment structures, fluides : DVVD
BET Economie de la Construction : Axio SAS
Conseil environnement : Elan

Coûts

Coût de construction : 3 200 €/m²
Coût final du projet : 9 274 000 € HT

Principaux prix

Sans objet

Certifications

• LBC 3.0 utilisé comme cadre lors de la conception
• HQE pour la qualité d'éclairement des espaces

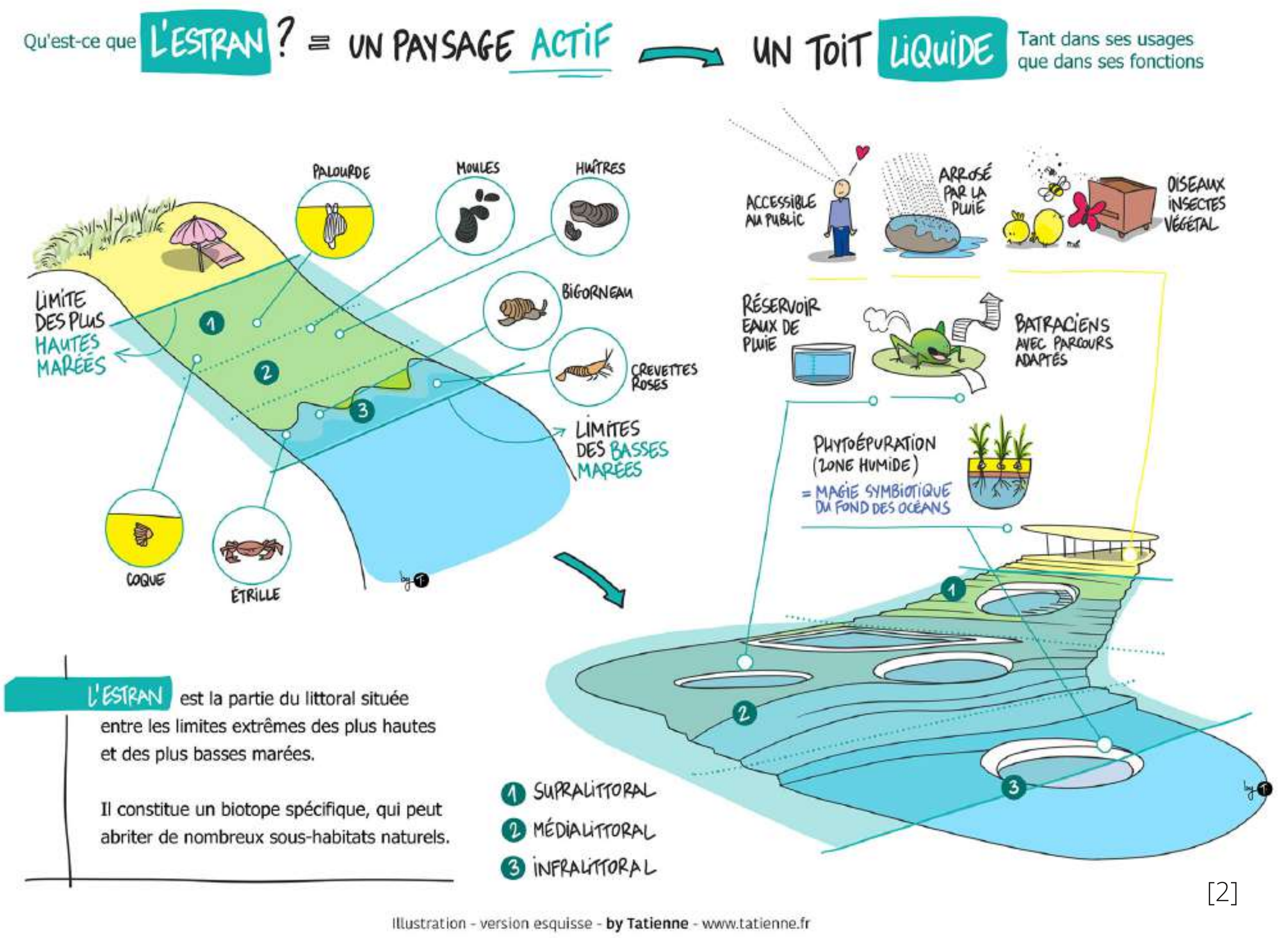

[2]

Illustration - version esquisse - by Tatienne - www.tatienne.fr

ANALYSE GLOBALE DU PROJET

La Communauté d'Agglomération du Pays basque a lancé en 2019 un dialogue compétitif pour l'attribution d'un marché de maîtrise d'œuvre pour le Pôle d'Excellence sur le Biomimétisme Marin à Biarritz selon un calendrier court et rythmé.
La ligne du projet architectural doit affirmer l'ambition portée par la Communauté d'Agglomération du Pays basque : concilier la dimension de l'impact de l'océan sur notre civilisation actuelle et sur son devenir, avec l'intelligence naturelle des êtres vivants qu'il abrite.
Le tout au bénéfice d'une nouvelle génération de bâtiments hybrides et régénératifs, pour que ce soit désormais la ville qui s'adapte aux grands cycles de la nature et non plus l'inverse.
Le projet proposé par l'agence Béchu et accompagné par le cabinet de conseil environnemental Elan trouve dans l'Estran un écosystème d'inspiration pour la conception de ce projet.

Objectifs

Les enjeux majeurs du projet :

• **Créer un écosystème** s'intégrant au sein d'un écosystème plus large en se servant de ses éléments, et interagissant avec lui à travers des services écosystémiques.

• Concevoir un lieu capable de **s'adapter au changement climatique**, pour ne pas en subir les effets (pluies, tempêtes, pollution...), en observant la relation climat/océan ainsi que les propriétés de résilience du monde des abysses.

• **« Ne pas déranger les voisins »** en composant un paysage habité qui offrira un nouveau lieu de promenade ouvert à tous et qui préservera la nature environnante. Un « paysage habité » dans lequel la nature pourra devenir « active » par les propriétés, notamment éducatives, qu'elle véhiculera.

• Créer une nouvelle **génération de bâtiment cadrée par le monde scientifique** — groupement representé par Françoise Gaill et Yves Tourre — et dont la démarche pourra même accompagner les actions mises en place par la plateforme internationale Climat/Océan coordonnée par Françoise Gaill.

Impacts positifs de l'Estran en termes de services écosystémiques

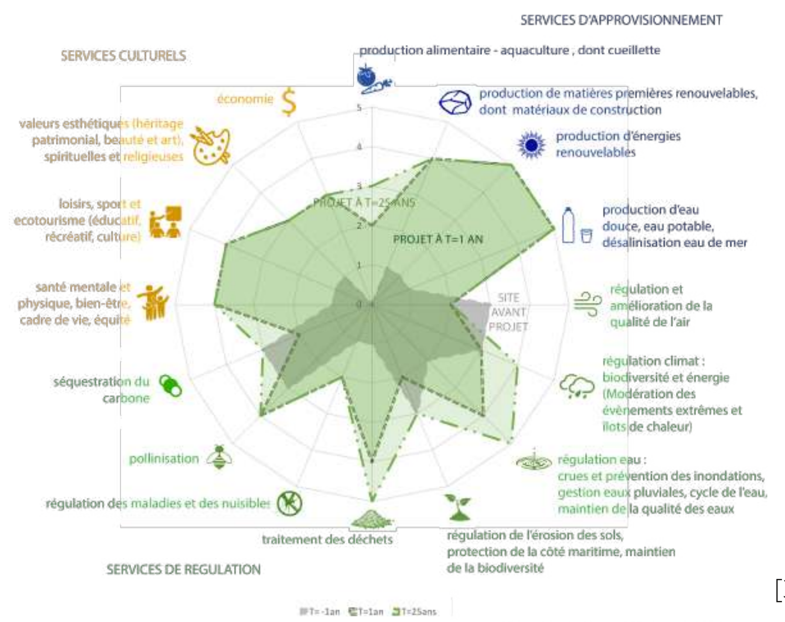

[3]

Conception biomimétique

L'approche biomimétique du projet a été portée par l'ensemble des acteurs à différentes échelles : une inspiration conceptuelle des mondes marins, une inspiration de la fonctionnalité de l'estran pour la toiture, un outil paramétrique pour optimiser la protection solaire de la façade et une vision écosystémique temporelle pour évaluer les impacts positifs du projet dans un écosystème.

• **Une toiture biomimétique active :** la toiture prévoit une zone humide qui s'inspire et reproduit le fonctionnement de l'estran. Cette zone humide servira de réservoir pour les eaux de pluie, de système de phytoépuration, d'habitat pour la biodiversité, comme d'un élément paysager accessible.

• **De la lumière naturelle :** la lumière plonge à travers de multiples puits lumineux pour éclairer les espaces et diminuer les consommations énergétiques.

• **Une façade bio-inspirée :** le confort thermique est assuré grâce à la conception paramétrique des façades inspirées, dont l'algorithme utilisé est lui-même inspiré par la nature, appelé Système Multi-Agents (SMA).

• **Une intégration avec la topographie :** le bâtiment utilise la contrainte de ce terrain en pente comme opportunité d'assise structurelle pour intégrer ce volume au territoire et permettre une quasi-disparition visuelle du projet sur le site.

• **La production des services écosystémiques :** le projet a estimé les services écosystèmiques du site avant le projet, après un et après 25 ans, afin de mesurer ses impacts positifs locaux.

• **Une stratégie sobre en matériaux :** composé de matériaux disponibles en Nouvelle Aquitaine, Estran profitera des savoir-faire locaux pour sa construction. La limitation de l'extraction de ressources minérales en préconisant un usage au strict minimum selon le principe de non-extraction et les matériaux biosourcés seront privilégiés.

• **Vers l'autosuffisance en eau :** un système de collecte des eaux pluviales, ainsi qu'un système de traitement des eaux noires et grises fonctionnant en boucle fermée, seront installés au sein de son site, limitant de ce fait ses rejets d'eau.

• **Consommations énergétiques minimisées et production d'énergie renouvelables :** Estran produit des ENR à hauteur de 121,3% des consommations totales du bâtiment. Les émissions de CO_2 supplémentaires à la normale sont évitées à travers une réflexion sur l'ensemble des choix des matériaux.

• **Estran participe à réguler les effets d'îlots de chaleur :** la végétalisation enrichie du site couplée à la présence d'étendues d'eau valorisent la régulation du phénomène d'îlot de chaleur urbain.

• **Création d'une structure en béton d'argile porteur créée grâce aux terres d'excavation :** le liant Materr'Up combiné aux déblais issus du chantier, permet la mise en œuvre d'un béton de site. C'est un béton autoplaçant, équivalent d'un béton de classe C25/30 formulé à partir d'une argile.

DÉMARCHE BIOMIMÉTIQUE

Approche
- ● Problem driven
- ○ Solution based

Origine de la bio-inspiration
- ○ Opportunité/hasard/intérêt personnel
- ○ Collaborations interdisciplinaires
- ● Appel à projet
- ○ Autre

Performances ciblées
- ● Confort thermique
- ● Confort visuel/lumineux
- ○ Confort acoustique
- ● Qualité de l’air intérieur
- ○ Qualité de l’air extérieur
- ● Résistance aux contraintes mécaniques
- ● Gestion de l’eau intérieur
- ● Gestion de l’eau extérieur
- ● Accueil de la biodiversité
- ● Adaptation au climat
- ○ Allègement de la structure
- ● Gestion des déchets
- ● Autres : performance énérgétique, s. culturels

Échelle d'intégration
- ○ Matériaux
- ● Technologie
- ● Système façade/toit/sol
- ● Bâtiment
- ○ Îlot
- ○ Quartier

PROCESSUS DE CONCEPTION

Utilisation d'un cadre de conception
- ● Oui
- ○ Non

Contraintes majeures
- ○ Manque de fonds
- ○ Utilisation d'outils biomimétiques
- ○ Règlementations
- ○ Problèmes techniques
- ● Autres

Complexité de la conception
- ● Haute
- ○ Faible

LIEN AVEC LA BIOLOGIE

Niveau d'inspiration
- ○ Organisme
- ○ Comportement
- ● Écosystème

Dimension de l'inspiration
- ○ Forme
- ● Matériaux
- ● Constructions/structure
- ● Processus
- ● Fonction

Types de modèles inspirants
- ○ Animal : Éponge de mer
- ○ Plante
- ○ Champignon
- ○ Archea/Bacteria
- ○ Protozoaire
- ○ Chromista
- ● Écosystème : marin, estran

Type de connaissance utilisée
- ○ Existantes pour le grand public
- ● Existantes pour le spécialiste
- ● Créées pendant la conception

Type d'apports en biologie
- ○ Historique/savoir du concepteur
- ○ Acquisition lors de la conception
- ● Biologistes intégrés au processus

RÉSULTATS & INNOVATION

Complexité de la construction
- ● Haute
- ○ Faible

Niveau d'innovation
- ○ Innovation de rupture
- ● Amélioration de l'existant

LEÇONS À RETENIR

Utiliser les ressources locales pour répondre aux besoins du projet (aux différents services écosystémiques) est un enjeu majeur. Parfois difficilement réalisable. La notion de coût de construction arrive généralement dans le processus de conception et oblige à réduire les ambitions.
Les propositions innovantes mais générant un changement économico-socio-culturel importants sont parfois un frein malgré une volonté initiale forte de bâtiment régénératif.
Dans le cadre d'un projet de construction d'un bâtiment régénératif, il semble important de pouvoir moduler la programmation de manière à ce que les usages puissent s'équilibrer les uns par rapport aux autres.

Crédits des images:

[1] [2] [3] © Agence d'Architecture A. Bechu & Associés

Revenir à la liste des projets

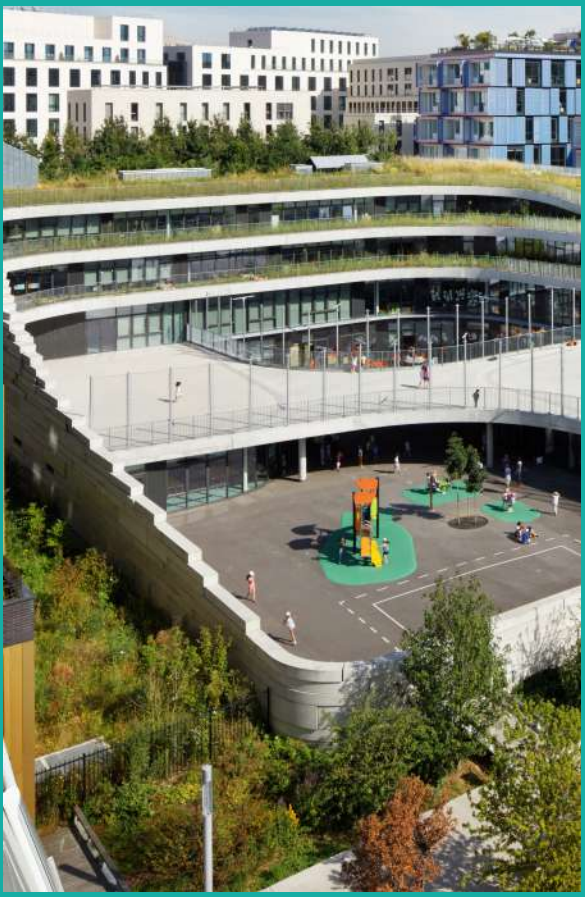

GROUPE SCOLAIRE DES SCIENCES & DE LA BIODIVERSITÉ

Adresse : 12-14 traverse Jules Guesde, 92100 Boulogne-Billancourt
Statut du projet : construit
Date de conception : 2010
Type/usage : équipement scolaire
Rénovation : non
Climat : CFB - climat tempéré chaud, sans saison sèche et à été tempéré
Surface du projet : 6 766 m^2
Coût de construction : -
Coût final du projet : 35 000 000 €

RÉSUMÉ

À la fin du 20e siècle Renault ferme les portes de ses usines de Boulogne-Billancourt léguant alors à la ville une immense friche industrielle constructible. En collaboration avec la Ville de Boulogne-Billancourt la SPL Val de Seine transforme alors cette friche industrielle en quartiers résidentiels culturels et économiques. Le Groupe Scolaire desSciences & de la Biodiversité se situe dans le quartier du Trapèze, l'un des premiers quartiers français à obtenir la certification écoquartier. Composé de 18 salles de classes et d'un gymnase dimensionné pour accueillir des compétitions régionales, cette école ouvre ses portes à la rentrée scolaire 2014 à 482 élevés, de la maternelle à l'élémentaire. Attenant aux salles de classes se trouve un gymnase dimensionné de manière à pouvoir y accueillir des compétitions régionales.
Perçu comme le poumon vert du quartier du Trapèze ce bâtiment participe à la restauration écologique de cette ancienne friche industrielle. Dotée d'un jardin sur son toit et d'une enveloppe dîte vivante cette école facilite l'accueil de la biodiversité en milieu urbain.
La conception offre aux utilisateurs du site et des bâtiments alentour l'illusion de se trouver non pas au cœur d'un environnement urbain dense, mais au sein d'un espace naturel.

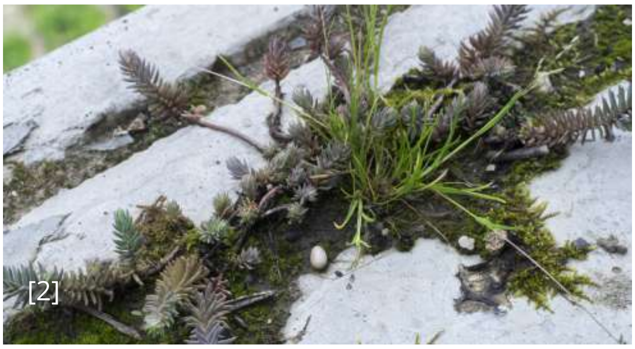
[2]

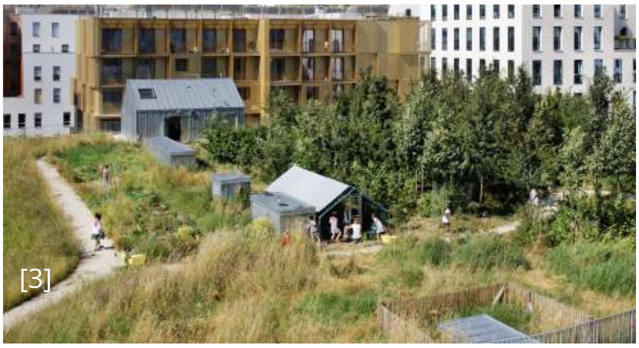
[3]

Rôle des principaux participants

Maîtrise d'ouvrage : SPL Val de Seine
Architectes coordinateurs : Loci Anima pour A4 est
Architecte : ChartierDalix
Maîtrise d'ouvrage : SPL Val de Seine
Paysagiste : Atelier d'Ecologie urbaine
Conseil en écologie Appliquée : Biodiversita, Atelier d'écologie urbaine, Hello Flore, Aurélien Huguet
Ouvrages publics : Bouygues
BET structure, fluide : EVP ingénierie, CFERM ingénierie
Recensement la biodiversité : ARB

Coûts

Coût final du projet : 35 000 000 €

Principaux prix obtenus

Sans objet

Certifications obtenues

• Certification HQE pour l'école
• Démarche HQE pour le gymnase

Inspiration et phase de test :

• Règlementation B&E+C pour inspiration
• Biodi(v)strict®

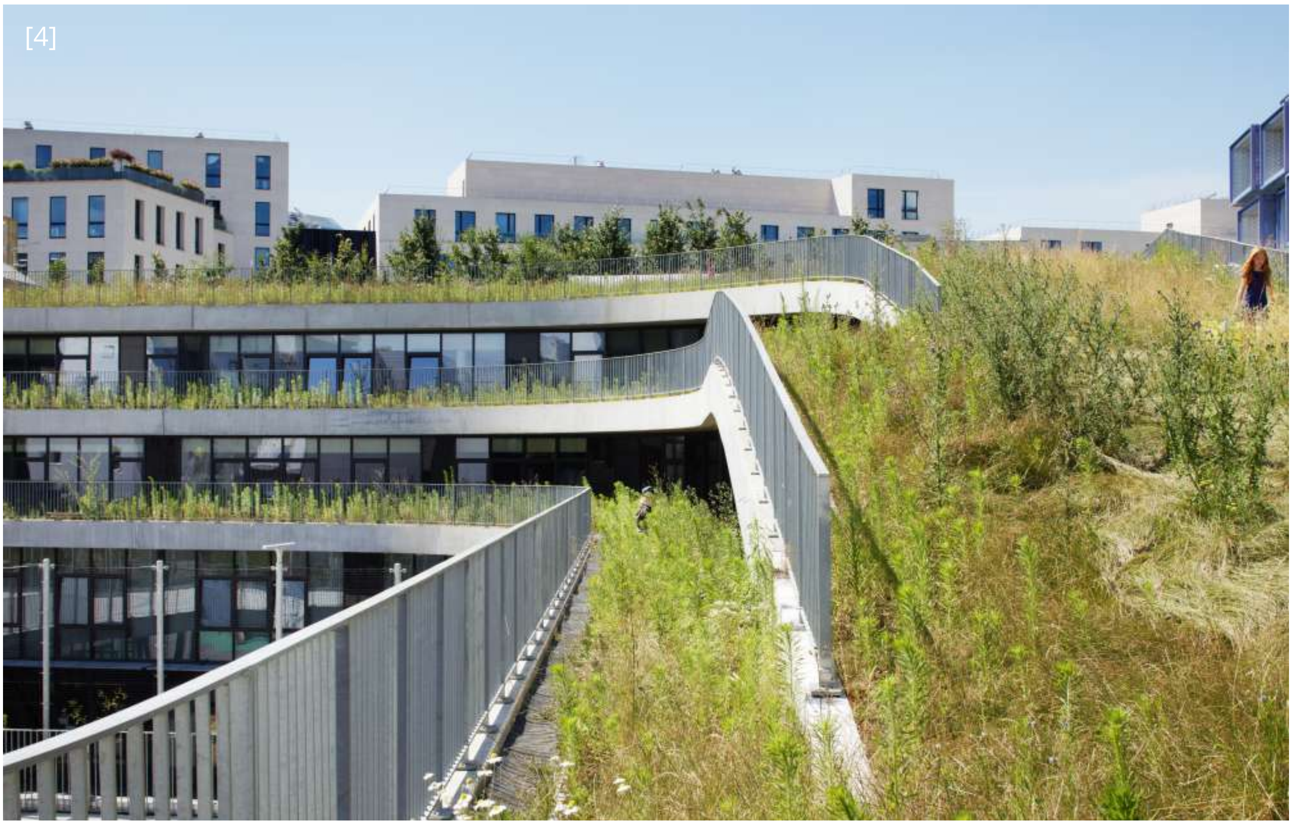

ANALYSE GLOBALE DU PROJET

Au début des années 2000 dans un contexte urbain dense SPL Val de Seine et la Maire de Boulogne-Billancourt avaient pour faire de l'ancienne friche industrielle des usines Renault un quartier à forte ambition écologique.
L'École des Sciences & de la Biodiversité se situe dans le quartier du Trapèze, l'un des premiers quartiers français à obtenir la certification écoquartier. Loci anima, l'agence d'architectes en charge de la conception du macro-lot A4 est, où ce situ le projet, souhaitait faire de cet îlot « un îlot urbain végétal colonisé par les Hommes, les enfants, les animaux et les végétaux ».

Dans le cadre du programme le maître d'ouvrage souhaitait que le bâtiment dispose d'un toit et d'un mur végétalisé. Les architectes de ChartierDalix sont allés plus loin que cela. L'ensemble du mur d'enceinte du bâtiment est habitable par la faune et flore locale. Le mur d'enceinte du bâtiment accueille donc un écosystème fonctionnel qui ne demande que très peu d'entretien.
Par son architecture audacieuse ce bâtiment assure la restauration de la biodiversité au sein d'un écosystème urbain dense, l'éducation des jeunes générations aux enjeux écologiques, la transmission d'un héritage durable générations futures.
À ce jour, le groupe scolaire accueille près de 500 élèves de la maternelle à la primaire et compte près de 20 classes. Dans l'enceinte du bâtiment se trouve aussi un gymnase, une cantine scolaire ainsi qu'un centre de loisir.

Avant d'aménager les nouveaux quartiers commerciaux et résidentiels de l'île Seguin et du Trapèze des diagnostics quant à la pollution des sols ont été menés. Ces études ont mis en évidence une forte concentration de polluants au sein de ces sols. Même si les terrains des anciennes usines Renault ont été décontaminés, une pollution résiduelle y subsiste (environ 0,2%). Il restait donc préférable de ne pas créer des jardins à même le sol. En aménageant un espace vert sur le toit, toute contamination de ce nouvel espace vert a été ainsi évitée. De plus la surface disponible au sol, dans le cadre de ce projet, n'était pas suffisante pour accueillir une telle superficie de biodiversité.
Par ailleurs il a aussi fallu réfléchir à la conception d'un mur de béton capable d'accueillir une biodiversité spontanée capable de survivre aux pressions environnementales.

Objectifs

• **Faire vivre en symbiose la faune, la flore et l'Humain :** face à l'urbanisation croissante de nos territoires l'Humain a ces dernières décennies dénoué tout lien avec la nature. Nos villes étant dépourvues de tout espace d'accueil de la biodiversité il devient néanmoins primordial d'y faire face en restaurant la biodiversité en ville. C'est d'ailleurs ce que tente de promouvoir les architectes ChartierDalix en imaginant le Groupe Scolaire des Sciences & de la Biodiversité. Pour cela ils profitent alors des espaces inutilisés pour en faire des espaces d'accueil de la biodiversité (toiture, murs, pied de murs).

• **Imaginer la ville de demain :** ce bâtiment tente de bousculer les normes architecturales urbaines actuelles afin d'imaginer les espaces urbains de demain, où y cohabiteraient l'Humain et la nature.

• **Un support pédagogique :** même si l'entièreté de la terrasse végétalisée n'est pas accessible aux enfants, un parcours et des espaces pédagogiques ont été aménagés dans le but de développer un programme éducatif relatif à l'écologie.

ANALYSE BIOMIMÉTIQUE

Conception biomimétique

• **Murs de vieux chemins et de citadelles :** ces murs, qui, au fil du temps, sont des support pour le développement de la biodiversité ont l'équipe en charge de la conception du mur d'enceinte englobant l'école, afin d'en faire une surface habitée. Les espèces végétales colonisant ce milieu sont, par ailleurs, aptes à résister aux variations thermiques et climatiques, et donc aptes à durer dans le temps.

• **Une falaise minérale :** afin de faire du mur d'enceinte un milieu extrudé, composé de failles, d'anfractuosités et de trous, l'équipe de conception du bâtiment s'est inspirée des parois des falaises pour fabriquer les 35 moules qui ont été utilisés pour façonnages des blocs de béton de ce mur.

• **1350 blocs de béton** constituent l'enveloppe, habitable, encerclant le bâtiment et une quinzaine de moules ont été utilisés pour façonner ces blocs. Disposés en quinconce ces derniers constituent, grâce à leurs cannelures, engelures, fissures, interstices, troues, un riche terrain de colonisation pour la végétation muricole, les oiseaux et les insectes.

• **Une centaine de nichoirs** : sont installés sur le mur, ils ont été dimensionnés pour accueillir les oiseaux présents *in situ*. Au sein de ce mur d'enceinte sont aussi disposés des hôtels à insectes.

• **Chemin de l'eau :** afin d'assurer un taux d'humidité suffisant sur le mur d'enceinte de l'école pour irriguer les espèces végétales le colonisant, un cheminement de l'eau a été élaboré au sein de l'enveloppe.

• **Le gymnase d'inspiration cavicole :** pensé comme une cave le gymnase tire ses lignes architecturales de ce milieu écologique.

• **Un toit végétalisé :** 1650 m^2 de jardin sur le toit, s'inspirant des forêts primitives franciliennes.

• **Un toit divisé en 3 strates :** une prairie de fauche, une strate de fruticée, et une strate de chênes et charmes, reconstituant ainsi le biotope du sous-bois et de la prairie. Ces trois strates sont interconnectées afin de permettre aux espèces de circuler d'un milieu à un autre. Un potager ainsi qu'un poulailler ont par la suite été installés sur le toit.

• **Un écosystème interconnecté :** le bâtiment est un écosystème interconnecté dont le mur d'enceinte, habité, fait la jonction entre le sol et la toiture végétalisée.

• **Une conception paysagère :** jouant sur des courbes douces et fluides le bâtiment s'inspirent des lignes des paysages naturels, assurant alors un confort visuel et évitant tout phénomène de ruptures. Cette école donne ainsi l'impression de se retrouver à la campagne au sein d'un paysage extrudé.

• **4 différents milieux d'accueil de la flore :** les pieds de murs, les murs, la forêt et les prairies assurent l'accueil de la flore locale.

• **Espèces locales :** en amont des travaux de construction du bâtiment, une étude a été menée, sur trois ans, afin de recenser les espèces d'oiseaux présentes *in situ*, menant ainsi à l'intégration au sein du mur d'enceinte de nichoirs adaptés. Il en est de même pour les espèces floristiques présentes sur la terrasse végétale de l'école.

• **Éclairage naturel des salles de classes :** les fenêtres des salles de classes sont orientées du côté cours offrant ainsi un puit de lumière naturelle à ses utilisateurs.

Impacts documentés du projet

L'étude faunistique et floristique menée en 2016 a révélé que sur les 114 espèces observées dans l'enceinte du bâtiment 70 d'entre elles étaient des espèces spontanées.

Le suivi écologique mené pendant 3 ans après la livraison par l'Agence Régionale de la Biodiversité (ARB) a recensé :

• 15 espèces, dont 10 spontanées, sur le mur d'enceinte de l'école ;

• 28 espèces, dont 13 spontanées, au pied du mur d'enceinte ;

• 52 espèces, dont 44 spontanées, au sein de la prairie

• 35 espèces, dont 19 spontanées, au sein de la forêt.

DÉMARCHE BIOMIMÉTIQUE

Approche
- ● Problem driven
- ○ Solution based

Origine de la bio-inspiration
- ● Opportunité/hasard/intérêt personnel
- ○ Collaborations interdisciplinaires
- ○ Appel à projet
- ○ Autre

Performances ciblées
- ○ Confort thermique
- ● Confort visuel/lumineux
- ○ Confort acoustique
- ○ Qualité de l'air intérieur
- ○ Qualité de l'air extérieur
- ○ Résistance aux contraintes mécaniques
- ○ Gestion de l'eau intérieur
- ● Gestion de l'eau extérieur
- ● Accueil de la biodiversité
- ○ Adaptation au climat
- ○ Allègement de la structure
- ○ Gestion des déchets
- ○ Autres

Échelle d'intégration
- ○ Matériaux
- ○ Technologie
- ● Système façade/toit/sol
- ● Bâtiment
- ○ Îlot
- ○ Quartier

PROCESSUS DE CONCEPTION

Utilisation d'un cadre de conception
- ● Oui
- ○ Non

Contraintes majeures
- ○ Manque de fonds
- ○ Utilisation d'outils biomimétiques
- ○ Règlementations
- ● Problèmes techniques
- ○ Autres

Complexité de la conception
- ● Haute
- ○ Faible

LIEN AVEC LA BIOLOGIE

Niveau d'inspiration
- ○ Organisme
- ○ Comportement
- ● Écosystème

Dimension de l'inspiration
- ○ Forme
- ● Matériaux
- ● Constructions/structure
- ○ Processus
- ● Fonction

Types de modèles inspirants
- ○ Animal
- ○ Plante
- ○ Champignon
- ○ Archea/Bacteria
- ○ Protozoaire
- ○ Chromista
- ● Écosystème : prairie, forêt, milieu cavicole, murs citadelles

Type de connaissance utilisée
- ● Existantes pour le grand public
- ● Existantes pour le spécialiste
- ○ Créées pendant la conception

Type d'apports en biologie
- ● Historique/savoir du concepteur
- ● Acquisition lors de la conception
- ● Biologistes intégrés au processus

RÉSULTATS & INNOVATION

Complexité de la construction
- ○ Haute
- ● Faible

Niveau d'innovation
- ● Innovation de rupture
- ○ Amélioration de l'existant

Revenir à la liste des projets

LEÇONS À RETENIR

• **Construire non pas une structure végétalisée mais vivante :** il n'est pas rare que les façades et toits végétalisés aménagés sur nos bâtiments urbains soient de grands consommateurs d'eau et d'énergie. Le Groupe Scolaire des Sciences & de la Biodiversité prouve qu'il est possible d'ériger un mur habité et une toiture végétalisée ne demandent que très peu d'entretien.

• **Faire du bâtiment un outil pédagogique :** le toit végétalisé du bâtiment un riche support pédologique pour les enseignants de l'établissement.

• **Constituer une équipe de conception interdisciplinaire :** architectes, écologues, paysagistes, etc. ont apporté leurs connaissances et compétences afin de bâtir une école accueillant une biodiversité endémique et s'intégrant dans son écosystème urbain.

• **Imaginer un bâtiment inachevé :** inachevé le mur d'enceinte est amené à évoluer dans le temps, favorisant ainsi l'accueil d'une nouvelle faune et flore spontanées.

• **Conception des blocs de béton :** Afin de faire du mur un espace d'accueil de la biodiversité il a fallu réfléchir aux différents types de moulage des blocs de béton, ainsi que leur positionnement au sein de la structure afin que les failles, trous et cavités soient adaptées à la faune et flore locales, capables de résister aux pressions environnementales.

Crédits des images:

[1] [2] [3] [4] ChartierDalix - Takuji Shimmura

Revenir à la liste des projets

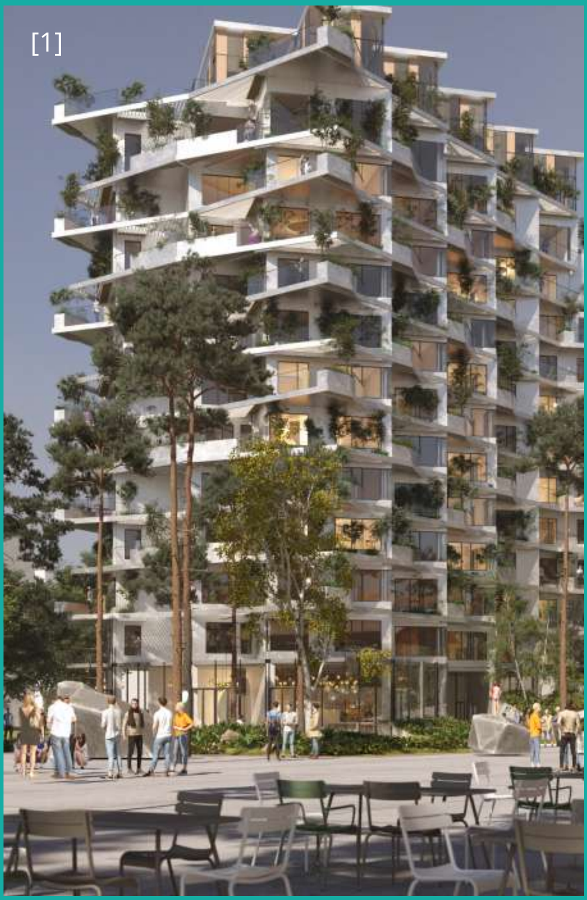
[1]

OSEZ JOSÉPHINE

Adresse : Rueil Malmaison, 92 500 - Zac de l'Arsenal
Statut du projet : IMGP2 – projet finaliste, non retenu
Date de conception : 2019
Type/usage : écoquartier usage mixte
Rénovation : partielle
Climat : CFB - climat tempéré chaud, sans saison sèche et à été tempéré
Surface du projet : 20 105 m²
Habitations : 16 375 m²
Commerces : 3 730 m²
Coût de construction : -
Coût final du projet : -

Membres du Biomim'City Lab engagés :

RÉSUMÉ

Situé au cœur du nouveau village de Rueil que représente la Zac de l'Arsenal, ce projet suit avant tout une démarche d'urbanisme régénérateur fondée sur un travail paysagé, biomimétique et bioclimatique. L'enjeu étant de définir les repères fondamentaux de l'écoquartier en train de naître, pour proposer aux habitants des signes d'identification facilement appropriables, à leur échelle, facilitant la présence de la nature et le bien-être de tous. Ces signes, ont été d'abord cherchés dans le grand paysage de la Métropole. Une échelle à laquelle on distingue les coteaux de la Seine qui grimpent et culminent au Mont Valérien, et marquée par les silhouettes des tours de la Défense qui émergent au Nord. Des images fortes déjà très présentes et signifiantes dans l'esprit de la majeure partie des habitants du bassin et au-delà, que le projet réinterprète en naissant de ce paysage très marquant, et qui s'organise autour de la place principale.

C'est ainsi qu'à l'image de la future gare du Grand Paris Express (GPE), des « collines habitées » longent la place, puis s'inclinent vers le parc traversant où elles rejoignent le sol. Des terrasses végétalisées accessibles à tous y ondulent et se plissent au-dessus du rez-de-chaussée. Sur ces « collines » émergent des habitations elles-mêmes fortement végétalisées, qui donnent accès au grand paysage et à ses éléments iconiques sus nommés. Le tout, à l'image de la nature observée, suit une démarche résolument biomimétique pour permettre à la ville de Rueil d'affirmer un positionnement d'avant-garde : aller plus loin que l'intégration de la nature à la ville en intégrant la ville elle-même aux grands cycles de la nature.

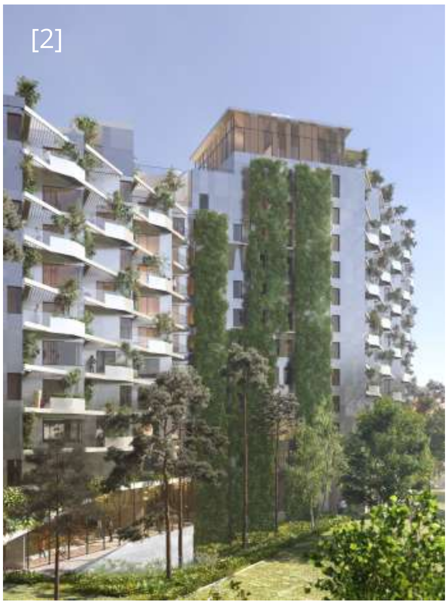
[2]

Rôle des principaux participants

Maîtrise d'ouvrage : Linkcity et Care Promotion
Architectes : Agence d'Architecture A. Bechu & Associés
Programmation & conception : Architecte associé Dream, Agence Babylone, Bouygues IDF, CDC Habitat
Partenaires Agora alimentation durable : Alliance slow food, Fondation Goodplanet, l'Atelier des Chefs
Partenaires innovations sociales : Hey Co, Have a good day, La Poste, Le cercle
Partenaires innovations écologiques : Ceebios, Le cercle, Reverdir, Decotropy, Moulinot, Incet, On, Embix, Elan, Inddigo

Coûts

Sans objet

Principaux prix

Sans objet

Certifications

• Visées : Well Community Pilot, Well Residential Pilot et BiodiverCity

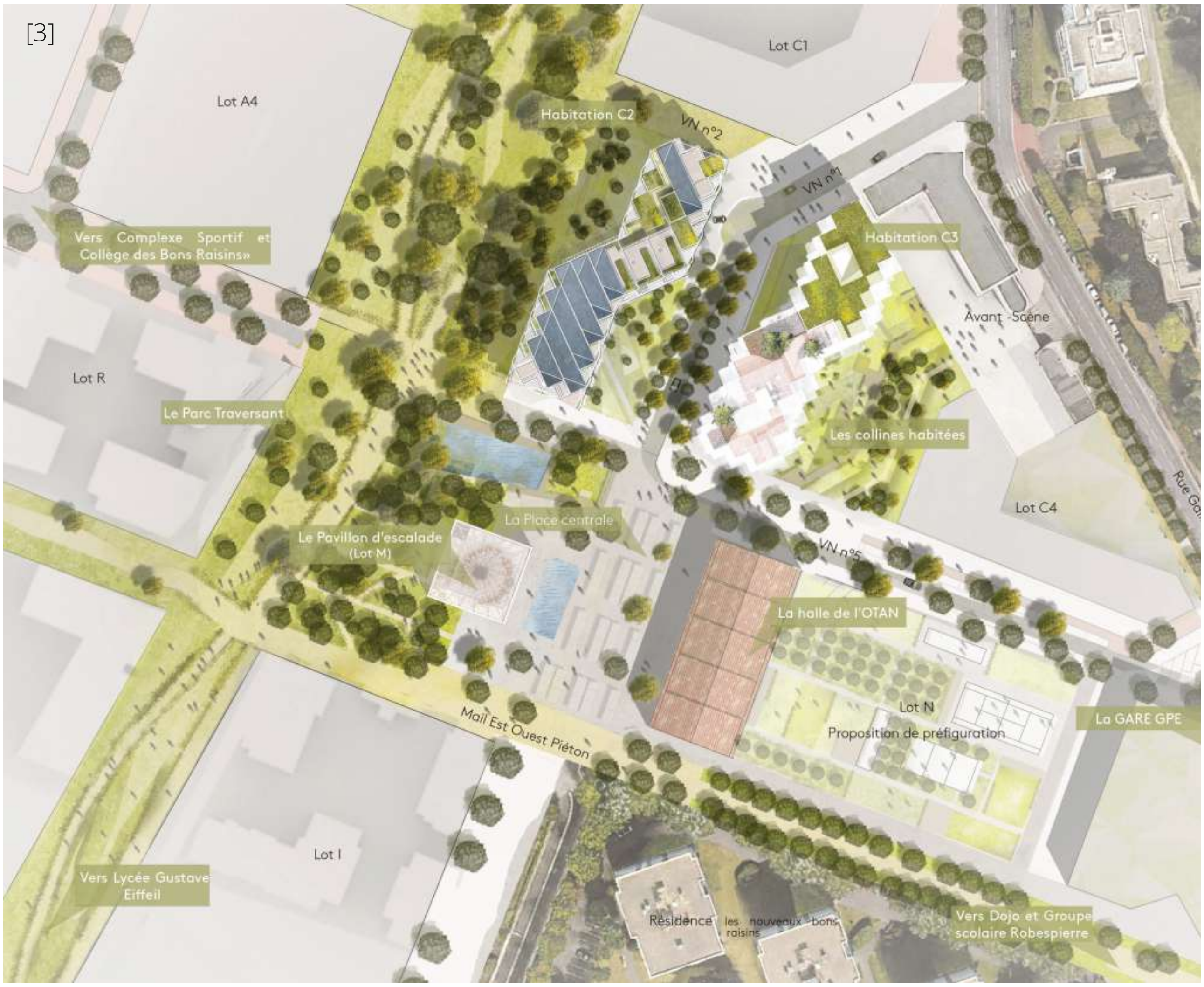

Plan masse du projet Osez Joséphine : Intégrer la nature à la ville pour intégrer la ville dans le grand cycle de la nature

ANALYSE GLOBALE DU PROJET

Dans le cadre d’un Appel à projets urbains innovants devant incarner la vision et l’identité partagée de la Métropole du Grand Paris, l’agence d’architecture A. Bechu & Associés a conçu le projet d’écoquartier Osez Joséphine à Rueil-Malmaison.

Située à environ 8 km à l’Ouest de Paris, la ville de Rueil-Malmaison compte près de 80 000 habitants. Commune la plus étendue des Hauts-de-Seine (environ 1 474 hectares), Rueil-Malmaison se caractérise également par une forte présence de biodiversité sur son territoire puisqu’elle compte 520 hectares d'espaces verts, soit plus d'un tiers de sa superficie totale.

Le départ annoncé des bâtiments de bureaux du groupe Renault et de l’OTAN, a conduit la Ville à envisager concrètement la recomposition et le réaménagement de ce site d’activités. En effet, cette zone d’environ 26 hectares composée d’anciennes friches industrielles, représentait une fracture urbaine importante. La volonté municipale est de réaliser un projet d’aménagement d’envergure, ayant pour finalité la constitution d’une nouvelle centralité rueilloise.

L’objectif principal du projet est de reconquérir cette fraction du territoire communal afin d’y créer une nouvelle centralité, comprenant une pluralité d’activités et d’équipements. L'écoquartier proposera également une offre de logements, d'emplois et de services diversifiés avec des constructions qualitatives et vertueuses sur le plan environnemental.

En ambitionnant de créer un quartier pour ses usagers, l’équipe de la conception suit les objectifs de la certification WELL, une démarche holistique dont l'objectif est d’améliorer le confort, la santé et le bien-être des individus, tant à l’intérieur de l’environnement bâti qu’au travers de la qualité des espaces publics.

Grâce à son travail mené à la fois sur l’écosystème forestier et le projet paysager, la maîtrise d’œuvre ambitionne d’obtenir la labellisation BiodiverCity, par la prise en compte des enjeux de biodiversité, dès la conception du projet.

Objectifs

Le paysage du nouveau quartier de l’Arsenal est composé de coteaux qui culminent au Mont Valérien. La présence de biodiversité dans l’écosystème environnant est forte et offre un « vivier vert » sur lequel greffer le futur quartier. Ainsi, l’écosystème caractéristique de la pinède présent en région parisienne (Forêt de Fontainebleau…), est le principal habitat naturel que l’équipe du projet a choisi de mettre en avant, et à partir duquel ils ont développé la démarche biomimétique. En effet, la durabilité d’un projet résidant aussi dans le respect du *genus loci*, ils ont souhaité que ce nouveau quartier fasse le lien avec l’histoire, en faisant écho au cèdre de Marengo (arbre de la famille des pins), « personnage » bicentenaire emblème du Parc de Rueil Malmaison offert en son temps par Joséphine de Beauharnais à son mari Napoléon, suite à la victoire de la bataille de Marengo.

C’est donc à partir de l’écosystème de la forêt, et de la stratégie phyllotaxique propre aux aiguilles des pins, qu'un travail biomimétique à été mené, à toutes les échelles et composantes du projet. Pour l’équipe en charge de la conception de cet écoquartier, le biomimétisme a été un véritable outil mis au service du projet urbain et architectural, tant dans l’organisation des programmes que dans la projection des usages.

ANALYSE BIOMIMÉTIQUE

Conception biomimétique

Dans le cadre de ce projet la maîtrise d'oeuvre a cherché à créer un cœur de quartier qui soit un écosystème entier à l'instar de ceux que la nature crée. Osez Joséphine invente ainsi les conditions d'un écosystème pérenne en s'inspirant de celui des forêts franciliennes et notamment celle de Fontainebleau. Les principales solutions sont :

• **La phyllotaxie pour l'organisation des bâtiments :** pour les bâtiments de logements la capacité des plantes à orienter leurs feuilles vers la lumière, a été utilisée. Cela a permis à chaque unité d'habitation de suivre un plan d'étage « phyllotaxique » de sorte à capter le maximum de lumière. L'objectif de cela est d'offrir plus de lumière l'hiver et une protection aux pics de chaleur en été dans les heures les plus chaudes grâce à des systèmes passifs de protection solaire. Les immeubles d'habitations sont composés d'une juxtaposition d'appartements indépendants. Chacun d'entre eux n'occupe qu'une petite partie du volume du bâtiment et ne dispose que d'une portion de ses façades.

• **Un projet évolutif :** l'immeuble peut ensuite, comme une branche, se développer dans la direction la plus favorable par prolifération, en supportant des feuilles qui se tournent systématiquement vers la lumière. Cette démarche biomimétique permet d'apporter un bioclimat positif à l'échelle de chaque appartement afin d'éviter toute ombre perpétuelle sur leurs façades ou dans leur environnement.

• **Une stratégie de végétalisation à plusieurs niveaux :** aux pieds des bâtiments des terrasses sont rendues à la végétation et ouvertes à la biodiversité. La nature monte littéralement à l'assaut des terrasses et façades des bâtiments pour rejoindre progressivement les toitures qui sont quant à elles dotées de serres partagées pour les habitants.

• **Un cycle de l'eau optimisé :** l'intégration de rétenteurs biosourcés Reverdir® et intégration de photobioréacteurs pour le traitement des eaux grises FREEWATERBOX®.

• **Valorisation des déchets :** une ressourcerie, un service de compostage et un objectif « tout compostable » à travers l'Agora de l'alimentation.

• **Récupération et production d'énergie :** une centrale photovoltaïque en toiture de la halle de l'OTAN, récupération de l'énergie via des serres des pompes à chaleur air-eau, récuperation de chaleur des eaux grises.

Impacts documentés du projet

La place centrale de l'Arsenal devient le lieu de rencontres et d'échanges précieux que le nouveau village attend. Le projet présente une stratégie vaste pour une réintégration de la ville au fonctionnement des écosystèmes naturels. Plus de 30 pins et cèdres se dressent dans le parc, sur la place, les trottoirs et tous les espaces libres.
La halle de l'Otan évoque le passé du site. Ses nouvelles façades en terre banchée et sa toiture en bardeaux de bois proposent des pistes vertueuses et durables de développement.
En face, dans la pinède, à l'orée du parc, se dresse une structure arborescente en bois qui fait renaître « le Cèdre ». Elle est enveloppée de façades transparentes et offre à des grimpeurs des parcours originaux dans un cadre naturel exceptionnel, elle présente aux passants les plaisirs d'un sport original et propose un exemple d'activités adaptées à la vie trépidante de la Métropole.

DÉMARCHE BIOMIMÉTIQUE

Approche
- [x] Problem driven
- [x] Solution based

Origine de la bio-inspiration
- [ ] Opportunité/hasard/intérêt personnel
- [x] Collaborations interdisciplinaires
- [x] Appel à projet
- [ ] Autre

Performances ciblées
- [x] Confort thermique
- [x] Confort visuel/lumineux
- [ ] Confort acoustique
- [x] Qualité de l'air intérieur
- [x] Qualité de l'air extérieur
- [x] Résistance aux contraintes mécaniques
- [x] Gestion de l'eau intérieur
- [x] Gestion de l'eau extérieur
- [x] Accueil de la biodiversité
- [x] Adaptation au climat
- [x] Allègement de la structure
- [x] Gestion des déchets
- [x] Autres

Échelle d'intégration
- [x] Matériaux
- [x] Technologie
- [x] Système façade/toit/sol
- [x] Bâtiment
- [ ] Îlot
- [ ] Quartier

PROCESSUS DE CONCEPTION

Utilisation d'un cadre de conception
- [x] Oui
- [ ] Non

Contraintes majeures
- [ ] Manque de fonds
- [x] Utilisation d'outils biomimétiques
- [ ] Règlementations
- [ ] Problèmes techniques
- [ ] Autres

Complexité de la conception
- [x] Haute
- [ ] Faible

LIEN AVEC LA BIOLOGIE

Niveau d'inspiration
- [x] Organisme
- [ ] Comportement
- [x] Écosystème

Dimension de l'inspiration
- [x] Forme
- [ ] Matériaux
- [x] Constructions/structure
- [ ] Processus
- [ ] Fonction

Types de modèles inspirants
- [ ] Animal
- [x] Plante
- [ ] Champignon
- [ ] Archea/Bacteria
- [ ] Protozoaire
- [ ] Chromista
- [x] Écosystème

Type de connaissance utilisée
- [ ] Existantes pour le grand public
- [x] Existantes pour le spécialiste
- [x] Créées pendant la conception

Type d'apports en biologie
- [ ] Historique/savoir du concepteur
- [x] Acquisition lors de la conception
- [x] Biologistes intégrés au processus

RÉSULTATS & INNOVATION

Complexité de la construction
- [ ] Haute
- [x] Faible

Niveau d'innovation
- [ ] Innovation de rupture
- [x] Amélioration de l'existant

LEÇONS À RETENIR

Ce projet a été l'occasion d'une démarche pédagogique d'explications de l'approche biomimétique et de son potentiel auprès de la maitrise d'ouvrage.
Celle-ci en a compris les opportunités réelles que l'outil biomimétique confère en matière d'innovation *low tech*, tout en constatant par ailleurs quelques blocages inhérents à l'ensemble de la chaîne de la construction pas ou peu éduquée au sujet. Les études menées en équipe avec la direction technique ont rapidement permis de convaincre de la capacité du projet à répondre aussi à un équilibre financier global.
Ce projet appuie la raison d'être de la démarche et de l'action du Biomim'City Lab, nécessaire à la mise en place d'une méthode éducative et intelligible par l'ensemble des acteurs de la « fabrique des territoires ».
N'ayant pas été lauréat de l'appel à projets, ce sujet précis ne verra pas le jour. Il a cependant donné naissance à une nouvelle famille de bâtiments de logements, qui en cette période de révolution écologique peuvent être conçus autrement, malgré les règlementations très cadrées concernant ce secteur.
L'ensemble des travaux de recherche menés par l'agence Bechu & Associés sur la stratégie phyllotaxique des plantes a permis d'implémenter leur outil de design paramétrique, et de faire naître ainsi un algorithme qui transpose l'approche architecturale bioclimatique à l'échelle de l'appartement, pour générer un confort d'été et un confort d'hiver sans apport de technologie complémentaire.

Crédits des images :

[1] [2] [3] © Agence d'Architecture A. Bechu & Associés

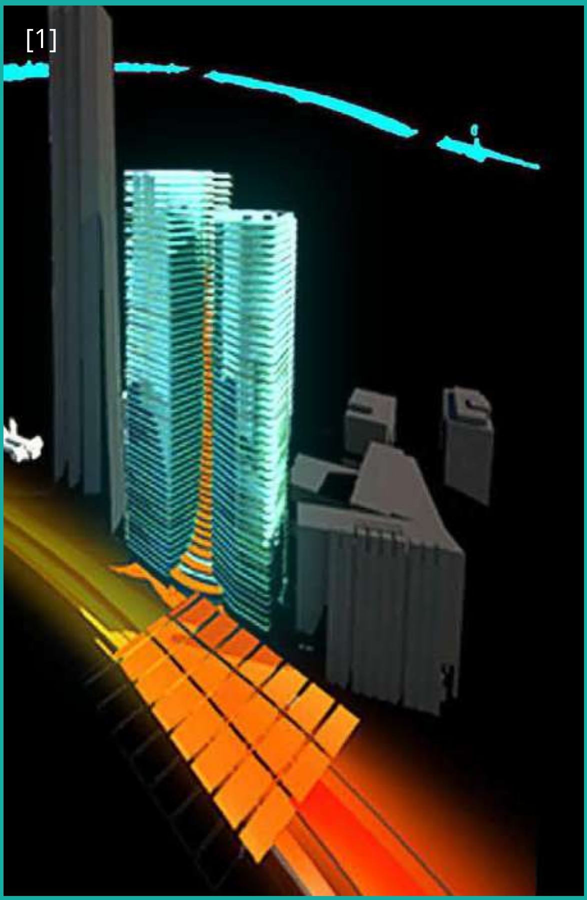

[1]

PARRAMATA

Tours de commerce bioluminescentes

Adresse : Mini Clones Pty Ltd, 89 Darcy St, Parramatta NSW 2150, Nouvelle-Galles du Sud, Australie
Statut du projet : projet non accepté
Date de conception : 2013
Type/usage : commerces
Rénovation : non
Climat : CFA - climat tempéré chaud sans saison sèche et à été chaud
Surface du projet : 120 000 m² SU
Coût de construction : 2 500 €/m^2
Coût final du projet : -

Membres du Biomim'City Lab engagés :

TANGRAM ARCHITECTES

RÉSUMÉ

La ville de Parramatta, située à 25 km de Sydney, ambitionne de concurrencer sa grande sœur. La ville mise donc sur un développement par l'innovation pour devenir un cœur battant dont le pouls résonnerait jusqu'à Sydney et l'océan. Au-delà des liaisons routières et ferrées, un lien naturel existe entre les deux cités : la rivière Parramatta, aux méandres intestinaux que les anguilles descendent et remontent inlassablement depuis toujours. Cette dimension historique et symbolique est puissante car elle relie également l'histoire des hommes, celle des aborigènes qui peuplent les lieux depuis 40 000 ans.
Ainsi, le projet de recherche s'appuie sur la connaissance des anguilles tachetées si caractéristiques de ce territoire et propose 2 tours de commerces totalisant 120 000 m², en connexion avec un pôle d'échange reliant directement Sydney et d'autres villes secondaires alentour. De cette façon, l'histoire des hommes, dans leur rapport à la nature, sert de ferment pour construire leur futur. Relier histoire et innovation.

Revenir à la liste des projets

Rôle des principaux participants

Commanditaire : Parramatta Square / Parramatta City Council

Architectes : Tangram Lab, Tangram Architectes, Treex, Institut Méditerranéen d'Océnaologie

Coûts

Coût de construction : 2 500 €/m^2

Principaux prix

Sans objet

Certifications

Sans objet

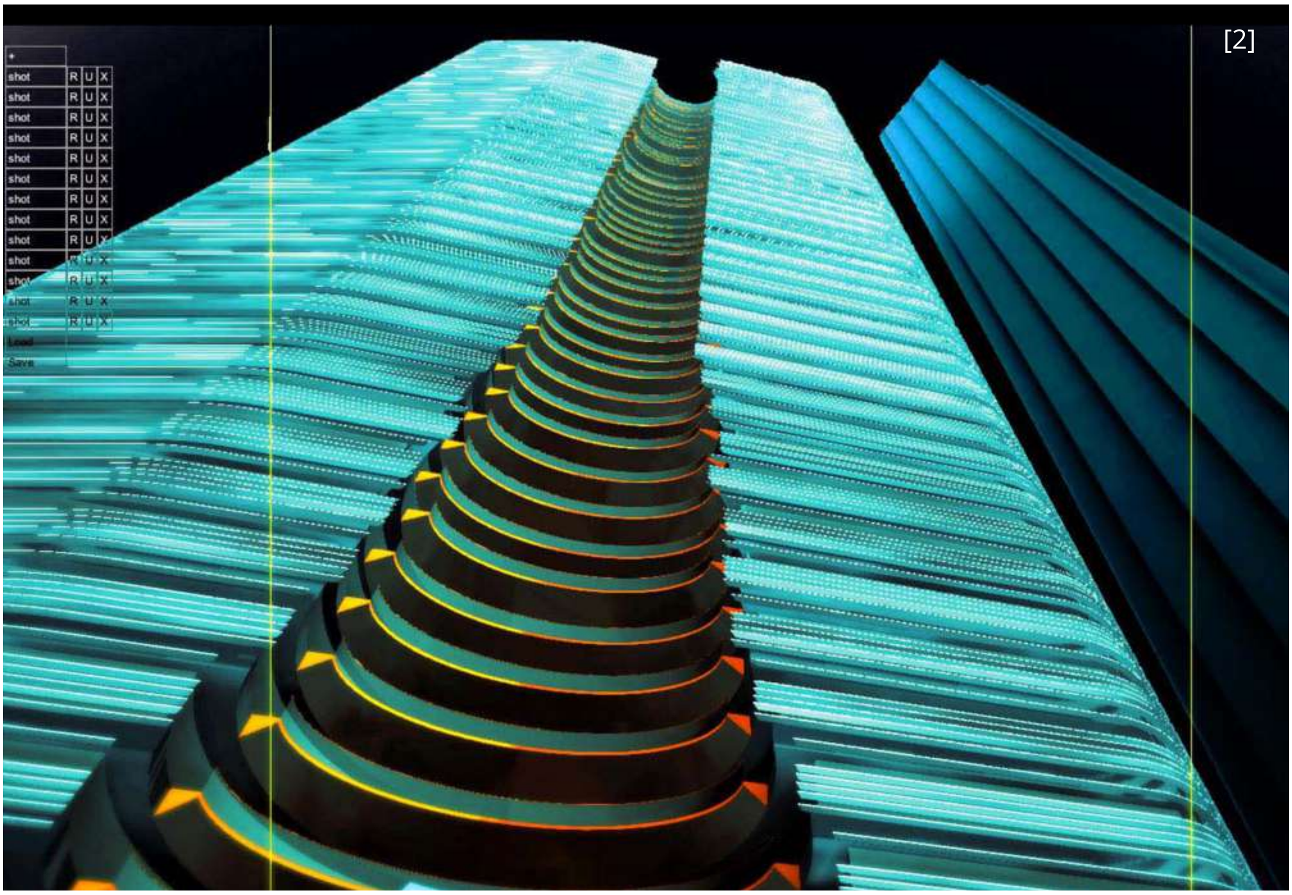

[2]

ANALYSE GLOBALE DU PROJET

Le projet joue le rôle d'interface urbaine. Il doit se connecter, d'un côté, à un pôle d'échange multimodal (trains, bus, parking enterré) et, de l'autre côté, à un large mail piéton reliant une ancienne église et de nombreux bâtiments institutionnels. L'enjeu est d'importance car le projet, dont le programme de 120 000 m² de commerces, se doit d'être le symbole d'une ville concurrençant Sydney sur le terrain de l'Innovation, de la connectivité, du digital et du travail. Le projet doit briller et affirmer sa présence 24/7.
Le terrain et la forme urbaine sont fortement contraints par le *master plan* et imposent deux tours dont les contours sont largement esquissés. Les critères de jugements sont très ambitieux et placent, par ordre hiérarchique, la durabilité, le design, le design urbain, la faisabilité et l'innovation.

Objectifs

L'objectif du projet est triple :

• **Réaliser une interface urbaine** entre l'accès aux tours, le pôle d'échange, les parkings enterrés, le grand mail piéton, tous à des niveaux différents, qui permettent une pratique de l'espace public riche et variée sans croisement de flux entre piétons et véhicules. Le sol a été modelé tel de grandes banquettes urbaines continues, serpentant pour relier l'ensemble des espaces à desservir. Les tours sont conçues comme un empilement de ces mêmes strates générant un ensemble continu.

• **Garantir des consommations raisonnables** eu égard au programme et aux surfaces demandées, particulièrement énergivores. Des planchers doubles à inertie ont été imaginés. Connectés à des prises d'air en façade et à une grande cheminée centrale (entre les 2 tours), ce système permet un chauffage/rafraîchissement passif saisonnier.

• **Conférer au projet une image dynamique, innovante et assurer un lien entre une culture aborigène millénaire et un renouveau** caractérisé par des architectures verticales de verre. Nous avons proposé un éclairage nocturne bioluminescent des tours pour briller naturellement tout en réduisant au maximum les consommations électriques.

Conception biomimétique

La raison d'une conception biomimétique réside dans le paradoxe apparent entre être énergétiquement vertueux et accueillir 120 000 m² de commerces, éclairés la nuit, avec des conditions de température et de ventilation contraignantes.

Les sources d'inspiration dans le vivant pour ce projet ont été :

• **Les anguilles tachetées de Parramatta :** emblèmes de la ville, cette espèce a inspiré la maîtrise d'œuvre. L'observation des nasses à anguilles, entonnoirs tressés, inventées par les aborigènes et des écailles sous-cutanées de ces poissons qui leur permettent de passer de l'eau douce à l'eau salée, a été source d'inspiration. En effet, ces observations ont amené à imaginer un système de cheminée centrale couplé à des planchers à inertie comprenant un vide d'air fermés par des écailles en façade. Ce système passif de régulation thermique du bâtiment permet une réduction de sa consommation électrique.

• **Les méduses :** la bioluminescence de cette espèce a conduit la maîtrise d'oeuvre à proposer un éclairage naturel peu énergivore répondant à la demande de signal nocturne. La lumière émise rappelle celle des anguilles ondulant dans les méandres de la rivière Parramatta.

Impacts documentés du projet

Les impacts potentiels visés sont :

• **Créer un lieu qui fluidifie les parcours** ; connecte différentes centralités et qui permet des usages variés ;

• **Contribuer à minimiser les consommations énergétiques** par un éclairage nocturne bioluminescent, ainsi que par des systèmes de ventilation et chauffage/rafraîchissement naturels passifs ;

• **Faire un pont entre la culture millénaire aborigène et la volonté d'innovation actuelle**.

Impacts sur la recherche

Ce projet a débouché sur un long processus de recherche et développement, toujours en cours, notamment par une thèse intitulée « Biolum-Archi, un phénomène biologique fascinant au service d'un modèle d'habitat durable méditerranéen ». Cette thèse a fait progresser la connaissance en étudiant les conditions de milieu et de croissance de bactéries naturellement bioluminescentes et a permi d'étudier différentes conditions de transfert au monde de la ville, du bâtiment et du paysage au travers de plusieurs partenariats en collaboration avec la recherche publique et privée. Un brevet est en cours de dépôt.

DÉMARCHE BIOMIMÉTIQUE

Approche
- [x] Problem driven
- [ ] Solution based

Origine de la bio-inspiration
- [x] Opportunité/hasard/intérêt personnel
- [ ] Collaborations interdisciplinaires
- [ ] Appel à projet
- [ ] Autre

Performances ciblées
- [x] Confort thermique
- [ ] Confort visuel/lumineux
- [ ] Confort acoustique
- [x] Qualité de l’air intérieur
- [ ] Qualité de l’air extérieur
- [ ] Résistance aux contraintes mécaniques
- [ ] Gestion de l’eau intérieur
- [ ] Gestion de l’eau extérieur
- [ ] Accueil de la biodiversité
- [x] Adaptation au climat
- [ ] Allègement de la structure
- [ ] Gestion des déchets
- [x] Autres

Échelle d'intégration
- [ ] Matériaux
- [x] Technologie
- [ ] Système façade/toit/sol
- [ ] Bâtiment
- [x] Îlot
- [ ] Quartier

PROCESSUS DE CONCEPTION

Utilisation d'un cadre de conception
- [x] Oui
- [ ] Non

Contraintes majeures
- [ ] Manque de fonds
- [ ] Utilisation d'outils biomimétiques
- [ ] Règlementations
- [x] Problèmes techniques
- [ ] Autres

Complexité de la conception
- [x] Haute
- [ ] Faible

LIEN AVEC LA BIOLOGIE

Niveau d'inspiration
- [x] Organisme
- [ ] Comportement
- [ ] Écosystème

Dimension de l'inspiration
- [ ] Forme
- [ ] Matériaux
- [ ] Constructions/structure
- [x] Processus
- [x] Fonction

Types de modèles inspirants
- [x] Animal : Anguille tachetée de Parramatta, Méduse lune
- [ ] Plante
- [ ] Champignon
- [x] Archea/Bacteria : *photobacterium phosphoreum*
- [ ] Protozoaire
- [ ] Chromista
- [ ] Écosystème

Type de connaissance utilisée
- [ ] Existantes pour le grand public
- [x] Existantes pour le spécialiste
- [x] Créées pendant la conception

Type d'apports en biologie
- [ ] Historique/savoir du concepteur
- [x] Acquisition lors de la conception
- [ ] Biologistes intégrés au processus

RÉSULTATS & INNOVATION

Complexité de la construction
- [x] Haute
- [ ] Faible

Niveau d'innovation
- [x] Innovation de rupture
- [ ] Amélioration de l'existant

LEÇONS À RETENIR

Les enseignements de ce projet résident dans la réponse à deux questions à priori antinomiques :

• Créer, sous la forme de deux tours de commerces de 120 000 m² de commerces, un signal symbolique fort à l'échelle de la ville de jour comme de nuit ;

• Être durable et vertueux.

La résolution de ce paradoxe s'est faite, au-delà des multiples connexions d'espaces publics entre elles, par une étude plus large du territoire et de ses habitants. La compréhension des fils cachés du site et de ses alentours au cours du temps a pu ouvrir de nouvelles perspectives à la maîtrise d'œuvre. La compréhension que les éléments naturels (la rivière Parramatta) étaient des liants bien plus puissants et durables que les infrastructures humaines récentes, a permis à la maîtrise d'œuvre d'ouvrir des pistes de recherches et réflexions relatives au biomimétisme urbain, afin de construire des infrastructures durables. Ce projet a amené la maîtrise d'œuvre à revoir son processus de conception architectural et urbain en y intégrant à part entière l'étude et la connaissance du vivant.

Crédits des images :

[1] [2] Treex + Tangram Architectes

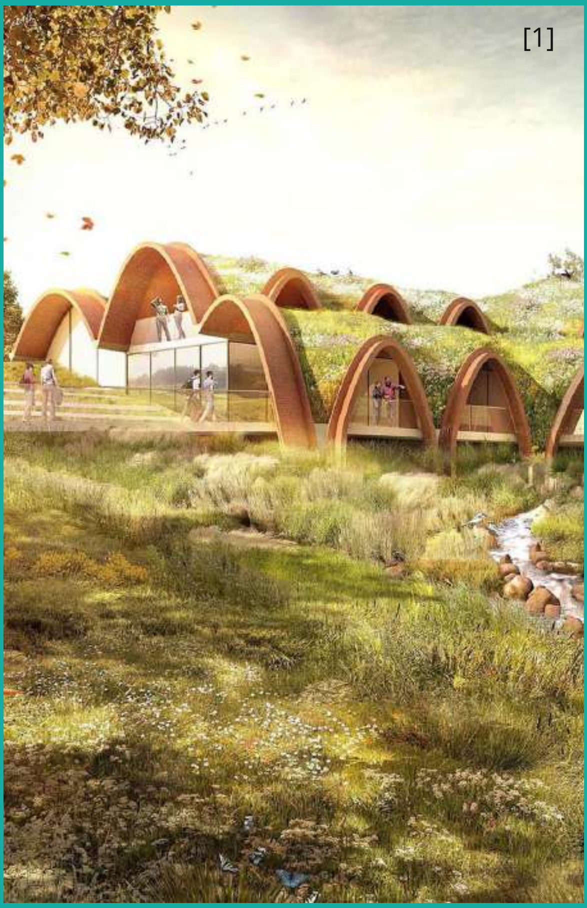
[1]

PÔLE D'EXCELLENCE SUR LE BIOMIMÉTISME MARIN

Adresse : Avenue de la Milady, 64200 Biarritz
Statut du projet : non accepté
Date de conception : 2019
Type/usage : centre de recherche
Rénovation : non
Climat : CFB - climat tempéré chaud, sans saison sèche et à été tempéré
Surface du projet : 2 194 m² SU, 2 300 m² d'emprise, 2 890m² SDP
Coût de construction : 2 760 €/m²
Coût final du projet : 8 M€

Membres du Biomim'City Lab engagés :

TANGRAM ARCHITECTES
Nobatek
INEF4

RÉSUMÉ

Avec pour ambition de structurer la filière Océan à l'échelle du Pays Basque, l'Agglomération du Pays Basque a choisi de bâtir un pôle d'excellence sur le biomimétisme marin qui se veut être un centre d'innovation et de recherche et un lieu de créativité autour de l'économie de l'océan. Situé à 600 mètres de l'océan, le mot d'ordre de la maîtrise d'ouvrage est l'exemplarité environnementale et la conception bio-inspirée. Pour concevoir ce bâtiment, dans un processus à la fois créatif et réaliste, en tenant compte des contraintes liées à sa construction et à son intégration dans un site naturel en bordure d'océan, l'équipe-projet s'est inspirée de la grotte sous-marine et du modèle biologique de l'éponge de mer.
Tourné vers l'Océan et dialoguant avec le grand paysage des Pyrénées, le Pôle d'Excellence sur le Biomimétisme Marin (PEBM) s'apparente davantage à un paysage construit qu'à un bâtiment paysager. Cette hybridation entre architecture et paysage est le résultat d'une conception biomimétique. Le PEBM entend rendre des services écosystémiques, être un bâtiment régénératif. Apportant des réponses aux 7 pétales et 20 impératifs du label Living Building Challenge, le PEBM se veut un bâtiment vivant. En absorbant, régulant le débit et filtrant l'eau, en créant les conditions d'une biodiversité positive, en respectant la topographie, limitant les déblais et les déchets et en limitant la pollution lumineuse nocturne, le PEBM assure la continuité de quatre trames colorées (verte, bleue, brune et noire) faisant de ce lieu hybride d'innovation ouverte, un hub de biodiversité profitable à tous. Cette démarche écosystémique à faible impact environnemental, frugal, *low-tech* et bas carbone, s'incarne dans une solution technique traditionnelle et innovante : des voûtes maçonnées de terre crue. En utilisant une ressource locale, le PEBM qualifie la Région Nouvelle Aquitaine par le développement d'une filière vertueuse et d'une économie bleue régionale. Toute la phase de conception a été validée par un passage au crible des services écosystémiques rendus au site, et des impératifs du Living Building Challenge, certification particulièrement exigeante en matière de construction durable.

Revenir à la liste des projets

Rôle des principaux participants

Maîtrise d'ouvrage : Communauté d'Agglomération du Pays Basque
Architectes : Tangram Architectes/Tangram Lab
Architectes associés : Ragueneau & Roux Architectes, Claret+Lebecq Architectes
Pôle environnement et études de faisabilité : NOBATEK/INEF4
Bureaux d'études : EGIS bâtiments Sud-Ouest (fluides, économie), Acoustb (acoustique), Elioth (structure), Nobatek/INEF4 (bioclimatisme + biomimétisme)
Conseil scientifique : Seaboost + M.I.O.

Coûts

Coût de construction : 2 760 €/m²
Coût final du projet : 8 M€

Principaux prix

Sans objet

Certifications

• LBC 3.0 utilisé comme cadre lors de la conception

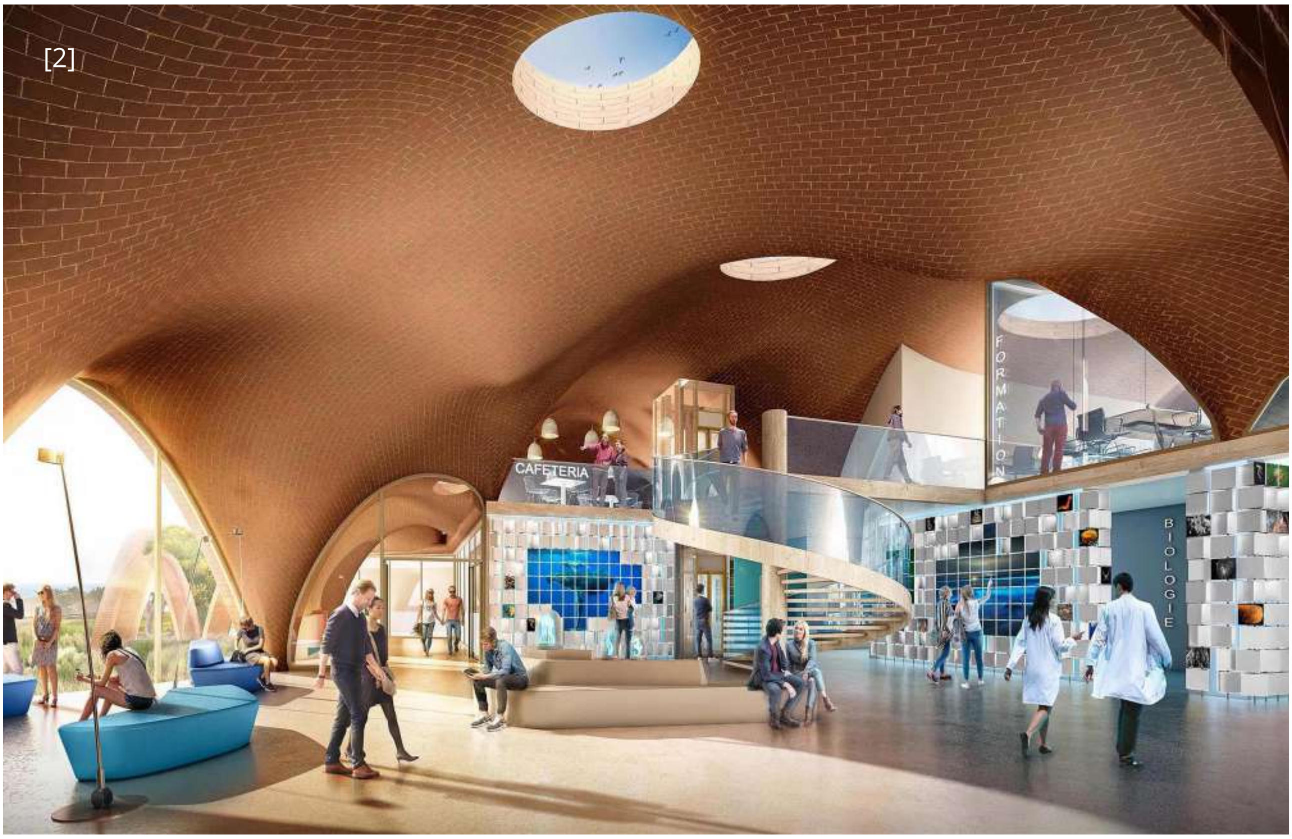

[2]

ANALYSE GLOBALE DU PROJET

La Communauté d'Agglomération du Pays basque a lancé en 2019 un dialogue compétitif pour l'attribution d'un marché de maîtrise d'œuvre pour le Pôle d'Excellence sur le Biomimétisme Marin à Biarritz. Ce futur lieu hybride d'innovation ouverte, de créativité et de recherche sur le biomimétisme marin et l'économie de l'océan, constitue une première nationale. À ce titre, le programme prévoit, dans un esprit collaboratif et ouvert, un centre de ressources, des laboratoires de recherche et des bureaux.

À l'image de sa thématique, le bâtiment devra être régénératif, résultant d'une conception biomimétique ou bio-inspirée et ouvert vers l'océan. Le site d'étude faisait partie autrefois d'une jonction hydraulique naturelle entre le lac de Mouriscot et l'Océan, une vaste zone humide et un réservoir de biodiversité de première importance. Aujourd'hui, ceint par des voies très empruntées le surplombant partiellement, notre site offre une poche non bâtie entre deux lotissements. La topographie marquée est accentuée par 2 *thalwegs* qui rappellent l'importance de sa fonction originelle de zone naturelle qu'il convient de restituer.

Ce projet a pour volonté de positionner le Pays basque comme référence en matière de biomimétisme marin, et de créer un bâtiment vecteur des activités scientifiques et économiques autour de l'économie bleue. Ambitionnant d'accueillir un pôle d'excellence en biomimétisme, ce bâtiment rassemblera divers acteurs du biomimétisme : l'IPREM Océan (Institut des sciences analytiques et de physico-chimie pour l'environnement et les matériaux), des espaces projets pour les étudiants du Master Matériaux bio-inspirés de l'UPPA, un centre de ressources en biomimétisme marin animé par Ceebios, ainsi qu'une pépinière d'entreprise. Afin de tendre vers un bâtiment régénératif, l'équipe-projet s'est confrontée aux exigences ambitieuses du Living Building Challenge, avec pour objectif d'être autonome en énergie et surtout en eau.

Objectifs

Pour répondre aux objectifs de bâtiment vivant, régénératif, à biodiversité positive et ainsi répondre aux objectifs de chaque pétale du LBC, les concepteurs ont proposé les solutions suivantes :

• **Le site :** Situé à quelques mètres seulement de l'iconique Cité de l'Océan, le bâtiment adopte une posture humble en épousant parfaitement la déclivité de 7 mètres grâce à sa structure en voûtes de terre crue successives. Le PEBM fait également face à un espace boisé compris dans le réservoir de biodiversité du Lac Mouriscot. Face à cette haute valeur environnementale, les concepteurs ont pris grand soin d'assurer un corridor écologique, à travers des structures d'accueil ou de passage (nichoirs, écoducs) ou une végétation autour et sur les toits adaptés aux espèces locales.

• **Contribuer à la qualité des paysages alentours :** en proposant une toiture vallonnée, à l'échelle du grand paysage et du bâti existant, directement connectée à 3 espaces naturels riches irrigués par 3 ruisseaux au plus près de la topographie existante. Créer ainsi une continuité totale de la trame verte.

• **S'insérer dans le tissu urbain existant :** en ne bloquant pas les vues, la lumière naturelle, en ne créant pas de nuisances sonores, olfactives, routières et en prenant en compte le réseau hydrographique à une échelle plus large. Recueillir, stocker, filtrer l'eau et la rejeter propre dans l'océan (trame bleue).

• **Participer au développement de l'économie bleue à l'échelle de la Région Nouvelle Aquitaine :** en agissant « local » et pensant « global » par l'emploi de voûtes maçonnées en terre crue afin de relier vernaculaire et innovation durable. Utiliser un minimum de matière pour supporter un maximum de terre végétale (trame brune) et s'inscrire dans une démarche frugale, *low-tech* et bas carbone.

• **La qualité de l'eau :** L'eau du thalweg et de pluie servent d'apport pour en améliorer sa qualité de sortie.

• **Tendre vers l'autonomie énergétique :** grâce à un principe de frugalité avec stratification d'air par les voûtes l'été et serres bioclimatiques l'hiver, ainsi que l'entrée de lumière naturelle au sein du bâtiment.

• **La bioluminescence :** Proposer un éclairage naturel bioluminescent adapté à la vie des espèces nocturnes (trame noire).

• **Organiser le programme avec un cœur, le centre de ressources, et 3 branches convergentes :** (laboratoires, tertiaire, fonctions) communes : le tout dans le but de travailler, échanger et innover au contact de la nature.

• **Une conception biophilique pour répondre à des enjeux sanitaires et de confort :** Cette conception biophilique offre un confort thermique grâce à une inertie thermique développée par à ses voûtes de terre crue et sa ventilation naturelle traversante et zénithale. La conception biophilique du bâtiment s'opère aussi à travers sa végétalisation et son dessin paysager.

Conception biomimétique

L'équipe projet s'est attachée à faire émerger des solutions adaptées au contexte, ainsi toute la phase de conception, de l'idéation au tri des idées, s'est faite au travers de boucles biomimétiques, qui ont été validées ou écartées selon les services écosystémiques et les impératifs du Living Building Challenge. Les mots clés du projet sont : frugalité, *low-tech*, biodiversité, écosystème local et faible impact écologique.

Les boucles biomimétiques se sont découpées en 4 grandes étapes :

1. Définition des problématiques du projet et exploration de plusieurs thématiques (climat, hydrologie, géologie, faune/flore, nuisances) ;

2. Recherche dans la donnée biologique de réponses existantes ;

3. Exploration et la compréhension de modèles biologiques inspirants puis leur sélection ;

4. Transfert des modèles vers des solutions technologiques à mettre en oeuvre, pour répondre aux problématiques liées aux quatre trames.

Dans le cadre des travaux de recherches effectués au sujet des boucles biomimétiques, 2 espèces vivantes et 1 élément géologique ont été retenus :

• **Les éponges de mer :** pour leur capacité à gérer l'eau et à favoriser la biodiversité ;

• **Les bactéries bioluminescentes :** pour une bio-utilisation favorable à la vie nocturne ;

• **Les grottes terrestres et sous-marines :** dans une démarche dite géo-mimétique, pour s'inspirer de l'ambiance qu'elles créent.

Les solutions suivantes sont issues du processus biomimétique :

· **Stocker, retenir et filtrer l'eau :** La continuité de la circulation de l'eau présente sur le site a été assurée ainsi qu'un rejet en aval d'une eau propre de polluants par un stockage en bassin, une lombrifiltration (eaux grises et noires) ainsi que la filtration des eaux de ruissellement (phytoépuration).

· Les éponges de mer source d'inspiration pour la **trame verte :** ces espèces structurantes favorisent le développement d'une biodiversité par mutualisme et commensalisme. L'implantation du bâtiment, son adaptation au substrat et les formes vallonnées de sa toiture créent différentes strates de biodiversité. Les formes générées sont résultantes d'une étude des espèces locales mises en danger par la raréfaction de leurs habitats naturels. Pour cela divers espaces d'accueil de la biodiversité seront créés;

· Bâtiment adapté à la déclivité du sol et **respect de la topographie**.

· **Des voûtes maçonnées inspirées des grottes :** s'intègrent au paysage par ses formes courbées et vallonnées adaptées aux contraintes topographiques du site.

· À l'instar des éponges de mer, fabricant leur squelette par ajouts successifs, le PEBM sera fabriqué à partir de **terre crue** : issues notamment des travaux de décaissement, par ajouts successifs.

· **Limitation des nuisances lumineuses nocturnes** par utilisation des bactéries bioluminescentes.

Revenir à la liste des projets

Impacts documentés du projet

• Ce bâtiment garantit une **continuité paysagère sol/toiture**, ses structures, son **corridor écologique**, et son traitement de l'eau.

• Il permet aussi de s'immerger dans un paysage riche **mettant en scène le chemin de l'eau** jusqu'à l'océan.

• Par ses **services écosystémiques** et son origine directement inspirée du vivant, il sensibilise le public au biomimétisme marin et à la conception à faible impact environnemental, tout en inspirant ses futurs occupants.

• S'inscrit dans le grand paysage (montagnes, océan) et crée une **continuité avec la Cité de l'Océan**.

• Fournit des **espaces extérieurs de détente variés** vivant au rythme des saisons et propices à la cohésion sociale en activant les 5 sens.

• **Améliore la situation des lotissements alentours** (lumière, vue, accès à la nature, gestion de l'eau).

Impacts sur la recherche

La proposition d'utiliser la bioluminescence s'appuie sur des travaux déjà existants : dans le cadre des travaux du Tangram Lab et du M.I.O (Mediterranean Institute of Oceanography), la thèse intitulée « Biolumarchi » se penche sur la bioluminescence appliquée à l'architecture, et vise à fournir un éclairage diffus nocturne, qui est durable et écologique. Tangram Architectes continue à ramifier ses recherches sur la bioluminescence. Par ailleurs, deux projets de Recherche & Développement ont été initiés à la suite de ce projet. Le premier, en partenariat avec Nobatek/INEF4, concerne 3 thématiques générales à savoir l'énergie/les économies d'énergie, la matière/les économies de matières et l'intégration du vivant dans le processus de conception. Le second, en partenariat avec Elioth, concerne la compréhension de la terre crue et le développement de cette filière. Depuis Tangram a gagné un projet dans lequel ce matériau sera utilisé.

DÉMARCHE BIOMIMÉTIQUE

Approche
- ● Problem driven
- ● Solution based

Origine de la bio-inspiration
- ● Opportunité/hasard/intérêt personnel
- ● Collaborations interdisciplinaires
- ● Appel à projet
- ○ Autre

Performances ciblées
- ● Confort thermique
- ● Confort visuel/lumineux
- ● Confort acoustique
- ● Qualité de l’air intérieur
- ○ Qualité de l’air extérieur
- ● Résistance aux contraintes mécaniques
- ● Gestion de l’eau intérieur
- ● Gestion de l’eau extérieur
- ● Accueil de la biodiversité
- ● Adaptation au climat
- ● Allègement de la structure
- ● Gestion des déchets
- ○ Autres

Échelle d'intégration
- ● Matériaux
- ● Technologie
- ● Système façade/toit/sol
- ● Bâtiment
- ○ Îlot
- ● Quartier

PROCESSUS DE CONCEPTION

Utilisation d'un cadre de conception
- ● Oui
- ○ Non

Contraintes majeures
- ○ Manque de fonds
- ● Utilisation d'outils biomimétiques
- ● Règlementations
- ● Problèmes techniques
- ○ Autres

Complexité de la conception
- ● Haute
- ○ Faible

LIEN AVEC LA BIOLOGIE

Niveau d'inspiration
- ● Organisme
- ● Comportement
- ● Écosystème

Dimension de l'inspiration
- ○ Forme
- ● Matériaux
- ● Constructions/structure
- ● Processus
- ● Fonction

Types de modèles inspirants
- ● Animal : Éponge de mer
- ● Plante
- ○ Champignon
- ○ Archea/Bacteria
- ○ Protozoaire
- ○ Chromista
- ● Écosystème

Type de connaissance utilisée
- ● Existantes pour le grand public
- ● Existantes pour le spécialiste
- ○ Créées pendant la conception

Type d'apports en biologie
- ● Historique/savoir du concepteur
- ○ Acquisition lors de la conception
- ● Biologistes intégrés au processus

RÉSULTATS & INNOVATION

Complexité de la construction
- ● Haute
- ○ Faible

Niveau d'innovation
- ● Innovation de rupture
- ○ Amélioration de l'existant

LEÇONS À RETENIR

Le PEBM invite à adopter un autre regard sur la conception de nos bâtiments. En s'inspirant de la certification Living Building Challenge le PEBM tend à être un bâtiment frugal qui n'utilise que peu de matériaux pour sa conception, un batiment low tech demandant la mise en œuvre de technologies simples ainsi qu'un bâtiment bas carbone visant à être régénératif.

Une étude complète à l'échelle du territoire a fait émerger des problématiques fortes sur la discontinuité des trames verte, brune et bleue alors même que le Pays-Basque est un des points chauds de la biodiversité au niveau mondial. Les contraintes fortes du site d'implantation (déclivité, implantation) ont fait émerger une opportunité de réunir techniques de construction vernaculaire et innovation durable valorisant une filière d'économie bleue à l'échelle de la Région Nouvelle Aquitaine. La recherche des espèces en danger localement a conduit l'équipe en charge de la conception du PEBM à formaliser les toitures du projet pour leur créer des habitats et des lieux propices à leur développement. La volonté de traiter naturellement le flux écologique de l'eau bien au-delà de notre terrain a guidé notre implantation. Ainsi, à l'image de notre modèle biologique qu'est l'éponge, le bâtiment se fond dans son environnement pour mieux se mettre à son service.

Le processus de conception biomimétique intégrant un conseil scientifique, a amené la maitrise d'œuvre à faire de très nombreuses itérations et à développer leurs connaissances sur de nombreux sujets. En retour, les connaissances non utilisées directement ont enrichi le processus permettant à la maitrise d'œuvre d'en améliorer les rouages.

Un dernier résultat très intéressant réside certainement dans le choix de l'espèce biologique dont la seule référence a permis l'équipe en charge de la conception du PEBM de répondre à de nombreuses contraintes.

Crédits des images :

[1] [2] SO-images

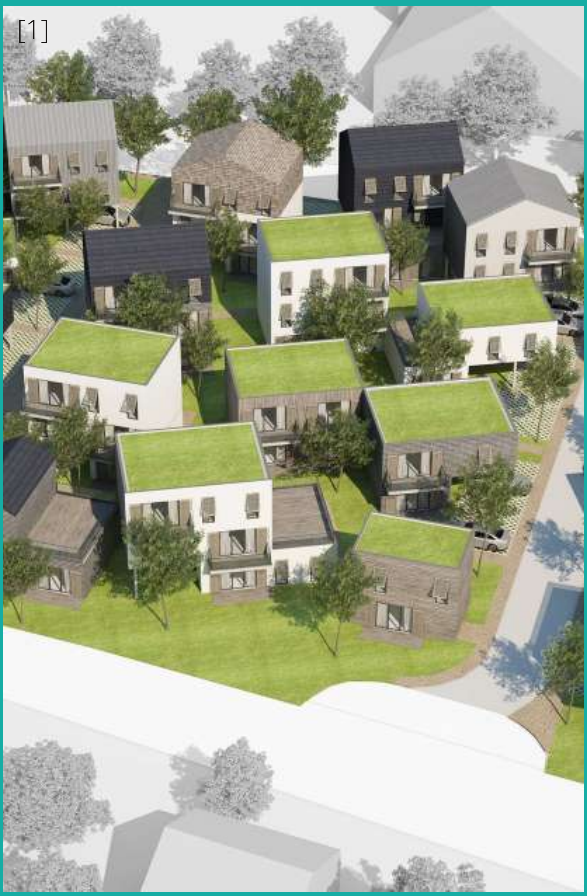

[1]

RÉSIDENCE SOLAIRE D'ORDENER

Adresse : Caserne Ordener, Senlis, France
Statut du projet : projet sans suite
Date de conception : 2018
Type/usage : logements
Rénovation : non
Climat : CFB - climat tempéré chaud, sans saison sèche et à été tempéré
Surface du projet : 3576 m^2 SHAB
Coût de construction : 5,88 M€ HT programme initial - avril 2018
Coût final du projet : 4.9 M€ HT programme bailleur social - septembre 2018

Membres du Biomim'City Lab engagés :

IN SITU ARCHITECTURE
Nobatek INEF4

RÉSUMÉ

Ce projet consiste à créer un ensemble de soixante-dix logements. L'implantation des bâtiments et leur orientation ont été calculées à l'aide d'un algorithme phyllotaxique solaire mis au point par In Situ architecture en partenariat avec le laboratoire de biologie végétale RDP. À l'instar des feuilles d'une plante, les appartements bénéficient tous d'un ensoleillement direct, le bénéfice est alors double, il permet de réduire de façon significative leur consommation énergétique et d'augmenter la santé des usagers.

Le projet atteint quatre heures de soleil direct par bâtiment ce qui permet de réduire de 25% la consommation du chauffage par rapport à un projet classique de haute qualité environnementale.

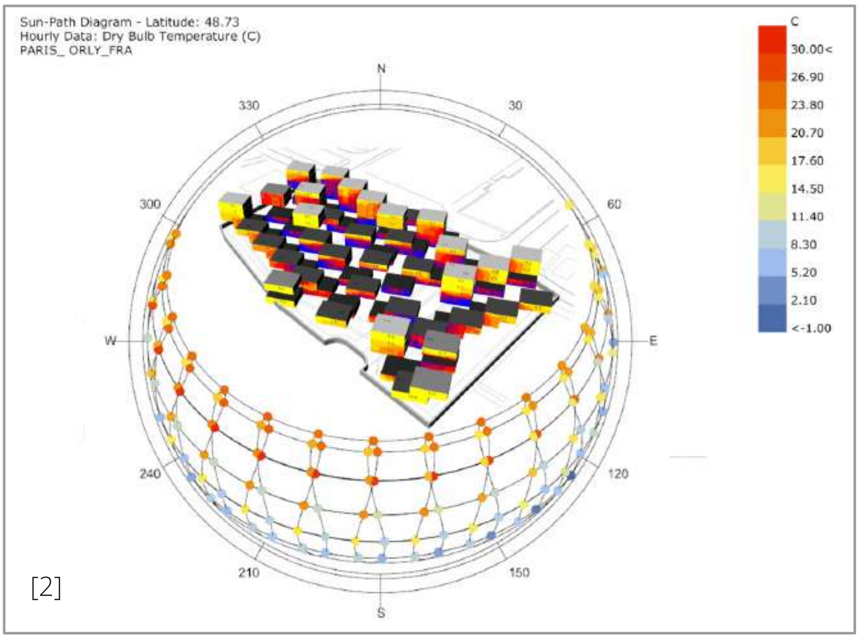

[2]

Rôle des principaux participants

Maîtrise d'ouvrage : IDéel délégué par la Mairie de Senlis
Architectes : In Situ Architecture et Nobatek-INEF4
BET TEC : Nobatek-INEF4

Coûts

Coût de construction : 5,88 M€ HT programme initial - avril 2018
Coût final du projet : 4.9 M€ HT programme bailleur social - septembre 2018

Principaux prix

Sans objet

Certifications

Sans objet

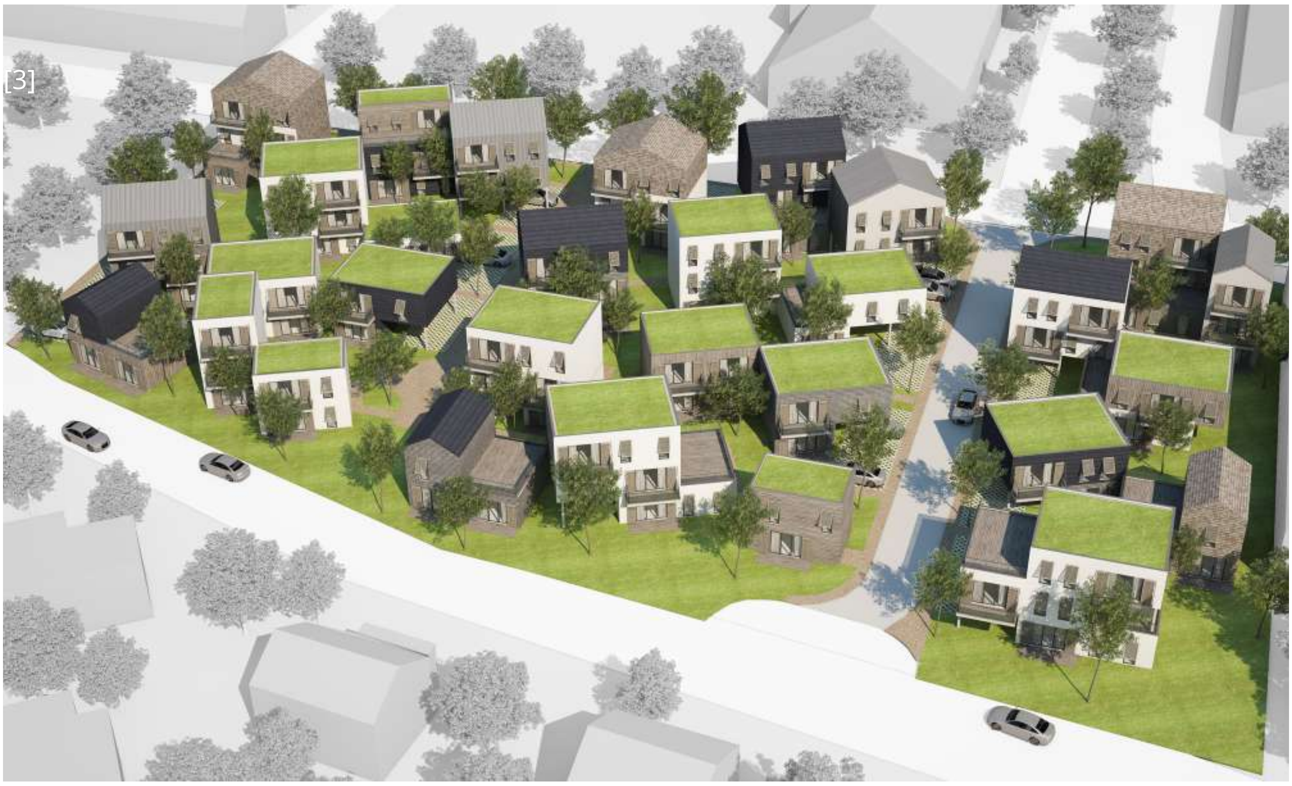

ANALYSE GLOBALE DU PROJET

L'appel d'offre de ce projet a été lancé en 2018, avec un cahier de charges pour la réalisation d'hébergements à destination de chercheurs et étudiants.
Le projet, avec un engagement fort pour l'économie d'énergie, a été laureat de l'appel d'offre, mais le programe a évolué au fil du temps. Du fait de changements d'acteurs, un nouveau projet est proposé à l'année 2018 mixant réhabilitation et construction de nouveaux bâtiments.

Objectifs

L'objectif principal du projet est la création d'une forte densité de logements économes en énergie.
Le projet propose d'utiliser les apports solaires passifs du soleil pour réduire les consommations des bâtiments. La création d'un algorithme bio-inspiré de la phyllotaxie des plantes permet de répondre à cet objectif principal et de maîtriser le rapport entre forte densité et apports solaires.

Le programme vise la création de 3516 m^2 SHAB pour une surface foncière disponible de 6333 m^2 hors voirie, le tout limité à une hauteur de deux étages avec toiture. Après plusieurs remaniements, le programme prévoit la création de soixante-dix logements sociaux de différentes tailles allant du studio à l'appartement 4 pièces (16 T1, 25 T2, 24T3, 5 T4). Les surfaces des logements étant normalisées par les bailleurs sociaux, le projet prévoit la création de jardins et de grandes terrasses extérieures afin de donner à chaque logement une pièce de vie supplémentaire. Cette configuration permet d'anticiper des usages mixtes type télétravail et de développer d'une approche biophilique. Enfin chaque logement bénéficie d'une place de parking extérieure directement accessible.

Le site est classé par les monuments historiques, les matériaux sont prédéfinis par la réglementation urbaines : toiture en tuiles et enduit en façade. Le projet propose d'élargir cette gamme de matériau à l'utilisation de bardeau de bois, zinc et toiture végétale.

Conception biomimétique

Avec 45% de l'énergie finale consommée et 25% des émissions de gaz à effet de serre (GES), le bâtiment est le plus gros gisement de la transition énergétique et environnementale. Bien que la construction neuve ne représente qu'1% du parc immobilier chaque année, les bâtiments neufs représentent une opportunité majeure de réduction des émissions de gaz à effet de serre. Plutôt qu'une approche à forte intensité technologique de la performance énergétique du bâtiment, nous proposons une approche d'ultra-passivité en optimisant tous les apports passifs possibles.
Alors qu'une plante est particulièrement vertueuse en terme de capture solaire, un bâtiment standard quant à lui utilise très peu les apports énergétiques du soleil. Il y a selon nous un véritable enjeu à explorer une morphogenèse de bâtiments « utra-passif » qui à l'instar des plantes capteront au maximum l'énergie du soleil. Le bénéfice est double, il permet de réduire de façon significative les consommations énergétiques en utilisant l'énergie passive du soleil et d'augmenter la bonne santé des usagers en régulant leur biorythme. La démarche ici est transversale entre biologie, mathématique, architecture bioclimatique et architecture paramétrique et elle réunit les compétences de chercheurs, d'ingénieurs et d'architectes.

Impacts documentés du projet

L'utilisation de l'énergie passive du soleil permet de réguler thermiquement un bâtiment et de diminuer sa consommation énergétique. Nous avons couplé nos études de cas (voir plus loin) avec une STD (simulation thermique dynamique) et nous sommes arrivés aux résultats suivants :

• 6 heures d'exposition directe (projet de 12 appartements à Senlis) permettent **50 % d'économie de chauffage** ; 4 heures d'exposition directes (projet de 70 appartements à Ordener) permettent **25 % d'économie de chauffage**.

Bien que plus difficilement quantifiable, les bénéfices santé, d'après la littérature scientifique porte sur :

• Une **meilleure régulation des rythmes circadiens** par les apports de lumière naturelle (régulation de la vitamine D, régulation de la mélatonine, diminution des SAD – Seasonal Affective Disorder).

• Une **diminution du stress** par les vues optimisées sur un cadre naturel, voire un sentiment d'appartenance à un lieu reconnecté aux principes du vivant (biophilie).

Impacts sur la recherche

Le travail de R&D sur la modélisation d'algorithmes phyllotaxiques a démarré en 2017. Ce projet a permis de tester son application et d'enrichir le programme de R&D en cours. En 2019, le programme a obtenu une subvention de la part de BPI et devrait passer en phase « développement » en 2021.

DÉMARCHE BIOMIMÉTIQUE

Approche
- ● Problem driven
- ○ Solution based

Origine de la bio-inspiration
- ● Opportunité/hasard/intérêt personnel
- ● Collaborations interdisciplinaires
- ● Appel à projet
- ○ Autre

Performances ciblées
- ● Confort thermique
- ● Confort visuel/lumineux
- ○ Confort acoustique
- ○ Qualité de l'air intérieur
- ○ Qualité de l'air extérieur
- ○ Résistance aux contraintes mécaniques
- ○ Gestion de l'eau intérieur
- ● Gestion de l'eau extérieur
- ● Accueil de la biodiversité
- ● Adaptation au climat
- ○ Allègement de la structure
- ● Gestion des déchets
- ○ Autres

Échelle d'intégration
- ○ Matériaux
- ○ Technologie
- ○ Système façade/toit/sol
- ● Bâtiment
- ● Îlot
- ● Quartier

PROCESSUS DE CONCEPTION

Utilisation d'un cadre de conception
- ● Oui
- ○ Non

Contraintes majeures
- ○ Manque de fonds
- ● Utilisation d'outils biomimétiques
- ● Règlementations
- ● Problèmes techniques
- ○ Autres

Complexité de la conception
- ● Haute
- ○ Faible

LIEN AVEC LA BIOLOGIE

Niveau d'inspiration
- ● Organisme
- ○ Comportement
- ○ Écosystème

Dimension de l'inspiration
- ● Forme
- ○ Matériaux
- ○ Constructions/structure
- ○ Processus
- ○ Fonction

Types de modèles inspirants
- ○ Animal
- ● Plante
- ○ Champignon
- ○ Archea/Bacteria
- ○ Protozoaire
- ○ Chromista
- ○ Écosystème

Type de connaissance utilisée
- ○ Existantes pour le grand public
- ● Existantes pour le spécialiste
- ○ Créées pendant la conception

Type d'apports en biologie
- ○ Historique/savoir du concepteur
- ○ Acquisition lors de la conception
- ● Biologistes intégrés au processus

RÉSULTATS & INNOVATION

Complexité de la construction
- ● Haute
- ○ Faible

Niveau d'innovation
- ● Innovation de rupture
- ○ Amélioration de l'existant

Revenir à la liste des projets

LEÇONS À RETENIR

Le résultat du calcul algorithmique phyllotaxique ne dessine pas un projet architectural, mais plutôt un ensemble de gabarits et d'implantations permettant la genèse d'un projet architectural qui pourra ainsi optimiser la capture de l'énergie solaire. Cet outil permet de développer une méthodologie d'optimisation de plans masses (implantations, volumétries, orientations et ouvertures des bâtiments d'un projet urbain) en phase amont de faisabilité architecturale ou urbaine. Ce projet est le plus avancé an terme de définition de projet et il montre que le calcul algorithmique permet une expression architecturale tout à fait singulière.

Crédits des images :

[1] [2] [3] In Situ Architecture

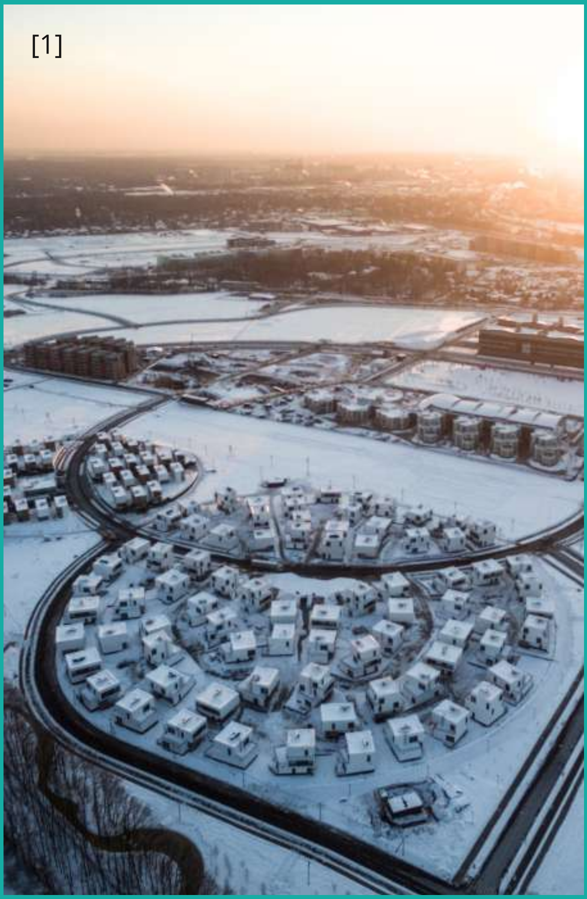
[1]

SKOLKOVO INNOVATION CENTER

Adresse : Skolkovo Innovation Center - District 11, Russie
Statut du projet : construit
Date de conception : 2012
Type/usage : logements
Rénovation : non
Climat : DFC - climat continental froid, sans saison sèche à été court et frais
Surface du projet : 17 000 m^2
Coût de construction : 556 €/m^2
Coût final du projet : 25 959 000 €

Membres du Biomim'City Lab engagés :

bechu + associés

RÉSUMÉ

Début 2010, le Président russe Dimitri Medvedev annonçait la création, dans la banlieue sud de Moscou, d'un vaste centre d'innovation technologique capable à terme de rivaliser avec la Silicon Valley californienne. Imaginé à l'image de la fameuse Cité des Étoiles de l'ex-Union soviétique transposée dans l'univers de toutes les technologies de pointe, ce complexe comprend : des laboratoires de recherche privés et universitaires, des entreprises et des start-ups, des pôles de rencontres et de séminaires, ainsi que des zones résidentielles. À l'issue d'un concours remporté en 2011, chaque quartier a été confié à un architecte de renommée internationale différent. L'agence d'architecture Bechu & Associés a remporté le district 11, quartier destiné à héberger les chercheurs et leurs familles. Afin de concevoir ce nouveau quartier, les architectes ont expérimenté une nouvelle approche de l'urbanisme écologique. Le district 11 est un quartier constitué de maisons familiales individuelles ayant pour objectif d'offrir un cadre de vie quotidien propice aux interactions sociales. Pour répondre à cet objectif l'agence d'architecture Bechu & Associés, s'est inspirée de l'organisation sociale des manchots sur la banquise. Afin de se tenir mutuellement chaud les pingouins se rassemblent en cercles concentriques. En se serrant les uns contre les autres, en groupes très denses (8 à 10 manchots au m^2), et en ne présentant que le haut de leur dos au vent froid, les manchots limitent les déperditions de chaleur. Ainsi, cette organisation sociale a été transposée aux dessins de l'agence, eux-mêmes façonnées par l'apport du design paramétrique via l'intégration de la data récuperée auprès des biologistes avec lesquels l'équipe a travaillé. Ce mode organisationnel, issu de la nature, projette une centaine de villas, regroupées dix par dix, dans une vaste clairière, ceinturée par un cours d'eau permettant d'évacuer la fonte des neiges. Ce regroupement en « tortue » qui a inspiré le plan de masse permet d'économiser 5°C de température par effet de radiation, au sein de chaque îlot. Ce projet est suivi et mesuré scientifiquement chaque année, confirmant ainsi l'efficience et l'intérêt de cette démarche biomimétique.

Revenir à la liste des projets

Rôle des principaux participants

Maîtrise d'ouvrage : OOO DAS SKOLKOVO
Architectes : Agence d'Architecture A. Bechu & Associés (Architecte mandataire)
Chef de projet : Pablo Lorenzino /// Daniel Kaufman (consultant développement durable)

Coûts

Coût de construction : 556 €/m²
Coût final du projet : 25 959 000 €

Principaux prix

2019 • Shortlisté au Green Solutions Award mention spéciale dans la catégorie « Sustainable Districts Grand Prizes »
2015 • Green Awards
2014 • Prix de l'Excellence remis par la municipalité de Shanghai - Global Smart City Best practice pour le projet de Skolkovo (Russie)
• 1er prix du Green Project « Ecofriendly Environment »

Certifications

• LEED

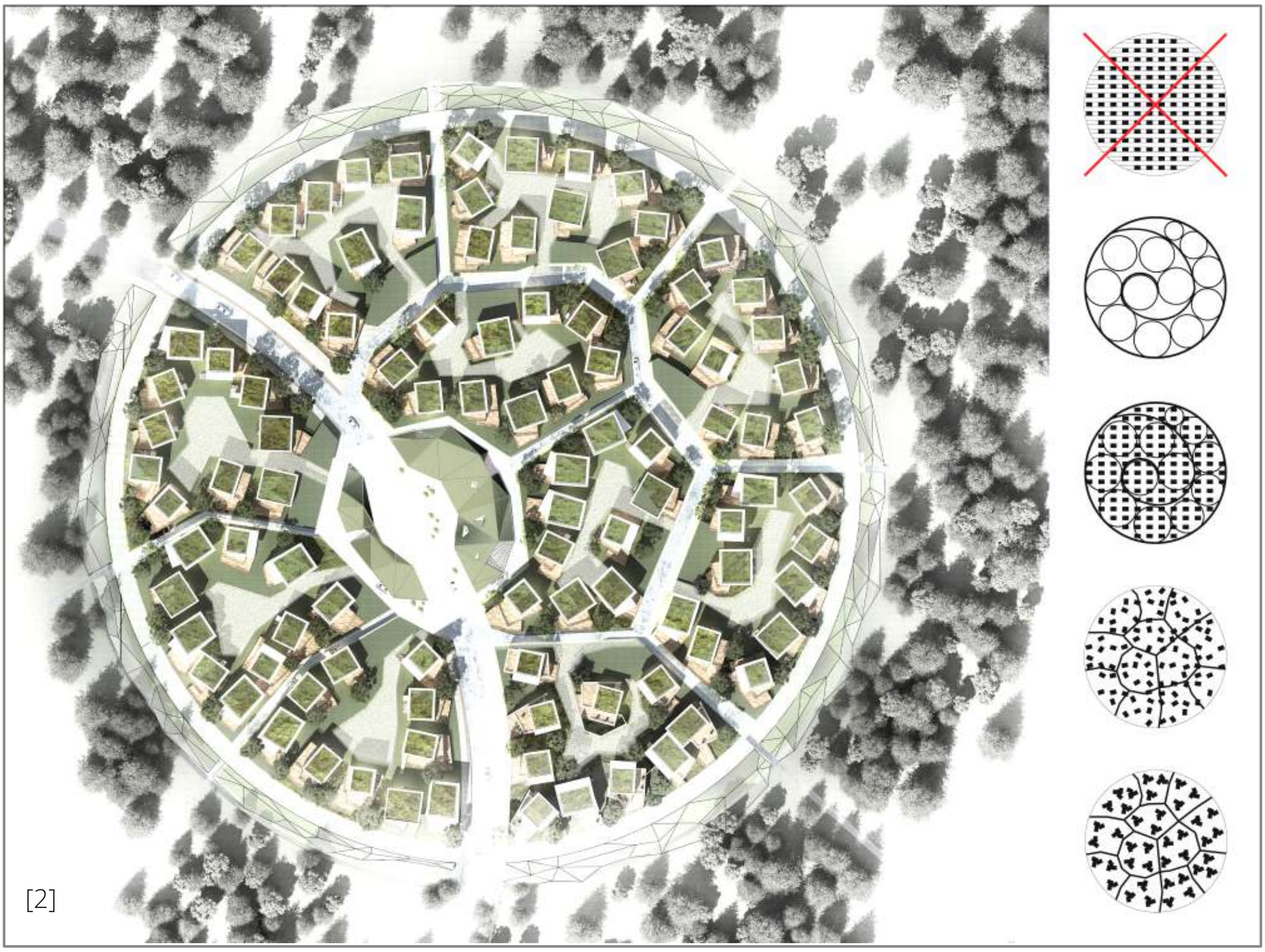

[2]

ANALYSE GLOBALE DU PROJET

Début 2010, le Président russe Dimitri Medvedev annonçait la création, dans la banlieue sud de Moscou, d'un vaste centre d'innovation technologique capable à terme de rivaliser avec la Silicon Valley californienne. Imaginé à l'image de la fameuse Cité des Étoiles de l'ex-Union soviétique transposée dans l'univers de toutes les technologies de pointe, ce complexe comprend des laboratoires de recherche privés et universitaires, des entreprises et des start-ups, des pôles de rencontres et de séminaires ainsi que des zones résidentielles.

Sur la base d'un schéma directeur global, chaque quartier aves ses spécificités programmatiques a fait l'objet d'un concours international. Bechu & Associés a remporté le district 11 destiné à y accueillir des maisons individuelles pour les chercheurs et leurs familles, à organiser pour que ce quartier facilite les échanges et permette des usages « doux » et partagés, dans un climat complexe très froid en hiver et chaud en été, où l'architecture doit faire le lien entre la nature et les personnes qui y vivent.

Ce projet est suivi et mesuré scientifiquement chaque année, confirmant ainsi l'efficience et l'intérêt de cette démarche biomimétique.

Objectifs

Le défi majeur de ce projet est de développer un quartier paysager et urbain optimal, esthétique et fonctionnel, et ce face à un climat extrême. Selon les années, les températures oscillent, entre hiver et été, de -42°C à +37°C, et la neige est présente en grande quantité durant plusieurs mois de l'année.

La spirale, figure présente dans la nature, qui régit l'organisation sociale des manchots, est dans ce projet devenu le modèle du village. Elle assure une protection physique aux maisons et aux habitants d'un quartier que l'on peut qualifier d'océan forestier.

Grâce à cette approche, dont l'optimisation du dessin s'est faite par la combinaison de données sur des outils paramétriques, le quartier peut s'adapter aux conditions climatiques tout en enrichissant les possibilités d'usage, de confort et d'interaction. Le quartier devient un « bio réseau » qui protège, abrite et offre de multiples possibilités d'espaces extérieurs.

Le quartier est organisé autour d'une place centrale et traversé par une rue de liaison, au sein de laquelle les activités quotidiennes s'organisent. Tout autour, des maisons individuelles carrées sont aménagées en spirale dans de petits groupes, *clusters*. Il en résulte un modèle de village compact qui préserve l'individuel et le caractère intime de chaque logement. L'approche bioclimatique résultante du plan directeur permet de maximiser les gains solaires au sein de chaque maison et groupe, tout en fournissant une protection aux vents.

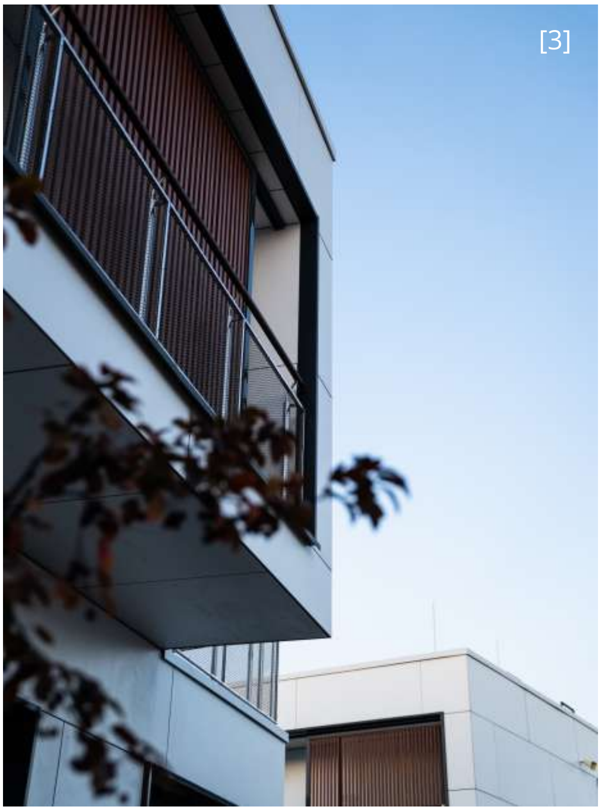
[3]

Conception biomimétique

• **Pingouins :** l'observation des manchots, se serrant les uns contre les autres en pivotant pour rester au chaud durant les tempêtes, a aidé la conception du plan directeur de l'agence d'architecture Bechu.

• **Bioclimatisme :** Le schéma directeur prend en compte la trajectoire solaire afin de maximiser l'apport solaire dans chaque maison et *cluster*, permettant ainsi de profiter de cours ensoleillées. La façon dont les maisons sont orientées, ayant toujours une façade froide et chaude, permet une ventilation naturelle pendant les journées chaudes d'été et de mi-saison, assurant le confort des occupants sans besoin de climatisation. La compacité des maisons permet une performance optimale de l'enveloppe, des écrans solaires adaptés complètent cette approche.

• **Toitures végétalisées :** Les toits verts contribuent au confort extérieur et à la réduction de l'effet d'îlot de chaleur par évapotranspiration, les nombreuses plantes contribuent à rafraîchir l'air et à limiter la dispersion des poussières.

Impacts documentés du projet

• La **consommation totale d'énergie primaire** est inférieure à 120 kWh/m² an (chauffage compris > 15 kWh /m²/an) ;

• **Isolation renforcée** : U entre 0,1 et 0,15 W / (m²K), environ 30 cm sur les parois verticales, 50 cm sous les toits ;

• **Suppression des ponts thermiques** grâce à l'isolation extérieure ;

• **Bonne étanchéité à l'air** (n50 inférieur à 0,6 h-1), membrane d'étanchéité à l'air continue verticalement et horizontalement ;

• Fenêtre à **triple vitrage**, fenêtres en U inférieures à 0,8 W / (m²K) ;

• Ventilation double flux avec **récupération de chaleur**, avec une batterie de chauffage couplée au chauffage urbain ou un échangeur géothermique ;

• Eau chaude via un **réseau de chauffage urbain local**.

Impacts sur la recherche

Ce projet a été rendu possible grâce aux convictions partagées avec le maître d'ouvrage, qui finance l'opération, mais aussi et surtout grâce à une collaboration multidisciplinaire. Cette œuvre est le résultat d'un travail commun réalisé avec des scientifiques, ingénieurs et des bureaux d'études spécialisés en environnement, structure et outils paramétriques. Pour chaque projet l'agence met en place des équipes aux compétences complémentaires pour fixer ensemble des objectifs structurels, récupérer les données nécessaires et générer les algorithmes qui participent à façonner ses dessins. Dans le cadre de la réalisation de ce projet l'agence d'architecture Bechu a travaillé avec son conseiller écologue, Daniel Kaufman, ainsi qu'avec des étudiants en biologie de l'université locale. Grace à ces étudiants l'agence Bechu a pu affiner sa compréhension de l'organisation des manchots.

DÉMARCHE BIOMIMÉTIQUE

Approche
○ Problem driven
● Solution based

Origine de la bio-inspiration
● Opportunité/hasard/intérêt personnel
○ Collaborations interdisciplinaires
○ Appel à projet
○ Autre

Performances ciblées
● Confort thermique
● Confort visuel/lumineux
○ Confort acoustique
● Qualité de l'air intérieur
● Qualité de l'air extérieur
○ Résistance aux contraintes mécaniques
○ Gestion de l'eau intérieur
● Gestion de l'eau extérieur
● Accueil de la biodiversité
● Adaptation au climat
○ Allègement de la structure
● Gestion des déchets
○ Autres

Échelle d'intégration
○ Matériaux
○ Technologie
○ Système façade/toit/sol
○ Bâtiment
○ Îlot
● Quartier

PROCESSUS DE CONCEPTION

Utilisation d'un cadre de conception
● Oui
○ Non

Contraintes majeures
● Manque de fonds
○ Utilisation d'outils biomimétiques
○ Règlementations
○ Problèmes techniques
○ Autres

Complexité de la conception
○ Haute
● Faible

LIEN AVEC LA BIOLOGIE

Niveau d'inspiration
○ Organisme
● Comportement
○ Écosystème

Dimension de l'inspiration
○ Forme
○ Matériaux
○ Constructions/structure
● Processus
● Fonction

Types de modèles inspirants
● Animal
○ Plante
○ Champignon
○ Archea/Bacteria
○ Protozoaire
○ Chromista
● Écosystème

Type de connaissance utilisée
● Existantes pour le grand public
● Existantes pour le spécialiste
○ Créées pendant la conception

Type d'apports en biologie
○ Historique/savoir du concepteur
○ Acquisition lors de la conception
● Biologistes intégrés au processus

RÉSULTATS & INNOVATION

Complexité de la construction
○ Haute
● Faible

Niveau d'innovation
○ Innovation de rupture
● Amélioration de l'existant

Revenir à la liste des projets

LEÇONS À RETENIR

Les conditions de vie aux pôles sont rudes : basses températures, présence de glaces terrestres et de mer, alternance marquée jour/nuit et faibles taux d’humidité. Les espèces vivantes qui s’y sont adaptées ont développé tout un arsenal de réponses morphologiques, physiologiques et comportementales, qui inspirent la technologie. Le succès de ce concept a aussi été dépend de l’acceptation des futurs habitants ; car la sensibilisation et l'éducation sont essentielles pour atteindre les objectifs de durabilité à long terme. Enfin, le biomimétisme montre sa capacité à s'exprimer à l'échelle urbaine. L'étude des écosystèmes — l'interconnexion entre espèces animales et végétales —, infuse la conception urbaine.

Crédits des images :

[1] [2] [3] © Agence d'Architecture A. Bechu & Associés

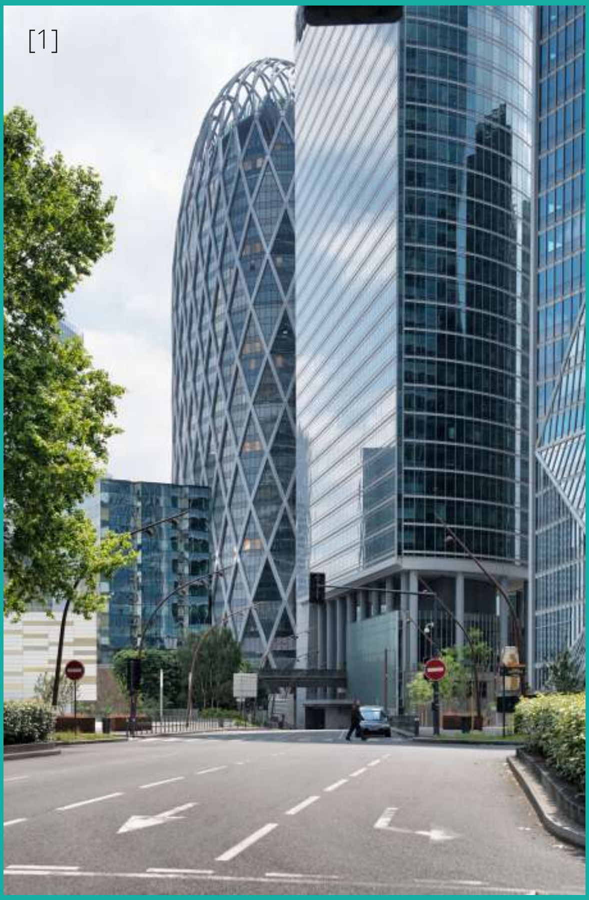
[1]

TOUR D2

Adresse : Paris La Défense - France
Statut du projet : construit
Date de conception : 2007
Type/usage : tertiaire
Rénovation : non
Climat : CFB - climat tempéré chaud, sans saison sèche et à été tempéré
Surface du projet : 54 500 m²
Coût de construction : 2 900 €/m²
Coût final du projet : 160 M€

Membres du Biomim'City Lab engagés :

bechu + associés

RÉSUMÉ

Concours gagné en 2007, projet livré fin 2014 par l'agence d'architecture Bechu en association avec Tom Sheehan, la tour D2 est l'un des symboles du renouveau de La Défense. Parce qu'elle est une composante du projet urbain qui se développe dans le quartier d'affaires, la tour D2 crée un lien vivant entre l'esplanade de La Défense et le quartier mitoyen de Courbevoie. Elle se dresse ainsi sur le boulevard circulaire, qu'elle contribue à transformer en boulevard urbain et humain. Bâtiment novateur, la coiffe de la tour D2 est habitée et accessible. Elle abrite le « Jardin des Nuages », un jardin à ciel ouvert de 500 m² qui culmine à 170 mètres d'altitude. Un bâtiment désormais emblématique à La Défense, dont la conception a permis l'économie de 30% de matière.

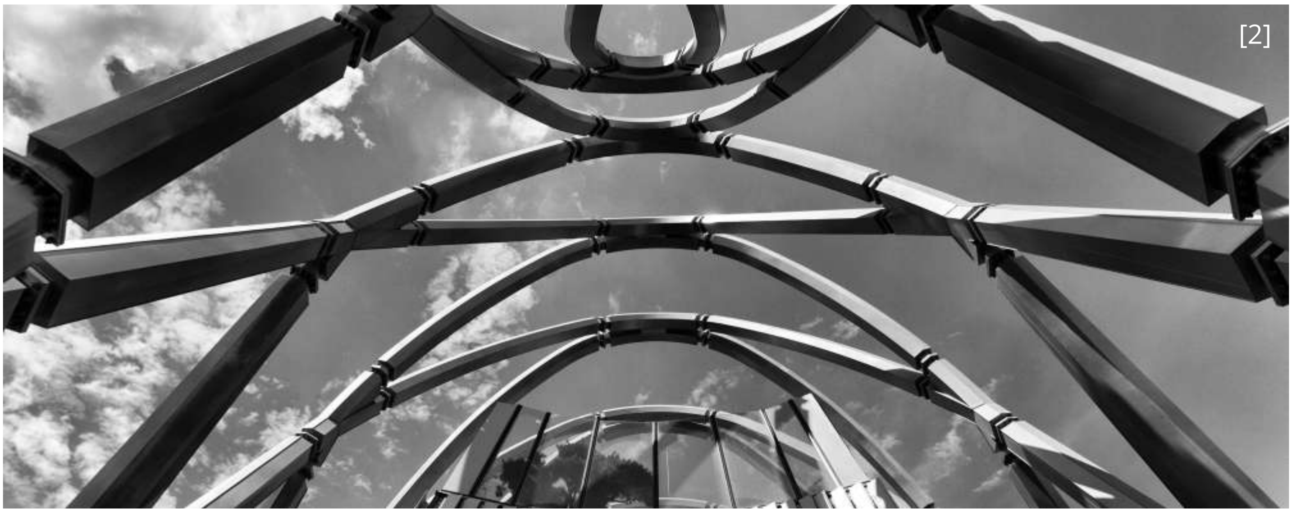
[2]

Rôle des principaux participants

Commanditaire : SOGECAP
Maître d'ouvrage délégué : Sogeprom et Bouygues Immobilier
Architectes : Agence d'Architecture A. Bechu & Associés (chef de projet : Régis Lassausse, associé) en association avec Tom Sheehan
BET façades : DVVD
Architecture d'intérieur : Volume ABC
Développement durable : Étamine

Coûts

Coût de construction : 2 900 €/m²
Coût final du projet : 160 M€

Principaux prix obtenus

2015
- Trophée Eiffel
- Prix Emporis Skyscrapers - troisième place
- Trophée du Bien-Être, catégorie « Habitat et Environnement »
- Archi Design Club Awards 2015, Catégorie « Bureaux et Commerces »

Certifications obtenues

- WELL
- BREEAM
- RT 2012
- NF HQE

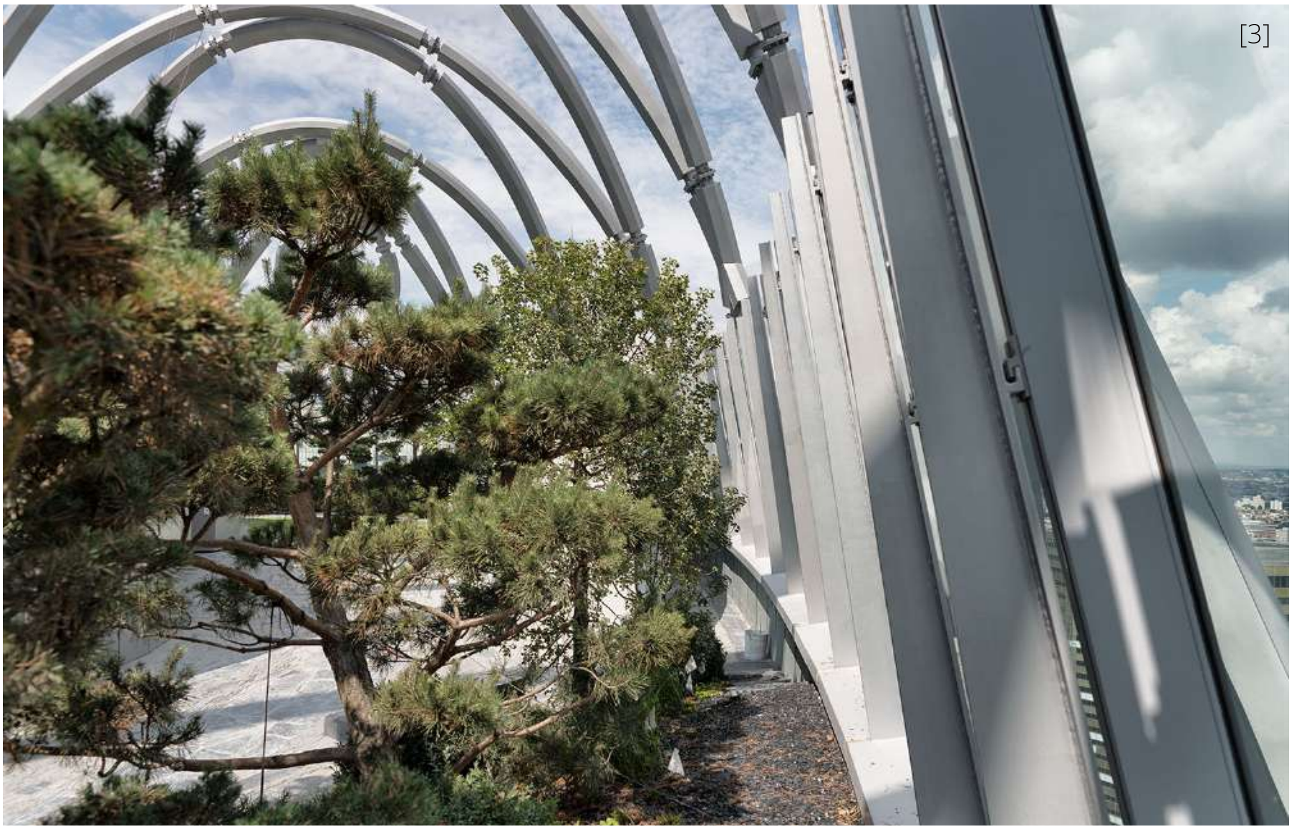
[3]

ANALYSE GLOBALE DU PROJET

La construction de la Tour D2 s'inscrit dans le cadre du plan de renouveau du quartier d'affaire de La Défense avec la volonté de l'aménageur et de la ville de Courbevoie de créer un quartier attractif, autant par les surfaces proposées que par les performances environnementales de l'ensemble des bâtiments.
Le terrain, propriété de Sogecap, se situe sur le boulevard périphérique, entre la Seine et la Grande Arche, et accueillait initialement les bureaux de Bureaux Veritas au sein d'un bâtiment vétuste de 10 000 m², que la tour D2 a donc remplacé. La tour de 37 niveaux comprend principalement des étages de bureaux livrés en blanc (35 000 m²), mais également des salles de réunion, un espace *fitness* et détente, ainsi que des restaurants inter-entreprises et un jardin en toiture. La tour D2 est un symbole fort de ce quartier d'affaire parisien puisqu'elle qui marque l'entrée dans le quartier de La Défense lorsque l'on vient de Courbevoie au nord, et qui participe également à recoudre un lien piéton entre l'esplanade de La Défense et la ville.

Objectifs

Édifiée à 171 mètres de hauteur, cette tour est la première de La Défense à avoir une exo-structure qui libère l'emprise de son noyau en béton. Englobant chacun des six étages de bureaux, les losanges de son exosquelette d'acier habillé d'aluminium – unique à La Défense – se rétrécissent pour engendrer une coupole ouverte inattendue où un jardin aérien évoluera dans les nuages. Économisant près de 30 % de matière, ce stratagème structurel épargne à la tour D2 la traditionnelle morphologie tripartite (socle, corps, couronnement) des tours voisines. En effet, la forme ovoïde qui l'en distingue minimise non seulement les masques visuels vis-à-vis de son voisinage immédiat, mais suscite également une perception dynamique de la part des piétons et des automobilistes. Si les tours voisines ne s'y reflètent pas, elle se réfléchit pourtant bien dans chacune d'entre elles ! Développée par autosimilarité, la géométrie de cette nasse métallique oblongue est d'essence fractale au même titre que l'arborescence de son noyau béton – concentrant les circulations (ascenseurs, escaliers, fluides...) – d'où rayonnent, telles des branches, les poutrelles raidissant de l'exosquelette. Les vitrages, eux, viennent s'y sertir tel des facettes d'un diamant ou de feuilles dont les multiples orientations ménagent l'ensoleillement tout en cadrant les vues sur la capitale, Courbevoie, ainsi que La Défense.

Conception biomimétique

• **Tour inspirée du périoste :** C'est l'observation de la structure du périoste (membrane fibreuse entourant les os) qui a inspiré cette exo-structure. Cette particularité lui permet d'alléger sa masse. En s'inspirant du périoste ainsi que d'autres exo-structures très présentes dans le monde vivant, l'agence d'architecture Bechu & Associés a réfléchi à la possibilité d'utiliser cet avantage structurel à l'échelle du bâtiment.

• **Atteindre la compacité optimale :** La tour D2 présente une forme quasi optimale en matière de compacité, puisqu'il s'agit en plan d'une forme ovoïde, avec un noyau central et des plateaux de bureaux en périphérie. La surface de façades est donc, à surface au sol équivalente, plus faible que dans le cas d'une tour à base carrée ou rectangulaire. La performance thermique de la tour en est donc meilleure.

• **L'originalité dans le principe structurel adopté :** notamment par le choix d'une exo-structure en acier, plutôt qu'une structure classique en béton. Cette exo-structure donne une grande flexibilité au projet, et permet une construction très préfabriquée. La structure se décompose en mailles, en forme de losanges verticaux. La hauteur des mailles, a été optimisée à 6 niveaux de manière à diminuer le coût de la structure et à limiter les linéaires de ponts thermiques au niveau des cadres et des ossatures.

• **Un plancher constituant le branchage :** Les planchers ont été pensés comme les branches primaires d'un arbre, prenant leur source sur le tronc, noyau central de l'édifice. Le sommet de la tour, tel un jardin dans les nuages est quant à lui inspiré de la canopée, étage supérieur de la forêt.

Impacts documentés du projet

• **Les ponts thermiques** d'accroche des cadres de la façade au niveau de l'exo-structure ont été travaillés de manière à réduire les déperditions au maximum. Le taux de surface vitrée de la façade est limité à 35% en vitrages, et 50% en intégrant les cadres de menuiseries des vitrages et des éléments de remplissage. Le tout étant associé à une isolation renforcée des parois donnant sur l'extérieur, la performance globale de la façade est de Ucw = 1,22 W/m²K.

• **La solution structurale** developpée a méné à une réduction de 30% en termes de matériaux de construction.

• **Une démarche à la fois biomimétique et biophilique :** pour ce projet, l'agence d'architecture Bechu & Associés a travaillé à mi-chemin entre biophilie et biomimétisme. Outre l'exo-structure, au sommet de la tour, à 171 mètres de hauteur, le « jardin des nuages », fait de celle-ci une allégorie de l'arbre, et un îlot urbain de biodiversité, puisque des oiseaux sont même venus y faire leur nid.

Impacts sur la recherche

Sur ce projet, la démarche biomimétique a été principalement partagée avec le BET DVVD, avec lequel l'agence a déjà travaillé sur des structures organiques complexes. Ensemble, ces deux acteurs de la maîtrise d'œuvre ont suivi une véritable démarche de R&D pour affiner leur dessin dans l'objectif d'économiser un maximum de matière, et réduire ainsi l'empreinte carbone de la future tour. Partageant leurs recherches avec le constructeur Vinci, ils ont dessiné ensemble près de 30 noyaux, avant d'aboutir à une structure satisfaisante.

DÉMARCHE BIOMIMÉTIQUE

Approche
- ● Problem driven
- ○ Solution based

Origine de la bio-inspiration
- ○ Opportunité/hasard/intérêt personnel
- ● Collaborations interdisciplinaires
- ○ Appel à projet
- ● Autre

Performances ciblées
- ● Confort thermique
- ● Confort visuel/lumineux
- ○ Confort acoustique
- ○ Qualité de l’air intérieur
- ○ Qualité de l’air extérieur
- ● Résistance aux contraintes mécaniques
- ○ Gestion de l’eau intérieur
- ○ Gestion de l’eau extérieur
- ● Accueil de la biodiversité
- ○ Adaptation au climat
- ● Allègement de la structure
- ○ Gestion des déchets
- ○ Autres

Échelle d'intégration
- ○ Matériaux
- ○ Technologie
- ● Système façade/toit/sol
- ○ Bâtiment
- ○ Îlot
- ○ Quartier

PROCESSUS DE CONCEPTION

Utilisation d'un cadre de conception
- ● Oui
- ○ Non

Contraintes majeures
- ○ Manque de fonds
- ○ Utilisation d'outils biomimétiques
- ○ Règlementations
- ● Problèmes techniques
- ○ Autres

Complexité de la conception
- ● Haute
- ○ Faible

LIEN AVEC LA BIOLOGIE

Niveau d'inspiration
- ● Organisme
- ○ Comportement
- ○ Écosystème

Dimension de l'inspiration
- ● Forme
- ○ Matériaux
- ● Constructions/structure
- ○ Processus
- ○ Fonction

Types de modèles inspirants
- ● Animal
- ○ Plante
- ○ Champignon
- ○ Archea/Bacteria
- ○ Protozoaire
- ○ Chromista
- ○ Écosystème

Type de connaissance utilisée
- ● Existantes pour le grand public
- ● Existantes pour le spécialiste
- ○ Créées pendant la conception

Type d'apports en biologie
- ○ Historique/savoir du concepteur
- ● Acquisition lors de la conception
- ○ Biologistes intégrés au processus

RÉSULTATS & INNOVATION

Complexité de la construction
- ● Haute
- ○ Faible

Niveau d'innovation
- ○ Innovation de rupture
- ● Amélioration de l'existant

LEÇONS À RETENIR

Ce projet a particulièrement démontré que le temps passé à faire des études ne représente pas un coût, mais un réel investissement, quand on en voit ici les effets positifs : un coût cible et un calendrier respecté. La tour a même été livrée avec 3 mois d'avance ! Aujourd'hui près de 7 ans après sa livraison, la tour D2 est une icône qui se démarque par son aspect organique et sa silhouette qu'aucune autre n'a. Elle crée véritablement le renouveau du paysage de La Défense parce qu'elle fait écho au vivant.

Crédits des images :

[1] [2] [3] © Pierre Elie de Pibrac

Merci de votre lecture

Ce document est voué à évoluer
dans le temps, à l'image de ce collectif.
Si vous souhaitez participer à la prochaine édition,
ainsi que rejoindre le Biomim'City Lab,
contactez-nous.

À bientôt

contact@ceebios.com

Illustrations type "topographiques" : Freepik
Police utilisée : Open sans

Ceebios

Biomim'
CITY
LAB

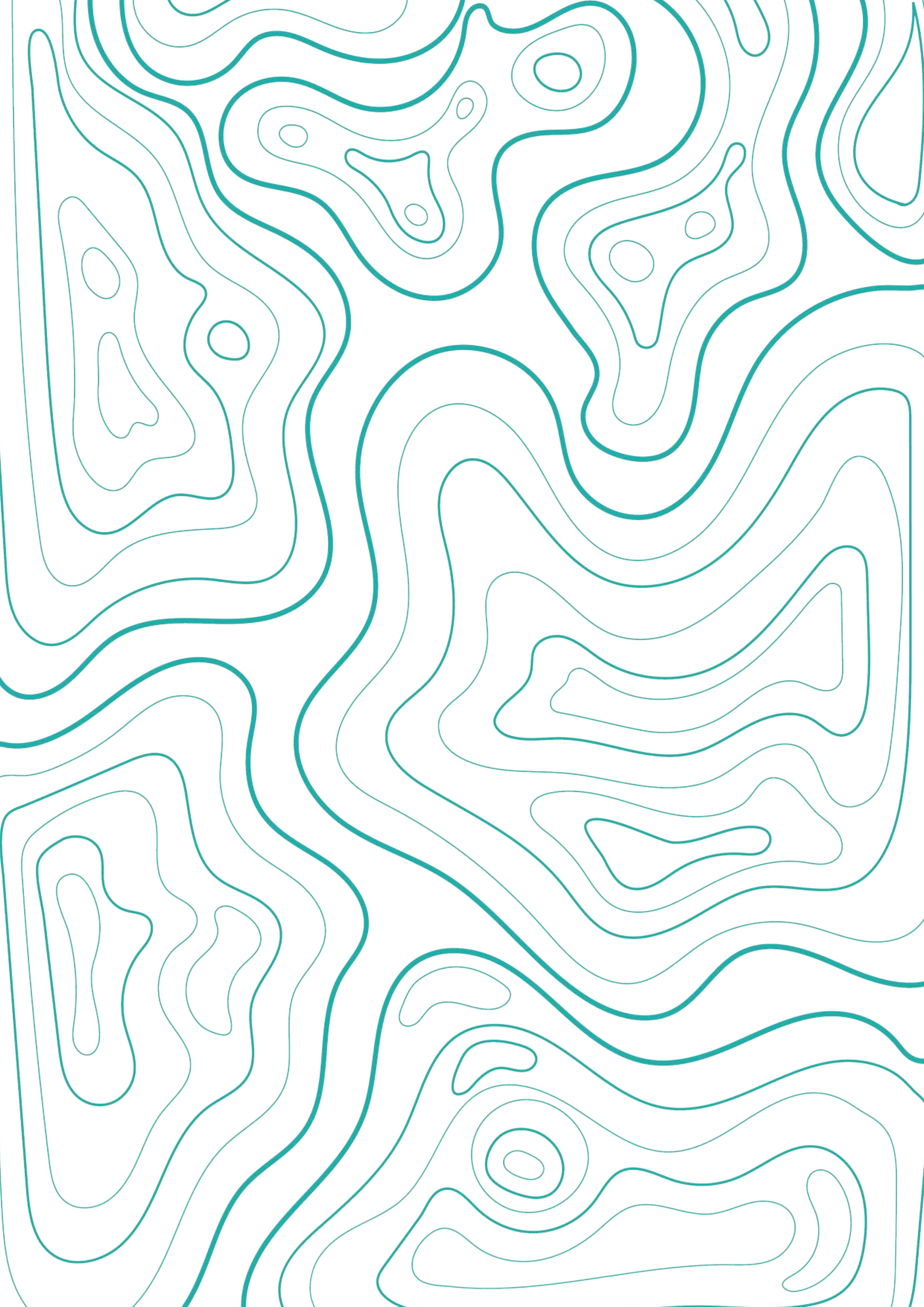

Supplement: Supplementary file 1 [file biomimetics-06-00027-s001.zip › S3 - Original source document -EtatDesLieux-BCL-20201223-web-BasDef.pdf]
