# Supplementary material for: Biomimicry in French Urban Projects: Trends and Perspectives from the Practice
Source: Biomimetics (Basel). 2021 Apr 27;6(2):27. doi: 10.3390/biomimetics6020027 (PMC8167731; doi:10.3390/biomimetics6020027)
Supplement: Supplementary file 1 [file biomimetics-06-00027-s001.zip › biomimetics-1144442-si.pdf]

Variables and classification options definitions (Table 2)

Year of design: Project design year

Project status: Actual project status.

Accepted and constructed or under construction: Projects accepted by project owners and constructed or under construction.

Accepted but not constructed: Projects accepted by project owners but not built.

Not Accepted: Projects not accepted by project owners.

Housing: Projects that mainly aims to provide new housing units.

Equipment: Projects that receive public and offer benefits to a broad population, like schools, hospitals, churches, and other sport and cultural projects.

Office: Projects that aim commercial or business use, as offices and stores.

Mixed-use: Projects with a mix of different uses, without a predominant one.

Renovation

Partial renovation on the project: The project has some level of retrofitting on existing infrastructures.

No renovation : The project does not have any retrofitting.

Aimed objectives with the biomimetic approach: objectives aimed by designers with the biomimetic approach

Thermal comfort: Biomimicry was used to improve the thermal comfort of the project.

Visual/lighting comfort: Biomimicry was used to address the project's visual and/or lighting comfort.

Acoustic comfort: Biomimicry was used to address the acoustic comfort of the project.

Indoor air quality: Biomimicry was used to promote better indoor air quality.

Outdoor air quality: Biomimicry was used to promote better outdoor air quality.

Resistance to mechanical stress: Biomimicry was used to face structural challenges and reduce structural charges.

Indoor water management: Biomimicry was used to manage water used inside the building as potable and wastewater.

Outdoor water management: Biomimicry was used to manage rain and stormwater.

Biodiversity hosting: Biomimicry was used to promote a better accommodation of biodiversity in the project, promoting habitat offer.

Adaptation to climate change: Biomimicry was used to face the new urban challenges related to climate change, promoting adaptability to new climatic conditions.

Lightening of the structure: Biomimicry was used to reduce the overall structure weight.

Waste management: Biomimicry was used to promote waste management solutions.

Others: Other objectives that do not fit on the above classifications (not specified).

Integration level of the biomimetic approach: Different project levels in which biomimicry was applied.

Materials: Biomimicry was applied on building construction materials.

Technology: Biomimicry was applied on specific building support technologies (as waste treatment and decontaminations systems)

Façade/roof/floor system: Biomimcry was applied most at the envelope level, affecting the façade, roof and or floor.

Building: Biomimicry was applied at the building scale, affecting its form, structure and other main characteristics.

Plot/Neighbourhood: Biomimicry was applied beyond the building scale, affecting the urban area and land use design at the plot or neighbourhood scale.

Type of biological model: Macro classification of the biological models

Eukaryotes – Animals

Eukaryotes – Plants

Eukaryotes – Fungi
Archea/Bacteria
Ecosystems

Aimed labels and certifications families: Sustainable labels aimed or achieved by the projects

Batiment passif
BDM
BiodiverCity
BREEAM
Cradle to Cradle
E+C-
Effinergie
HQE
LBC
LEED
Matériaux biosourcés
Nature-Art-Education
NF Habitat
RT2012
WELL

Lessons learned: Text excerpts from each project factsheet stating the lessons learned with the project design.
