# Supplementary material for: Biomimicry in French Urban Projects: Trends and Perspectives from the Practice
Source: Biomimetics (Basel). 2021 Apr 27;6(2):27. doi: 10.3390/biomimetics6020027 (PMC8167731; doi:10.3390/biomimetics6020027)
Supplement: Supplementary file 1 [file biomimetics-06-00027-s001.zip › S2 - Textual excerpts - export lecons.pdf]

Orginial textual excerpts analysed on MaxQDA ProAnalytics 2020 – Lessons learned

Alguesesns

La bio-façade intégrée au sein de l'Aglo House a été soumise à des tests d'acceptabilité sociale avant d'être déployée . En effet, la culture verticale d'algue au sein des façades de nos bâtiments n'est pas chose courante dans nos villes. Il a donc fallu, au préalable, s'assurer que les citoyens soient favorables à l'émergence de tels dispositifs.

• Le projet Alguesens et le projet de bio-façade Symbio2 ont vu le jour grâce à une collaboration entre des acteurs pluridisciplinaires. Ce projet urbain met ainsi en avant l'intérêt de mener des projets d'innovation collaboratifs afin d'en faire des projets fonctionnels, biomimétiques et assurant une certaine mixité sociale.

• Alors que nos villes doivent faire face à une pression démographique galopante et une urbanisation dense, le projet Alguesens assure la réintégration du vivant au sein de nos écosystèmes urbains.

Bangkok I'm Fashion Hub

Les enseignements de ce projet résident dans la conception d'une surface minimale composite bambou (tissage) / acier (pièces d'assemblage) qui répond à des paramètres variés telles que la distribution ordonnée des fonctions du programme, la connexion d'espaces publics à différentes altimétries et l'intégration de l'eau et des plantations dans l'architecture.

Le projet réfléchit aussi à la mise en place d'un cycle énergétique vertueux en réponse à un climat et à une industrie textile contraignants.

Cette réponse permet d'abolir la frontière entre toiture et façade, de penser l'enveloppe du bâtiment comme un tout unifié multifonctionnel permettant de traîter la protection solaire, le rafraîchissement et la ventilation naturels passifs. Cela ouvre la voie à un autre rapport dedans/dehors, intérieur/extérieur.

Biolum_Reef

La prise en compte des problématiques issues de l'activité anthropique, pesant sur les systèmes vivants, permet à la maîtrise d'œuvre d'apporter des solutions vertueuses ayant un impact à une échelle bien plus vaste que celle du site lui-même. Ceci incite à réfléchir de façon écosystémique, à prendre en compte les déchets, l'énergie, les matériaux ou la biodiversité comme des éléments d'un système global, non comme des éléments séparés.

De cette manière, un acte local peut avoir un puissant effet global. Ce projet a été une réelle prise de conscience sur l'impact positif que peut avoir un projet si l'on dépasse le seul intérêt humain présent dans un programme classique. Dès lors, l'étude et la compréhension des

principes du vivant deviennent un outil de conception puissant. Cela influe sur les outils, les méthodes de conception, la pluridisciplinarité et, in fine, sur la forme architecturale qui s'adapte aux multiples fonctions qu'elle remplit en interaction avec son environnement. Les limites de ce projet sont le nombre de projets de recherche associés qu'il faudrait lancer sur le long terme.

CIRC Lyon

Un processus de recherche et développement au cœur des solutions biomimétiques : les façades bio-inspirées du projet ont été objet d'un projet de recherche, avec plusieurs prototypes et tests avant son intégration dans le projet. À la suite de cette première application et aux recherches menées pendant les études par Art&Build, des évolutions futures sont envisageables.

En ce qui concerne la solution de verre thermochromique, les températures d'activation, les degrés de transparence et les couleurs sont des variables à ajuster en fonction du contexte spécifique de chaque nouveau projet, tout comme la répartition de ces éléments sur les façades en fonction de leur contexte spécifique. Des simplifications seraient possibles, par exemple avec le rajout d'une couche supplémentaire sur le verre ou en remplaçant le verre avec d'autres matériaux transparents comme le PMMA afin de faciliter la mise en œuvre. La connaissance de données météorologiques et un approvisionnement en énergie PV pourrait permettre une activation maitrisée du système pour anticiper des périodes de fort apport calorifique.

Quartier de Gally (Cité Fertile - Terres de Versailles)

Le projet Terres de Versailles a permis ICADE et l'équipe de conception de pousser les notions d'intégration entre ville et nature avec son projet de cité fertile, avec une approche biomimétique à l'échelle des écosystèmes.

L'intégration des acteurs engagés dans la transition écologique et le label Nature-Art-Education ont été des élements clés dans le succès du projet et dans sa performance écologique.

Ecoquartier Smartseille

Ce projet participe à la reconversion d'un ancien site industriel en un écoquartier en mettant notamment en œuvre des techniques de dépollution des sols par mycorémédiation et en y implémentant une dynamique sociale. Ce projet invite Eiffage à innover en termes d'urbanisme, d'éco-conception, de mobilité, d'habitat et d'aménagement des espaces créés, afin de participer à la construction de la ville durable de demain.

Ecotone

Écotone est un des premiers projets immobiliers parisiens à initier une démarche de biomimétisme en 2017. Malgré le contexte sanitaire de 2020, il s'agit d'un projet dont les enjeux sont de conserver ces ambitions architecturales et environnementales. Le temps long de la conception permet de continuer de développer la démarche de biomimétisme. L'importance de l'intégration du biomimétisme dès la phase concours avec une acculturation des architectes et de la maîtrise d'ouvrage en amont du projet est un élément clé. La volonté de la maitrise d'ouvrage est une condition sine qua non pour la réalisation d'un projet biomimétique. La création d'un comité scientifique pour accompagner et challenger l'équipe projet dès la phase concours est une action peu commune dans un process de promotion immobilière. Cette intégration de scientifiques experts en biomimétisme et biodiversité dès la phase concours est également une des clés de réussite du projet.

Eglise de Nianing

Élaborer un projet de ce type demande un certain niveau de maturation. Il existe aujourd’hui une incompatibilité temporelle entre le temps de conception lors d’un concours et le temps de recherche et développement. Pour autant, la phase de recherche est nécessaire afin de construire des projets urbains biomimétiques.

Contrairement aux programmes de recherches et développement nécessitant un investissement de temps sur le long terme, la phase de conception lors des concours ne dure que quelques mois. Développer un programme de recherche nécessite ainsi la création de partenariat et une curiosité à toute épreuve.

Estran

Utiliser les ressources locales pour répondre aux besoins du projet (aux différents services écosystémiques) est un enjeu majeur. Parfois difficilement réalisable. La notion de coût de construction arrive généralement dans le processus de conception et oblige à réduire les ambitions.

Les propositions innovantes mais générant un changement économico-socio-culturel importants sont parfois un frein malgré une volonté initiale forte de bâtiment régénératif. Dans le cadre d’un projet de construction d’un bâtiment régénératif, il semble important de pouvoir moduler la programmation de manière à ce que les usages puissent s’équilibrer les uns par rapport aux autres.

Groupe Scolaire des Sciences et de la Biodiversité

• Construire non pas une structure végétalisée mais vivante : il n’est pas rare que les façades et toits végétalisés aménagés sur nos bâtiments urbains soient de grands consommateurs d’eau

et d'énergie. Le Groupe Scolaire des Sciences & de la Biodiversité prouve qu'il est possible d'ériger un mur habité et une toiture végétalisée ne demandent que très peu d'entretien.

• Faire du bâtiment un outil pédagogique : le toit végétalisé du bâtiment un riche support pédologique pour les enseignants de l'établissement.

• Constituer une équipe de conception interdisciplinaire : architectes, écologues, paysagistes, etc. ont apporté leurs connaissances et compétences afin de bâtir une école accueillant une biodiversité endémique et s'intégrant dans son écosystème urbain.

• Imaginer un bâtiment inachevé : inachevé le mur d'enceinte est amené à évoluer dans le temps, favorisant ainsi l'accueil d'une nouvelle faune et flore spontanées.

• Conception des blocs de béton : Afin de faire du mur un espace d'accueil de la biodiversité il a fallu réfléchir aux différents types de moulage des blocs de béton, ainsi que leur positionnement au sein de la structure afin que les failles, trous et cavités soient adaptées à la faune et flore locales, capables de résister aux pressions environnementales.

Osez Joséphine

Ce projet a été l'occasion d'une démarche pédagogique d'explications de l'approche biomimétique et de son potentiel auprès de la maitrise d'ouvrage. Celle-ci en a compris les opportunités réelles que l'outil biomimétique confère en matière d'innovation low tech, tout en constatant par ailleurs quelques blocages inhérents à l'ensemble de la chaîne de la construction pas ou peu éduquée au sujet. Les études menées en équipe avec la direction technique ont rapidement permis de convaincre de la capacité du projet à répondre aussi à un équilibre financier global.

Ce projet appuie la raison d'être de la démarche et de l'action du Biomim'City Lab, nécessaire à la mise en place d'une méthode éducative et intelligible par l'ensemble des acteurs de la « fabrique des territoires ».

N'ayant pas été lauréat de l'appel à projets, ce sujet précis ne verra pas le jour. Il a cependant donné naissance à une nouvelle famille de bâtiments de logements, qui en cette période de révolution écologique peuvent être conçus autrement, malgré les règlementations très cadrées concernant ce secteur.

L'ensemble des travaux de recherche menés par l'agence Bechu & Associés sur la stratégie phyllotaxique des plantes a permis d'implémenter leur outil de design paramétrique, et de faire naître ainsi un algorithme qui transpose l'approche architecturale bioclimatique à l'échelle de l'appartement, pour générer un confort d'été et un confort d'hiver sans apport de technologie complémentaire.

Parramata

Les enseignements de ce projet résident dans la réponse à deux questions à priori antinomiques :

• Créer, sous la forme de deux tours de commerces de 120 000 m² de commerces, un signal symbolique fort à l'échelle de la ville de jour comme de nuit ;

• Être durable et vertueux. La résolution de ce paradoxe s'est faite, au-delà des multiples connexions d'espaces publics entre elles, par une étude plus large du territoire et de ses habitants. La compréhension des fils cachés du site et de ses alentours au cours du temps a pu ouvrir de nouvelles perspectives à la maîtrise 'œuvre. La compréhension que les éléments naturels (la rivière Parramatta) étaient des liants bien plus puissants et durables que les infrastructures humaines récentes, a permis à la maîtrise d'œuvre d'ouvrir des pistes de recherches et réflexions relatives au biomimétisme urbain, afin de construire des infrastructures durables. Ce projet a amené la maîtrise d'œuvre à revoir son processus de conception architectural et urbain en y intégrant à part entière l'étude et la connaissance du vivant.

Pôle d'Excellence du Biomimétisme Marin

Le PEBM invite à adopter un autre regard sur la conception de nos bâtiments. En s'inspirant de la certification Living Building Challenge le PEBM tend à être un bâtiment frugal qui n'utilise que peu de matériaux pour sa conception, un batiment low tech demandant la mise en œuvre de technologies simples ainsi qu'un bâtiment bas carbone visant à être régénératif.

Une étude complète à l'échelle du territoire a fait émerger des problématiques fortes sur la discontinuité des trames verte, brune et bleue alors même que le Pays-Basque est un des points chauds de la biodiversité au niveau mondial. Les contraintes fortes du site d'implantation (déclivité, implantation) ont fait émerger une opportunité de réunir techniques de construction vernaculaire et innovation durable valorisant une filière d'économie bleue à l'échelle de la Région Nouvelle Aquitaine. La recherche des espèces en danger localement a conduit l'équipe en charge de la conception du PEBM à formaliser les toitures du projet pour leur créer des habitats et des lieux propices à leur développement. La volonté de traiter naturellement le flux écologique de l'eau bien au-delà de notre terrain a guidé notre implantation. Ainsi, à l'image de notre modèle biologique qu'est l'éponge, le bâtiment se fond dans son environnement pour mieux se mettre à son service.

Le processus de conception biomimétique intégrant un conseil scientifique, a amené la maitrise d'œuvre à faire de très nombreuses itérations et à développer leurs connaissances sur de nombreux sujets. En retour, les connaissances non utilisées directement ont enrichi le processus permettant à la maitrise d'œuvre d'en améliorer les rouages. Un dernier résultat très intéressant réside certainement dans le choix de l'espèce biologique dont la seule référence a permis l'équipe en charge de la conception du PEBM de répondre à de nombreuses contraintes.

Residence solaire

Le résultat du calcul algorithmique phyllotaxique ne dessine pas un projet architectural, mais plutôt un ensemble de gabarits et d'implantations permettant la genèse d'un projet architectural qui pourra ainsi optimiser la capture de l'énergie solaire. Cet outil permet de développer une méthodologie d'optimisation de plans masses (implantations, volumétries, orientations et ouvertures des bâtiments d'un projet urbain) en phase amont de faisabilité architecturale ou urbaine. Ce projet est le plus avancé an terme de définition de projet et il montre que le calcul algorithmique permet une expression architecturale tout à fait singulière.

Skolkovo Innovation Center

Les conditions de vie aux pôles sont rudes : basses températures, présence de glaces terrestres et de mer, alternance marquée jour/nuit et faibles taux d'humidité. Les espèces vivantes qui s'y sont adaptées ont développé tout un arsenal de réponses morphologiques, physiologiques et comportementales, qui inspirent la technologie. Le succès de ce concept a aussi été dépend de l'acceptation des futurs habitants ; car la sensibilisation et l'éducation sont essentielles pour atteindre les objectifs de durabilité à long terme. Enfin, le biomimétisme montre sa capacité à s'exprimer à l'échelle urbaine. L'étude des écosystèmes — l'interconnexion entre espèces animales et végétales —, infuse la conception urbaine.

Tour D2

Ce projet a particulièrement démontré que le temps passé à faire des études ne représente pas un coût, mais un réel investissement, quand on en voit ici les effets positifs : un coût cible et un calendrier respecté. La tour a même été livrée avec 3 mois d'avance ! Aujourd'hui près de 7 ans après sa livraison, la tour D2 est une icône qui se démarque par son aspect organique et sa silhouette qu'aucune autre n'a. Elle crée véritablement le renouveau du paysage de La Défense parce qu'elle fait écho au vivant.
